# Human brain anatomy reflects separable genetic and environmental components of socioeconomic status

**DOI:** 10.1126/sciadv.abm2923

**Published:** 2022-05-18

**Authors:** Hyeokmoon Kweon, Gökhan Aydogan, Alain Dagher, Danilo Bzdok, Christian C. Ruff, Gideon Nave, Martha J. Farah, Philipp D. Koellinger

**Affiliations:** 1 Department of Economics, School of Business and Economics, Vrije Universiteit Amsterdam, 1081 HV Amsterdam, Netherlands.; 2 Zürich Center for Neuroeconomics (ZNE), Department of Economics, University of Zurich, 8006 Zürich, Switzerland.; 3 McConnell Brain Imaging Centre, Montreal Neurological Institute (MNI), McGill University, Montreal, QC H3A 2B4, Canada.; 4 Department of Biomedical Engineering, Faculty of Medicine, McGill University, Montreal, QC H3A 2B4, Canada.; 5 School of Computer Science, McGill University, Montreal, QC H3A 2A7, Canada.; 6 Mila-Quebec Artificial Intelligence Institute, Montreal, QC H2S 3H1, Canada.; 7 Marketing Department, the Wharton School, University of Pennsylvania, Philadelphia, PA 19104, USA.; 8 Center for Neuroscience & Society, University of Pennsylvania, Philadelphia, PA 19104, USA.; 9 La Follette School of Public Affairs, University of Wisconsin-Madison, Madison, WI 53706, USA.

## Abstract

Socioeconomic status (SES) correlates with brain structure, a relation of interest given the long-observed relations of SES to cognitive abilities and health. Yet, major questions remain open, in particular, the pattern of causality that underlies this relation. In an unprecedently large study, here, we assess genetic and environmental contributions to SES differences in neuroanatomy. We first establish robust SES–gray matter relations across a number of brain regions, cortical and subcortical. These regional correlates are parsed into predominantly genetic factors and those potentially due to the environment. We show that genetic effects are stronger in some areas (prefrontal cortex, insula) than others. In areas showing less genetic effect (cerebellum, lateral temporal), environmental factors are likely to be influential. Our results imply a complex interplay of genetic and environmental factors that influence the SES-brain relation and may eventually provide insights relevant to policy.

## Teaser

Socioeconomic status is linked with brain anatomy through a regionally varying balance of genetic and environmental influences.

## INTRODUCTION

Socioeconomic status (SES), typically measured by income, education, occupation, and neighborhood quality, is a powerful predictor of important life outcomes including physical and mental health, academic achievement, and cognitive abilities (*1*–*5*). The brain plays a central role not only in these relations, most obviously in mental health and intellectual capabilities, but also in physical health through neuroendocrine and inflammatory pathways (*6*, *7*). Thus, neuroscience provides a window on the biosocial pathways linking SES and human health and capabilities.

Neuroscience research on SES has revealed a generally positive relation with overall brain volume, as well as with regional cortical and subcortical volumes and cortical surface areas (*8*–*10*). We note variability across studies in the regions most associated with SES, which may be due, in part, to the relatively small samples studied, to differences in the ways SES has been measured and analyzed (e.g., choices of covariates) (*10*, *11*), and to different environments with different levels of assistance to individuals of low SES (*12*, *13*). One of the goals of the present study is to establish the relation of SES to regional gray matter volumes (GMVs) in the largest sample so far examined for voxel-level data, using a comprehensive measure of SES and controls for a number of potential confounds, based on a well-powered, preregistered analysis plan.

The second goal of the study is to differentiate genetic from environmental causes of the SES-GMV relation. The role of genes and environment in various outcomes associated with SES has been debated for decades and has provoked controversy in part because of perceived implications for policy (*14*).

Here, we pursue these two goals using data from the UK Biobank (UKB), a large-scale prospective epidemiological study of individuals aged 40 to 69 years at recruitment (*15*, *16*). We conducted voxel-based morphometry (VBM) analysis to investigate GMV associations with SES, which was measured by a rich set of SES indicators. To probe the genetic basis of the SES-GMV relation, we constructed a polygenic index of SES from multiple genome-wide association study (GWAS) results (effective *N* = 849,744), which included a large-scale meta-analysis of educational attainment (*17*). We then examined to which extent the estimated SES-GMV associations can be attributed to the shared common genetic architectures of SES.

## RESULTS

After selecting participants who had undergone both magnetic resonance imaging (MRI) and genotyping, and had complete SES information related to occupation, income, education, and neighborhood quality, we excluded participants with clinical diagnoses related to brain pathology, morbid obesity, heavy alcohol drinking, or low data quality. The resulting sample was 23,931 individuals, with a mean age of 62, 57% of whom were female. This sample size provides 90% statistical power to detect effects as small as *R*^2^ > 0.17% at the 5% significance level (corrected for multiple testing by permutation testing; uncorrected *P* < 2.19 × 10^−6^; see section S4.1). T1 images were preprocessed with Computational Anatomy Toolbox (CAT) 12, and anatomical regions were labeled according to the Neuromorphometrics atlas.

**Fig. 1.**
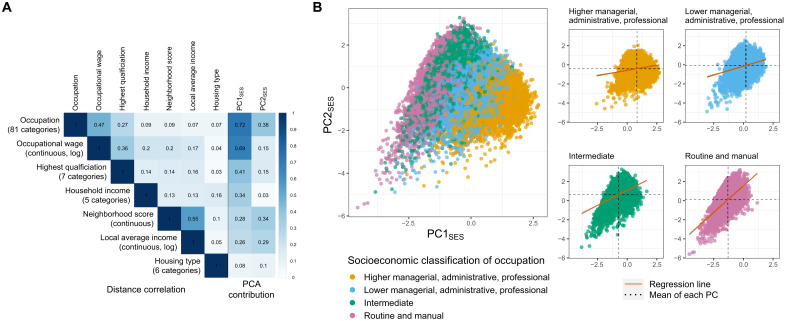
Measures of SES and PCA. (**A**) On the left, a distance correlation matrix is plotted for seven indices of SES. On the right, the squared loadings for each PC are indicated. (**B**) Scatter plots of the first PC (PC1_SES_) against the second component (PC2_SES_). The points in different colors represent four SES groups defined by National Statistics Socio-economic Classification, which are approximately clustered by the two PCs. On the right, the same scatter plots are presented for each SES group. The mean values of each PC are indicated for each group. The regression lines are plotted to describe that SES is more complex for the lower SES groups.

SES was represented in the analyses to follow by two summary measures, derived from available SES variables using a generalized version of principal components analysis (PCA; Fig. 1 and fig. S2). This approach better accommodates measurement error and allows us to appreciate the multidimensional nature of SES with just two components. PC1_SES_ mainly captures the positive correlations between the different SES measures and is most strongly influenced by occupations, occupational wages, and education. PC2_SES_ primarily reflects occupations and neighborhood qualities that are not strongly linked with educational attainment or income, e.g., individuals who live in relatively poor neighborhoods despite having high educational attainment. As shown later, PC2_SES_ contributes to capturing nongenetic variation in SES.

### SES and GMV

We first examined the relation between total intracranial volume (TIV) and SES by regressing TIV on PC1_SES_ and PC2_SES_, controlling for sex, age, genetic population structure, and a number of image-related technical covariates (see section S3.3). PC1_SES_ is positively associated with TIV {standardized β = 0.10, *P* = 1.1 × 10^−87^, 95% confidence interval (CI) [0.09, 0.11]}, while for PC2_SES_ the relation is statistically indistinguishable from zero (standardized β = 0.01, *P* = 0.14, 95% CI [−0.00, 0.02]). The two PCs together explain 1.6% of the variance of interest in TIV beyond the covariates (partial *R*^2^)—slightly higher than TIV’s relation to educational attainment (1.4%) and lower than its relation to fluid intelligence (2.6%) (*18*).

**Fig. 2.**
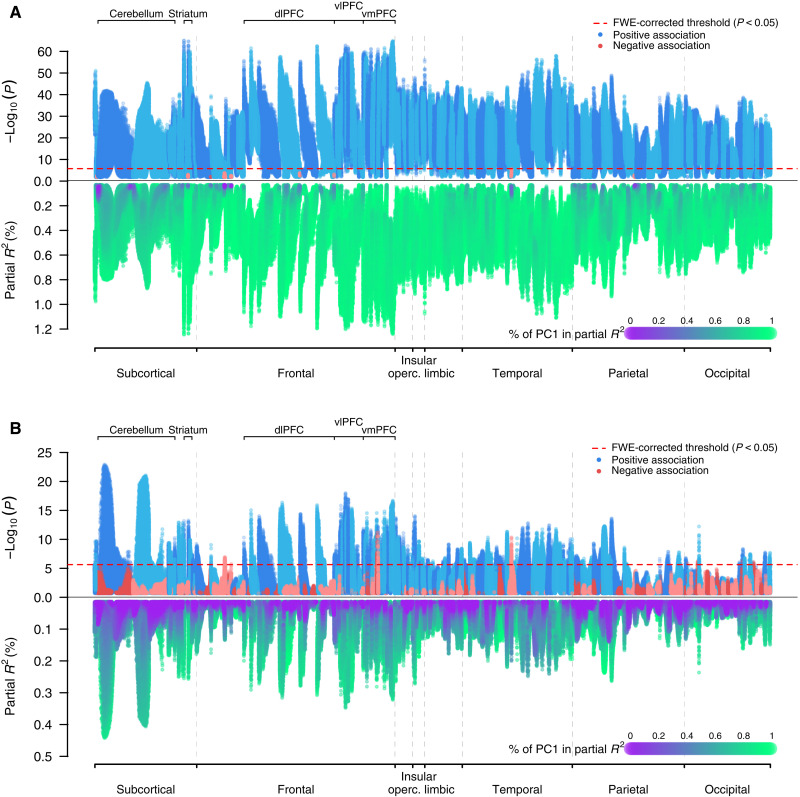
Manhattan plots: VBM of GMV and SES. (**A**) Univariate VBM results on the two PCs for SES. These regressions did not control for TIV. *P* values on a log_10_ scale (top) and partial *R*^2^ (bottom) are plotted for each voxel. The sign of the association is that of the first PC. The voxels were anatomically labeled according to the Neuromorphometrics atlas and grouped by the labeled regions. Within each region, the voxels were ordered by their distance to the medoid of their region. (**B**) Univariate VBM results with TIV controlled for.

Next, we conducted VBM analysis to test the association of these two PCs with regional GMV across the brain using the same set of covariates. Higher SES is associated with larger GMV across the brain (Fig. 2A). In total, 89.5% of the voxels have a statistically significant association with SES at a family-wise error (FWE) rate of 5%, all of which are positive. For statistically significant voxels, the average partial *R*^2^ is 0.4% and the highest is 1.2%, with the strongest associations in the left ventral striatum and the right frontal pole. Thus, the positive relation between total brain volume and SES arises from many relatively small sources of structural variation that are widespread across the brain.

Accordingly, when TIV is controlled for, just 8.5% of the voxels have a statistically significant association with SES and the average effect size in partial *R*^2^ is reduced by over half to 0.17% for the statistically significant voxels (see section S4.1.2). As shown in Fig. 2B, the strongest positive associations between SES and relative GMV fall in the prefrontal, insular, frontal opercular, lateral parietal, and lateral temporal regions, as well as in subcortical areas including the cerebellum, striatum, and thalamus. While SES-GMV associations are mainly driven by PC1_SES_, PC2_SES_ contributes relatively more in lateral temporal, cerebellar, and ventromedial prefrontal regions than in other regions (Fig. 2B and fig. S4A).

The regions implicated in these analyses include many reported in previous studies of SES and brain structure. While the cerebellum has not often been linked to SES, this may reflect its omission from many morphometric studies [but see (*19*), for a study of SES and cerebellar volume specifically, with positive findings]. Conversely, hippocampus volume is often noted to correlate with SES. Although this was also found in the present study, it was not among the strongest relations.

We also explored the influence of individual aspects of SES, such as education and income, by conducting a cluster-based analysis (figs. S8 and S9) as well as VBM on each measure separately (figs. S5 and S11). The overall pattern of results is similar, with years of schooling being most strongly associated.

SES-health relations are often stronger at lower levels of SES, where more extreme deprivation may impose unique effects on health (*20*, *21*), and this pattern is also seen in SES effects on the cortex in children (*22*). Stronger SES-GMV associations were found here in the lower SES participants of our sample as well (fig. S6) (*23*). Regionally, this is particularly apparent in the striatum (low SES, *N* = 15,611, max partial *R*^2^ = 0.65%, TIV adjusted; high SES, *N* = 8320, max partial *R*^2^ = 0.17%, TIV adjusted).

An alternative measure of the strength of the SES-GMV relation is the ability of aggregate GMV measures to predict SES. The small effect sizes for individual voxels do not imply that the association between SES and overall GMV structure is also small. To show this, we constructed brain-wide GMV scores to predict PC1_SES_ and PC2_SES_ via a stacked block ridge regression (*24*) with fivefold cross-validation. These scores predict Δ*R*^2^ = 4.9% (95% CI [4.4, 5.4]) of out-of-sample variation in PC1_SES_ and Δ*R*^2^ = 0.5% (95% CI [0.3, 0.7]) in PC2_SES_ (see section S4.2 for details).

### Genetic and environmental components of SES-GMV relation

The second question to be addressed is the contribution of genetic and environmental influence to the SES-GMV relations reported here. We approached this by first estimating the single-nucleotide polymorphism (SNP)–based heritability of SES and brain measures as well as the pairwise genetic correlations among them, which indicated that the genetic architectures of SES and brain structure are partly overlapping (section S6.1). We then constructed a polygenic index for SES (PGI_SES_) using the results of the GWAS. In view of the sensitivity of GWAS results to differences in ancestry, we derived the index from UKB participants of European ancestry only, excluding the scanned participants and other participants genetically related to them. The genetic data consisted of relatively common genetic variants (SNPs) with minor allele frequency ≥1%, which were related to educational attainment, occupational wages, household income, local average income, and neighborhood quality, combined using Genomic Structural Equation Modeling (SEM) (*17*, *25*) (effective *N* = 849,744). PGI_SES_ is strongly associated with PC1_SES_ (Δ*R*^2^ = 7.1%, *P* < 10^−300^) and weakly associated with PC2_SES_ (Δ*R^2^* = 0.02%, *P* = 0.03) (see section S3.4 for details). PGI_SES_ could then be used with images from participants of European ancestry (*N* = 20,799) to help discriminate genetic from environmental causes of GMV differences.

**Fig. 3.**
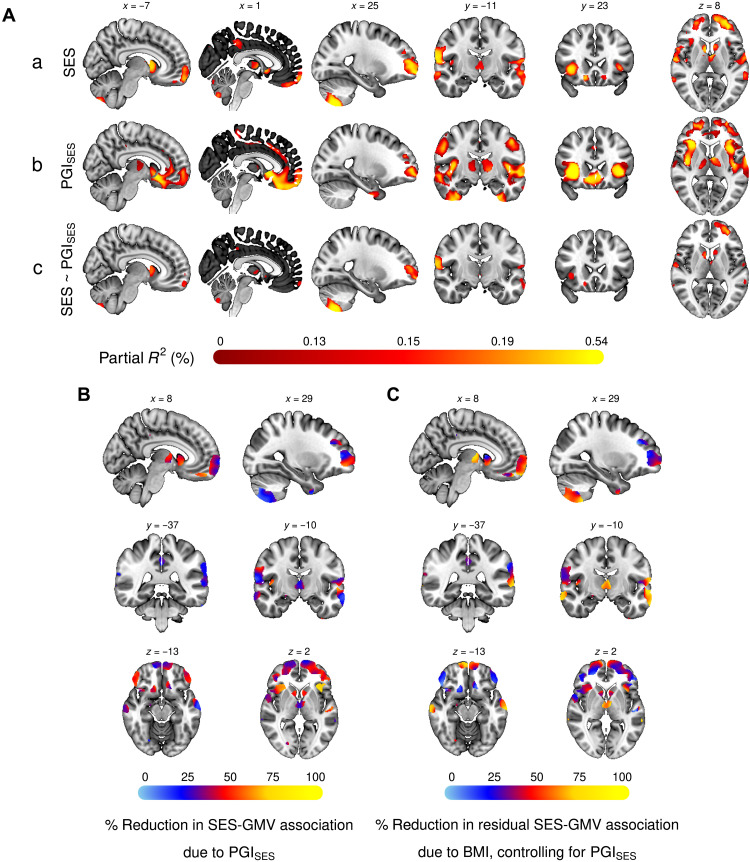
VBM of SES and its genetic and environmental components. (**A**) Univariate VBM results, with GMV as the dependent variable. Voxels significant at FWE rate of 5% level are plotted for (a) the two PCs measuring SES, (b) PGI_SES_, and (c) SES while controlling for PGI_SES_. (**B**) Percent reduction in the association between GMV and the two PCs for SES due to controlling for PGI_SES_. (**C**) Percent reduction due to controlling for BMI in the residual association between GMV and the two PCs for SES after controlling for PGI_SES_. The figures plot only voxels that had significant SES-GMV association before PGI_SES_ and BMI were controlled for. Montreal Neurological Institute (MNI) coordinates are indicated for (A) and (B). Measurement error in PGI_SES_ was adjusted for with genetic instrument variable regression for (B) and (C). The sample was restricted to individuals of European ancestry.

PGI_SES_ was then used to predict TIV (Δ*R*^2^ = 0.8%, *P =* 7.4 × 10^−64^) and GMV across the entire brain via VBM. The latter analysis revealed positive associations in widely distributed voxels (Fig. 3A, row b), with the most pronounced associations in the anterior insula, frontal operculum, prefrontal, anterior cingulate, and striatum. There is substantial overlap between the neuroanatomical correlates of SES and PGI_SES_. Controlling for TIV, approximately 41% of the GMV voxels associated with SES are also associated with PGI_SES_. This overlap is especially apparent in the insular and prefrontal cortices, with roughly 96 and 64% of the voxels associated with PC_SES_ also associated with PGI_SES_, respectively.

We then examined to which extent the shared common genetic architectures of SES and GMV account for the observed phenotypic associations by comparing TIV-adjusted regression results of GMV on SES with and without controlling for PGI_SES_. For 13% of the voxels significantly associated with SES before PGI_SES_ is controlled for, there is a statistically significant change in at least one of the coefficients for PC1_SES_ and PC2_SES_ after accounting for PGI_SES_. Controlling for PGI_SES_ reduces the SES-GMV associations across the entire brain, with the greatest reduction in the anterior insula, frontal operculum, ventrolateral prefrontal cortex, and ventral striatum of both hemispheres, consistent with VBM of PGI_SES_ mentioned earlier (Fig. 3B). When we correct for measurement error in PGI_SES_ using genetic instrumental variable (GIV) regression (*26*), we estimate that PGI_SES_ accounts for more than half of the SES-GMV associations for many of these regions. On average, 38% of the SES-GMV associations (min = 3%, max = 87%) can be statistically attributed to PGI_SES_ (see section S4.3 for details).

The remaining associations between GMV and SES could be either due to environmental influences on both or due to rare SNPs, structural variants (e.g., inversions and deletions), or interactions among genes (i.e., epistasis) that PGI_SES_ does not fully account for. Forty-three percent of the voxels significantly associated with SES fall into this category, remaining associated with SES after controlling for PGI_SES_ (Fig. 3A, row c). The SES-GMV association is least attenuated by genetic controls in the cerebellum and lateral temporal, lateral parietal, posterior cingulate, and primary motor regions, as well as some areas of the dorsolateral and ventromedial prefrontal cortex (vmPFC) and the thalamus. Controlling for PGI_SES_ accounts for less than 30% of the SES-GMV association in many of these regions. These results suggest that the aforementioned regions may be particularly susceptible to the influence of the socioeconomic environment. This is consistent with the relatively stronger association of PC2_SES_ to GMV in many of these areas, as PC2_SES_ was found to be barely heritable (see section S6.7). In summary, a substantial portion of the SES-GMV relation is attributable to known genetics, and that portion varies according to region of the brain. The remaining portion of this relation is also substantial and likely includes the effects of the environment.

Next, we sought to extend our evidence concerning environmental influences through the study of a specific environmental factor. Numerous environmental exposures are associated with SES and are plausible causal contributors to the SES-GMV relation found here. These include prenatal and childhood factors with lifelong effects, as well as adulthood exposures such as chronic life stress, nutritional status, physical exercise, environmental toxins, smoking, and other substance use. Experimental research with animals and human research with longitudinal, quasi-experimental, or experimental studies show that these are all capable of affecting the brain. On the basis of recent research with the same sample relating mid-life obesity to cognitive and brain aging (*27*), we chose to extend our analyses by including body mass index (BMI) as a marker for a set of behavioral factors that could mediate the SES-GMV relation, including nutrition, physical activity, and obesity, which can affect the brain through their downstream effects on blood pressure, blood lipids, glucose metabolism, and inflammation. In addition to the logical point that PGI_SES_ controls would account for genetic influences of BMI on the SES-GMV relation, there is also experimental evidence of SES affecting BMI through the environment: Increasing SES causes BMI to decrease (*28*).

**Fig. 4.**
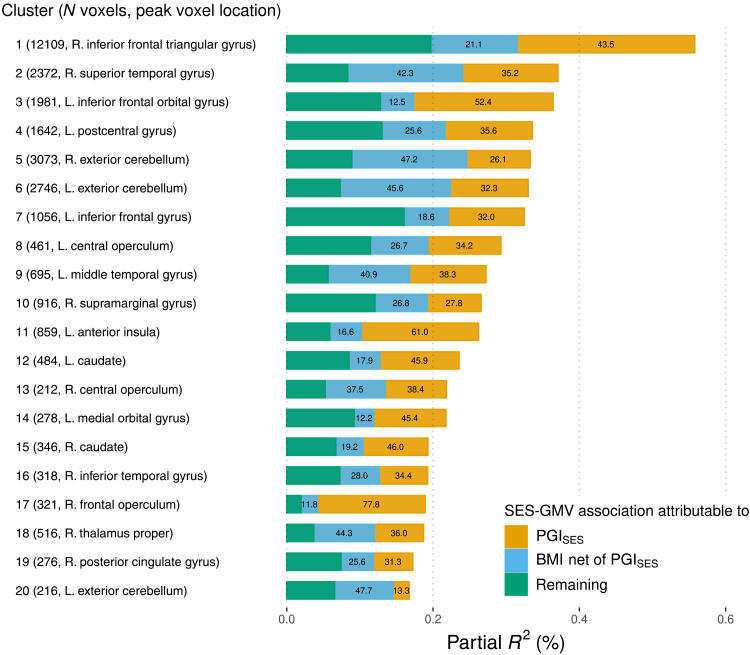
Genetic and environmental components in the association between SES and GMV of voxel clusters. Associations in partial *R*^2^ between the two PCs for SES and GMV in voxel clusters attributable to PGI_SES_ and BMI. The numbers in the bars report the percent share in the SES-GMV association statistically attributable to PGI_SES_ or BMI partialled out of PGI_SES_. The clusters were formed from the VBM results plotted in Fig. 3Aa. See table S9 for more information about the clusters. Measurement error in PGI_SES_ was adjusted for with genetic instrument variable regression. The sample was restricted to individuals of European ancestry.

BMI accounts for an average of 44% of the SES-GMV associations that remain after controlling for PGI_SES_ (Figs. 3C and 4). This result is not due to neurological disease associated with BMI, such as stroke or neurodegenerative disease, because neurological disease was an exclusionary criterion for our sample. The effect is particularly large in the thalamus and the cerebellum as well as the lateral temporal region and some areas of the vmPFC. Furthermore, for 91% of the voxels with significant SES-GMV association in the European ancestry sample, at least 50% of the estimated SES-GMV association can be statistically attributed to PGI_SES_ and BMI combined, with 67% on average.

**Fig. 5.**
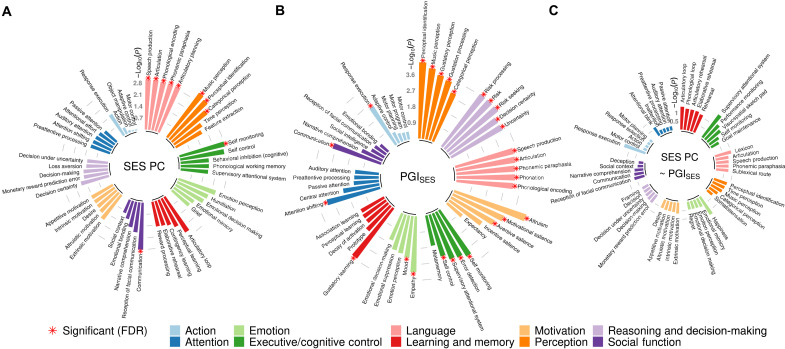
Functional annotation of brain regions associated with SES. A total of 492 cognitive concepts, belonging to 10 categories, were taken from Cognitive Atlas, and their predicted fMRI meta-analysis results were generated by NeuroQuery. For each concept, we computed the difference in mean chi-square between voxels statistically significant at nominal 1% level and the rest of the voxels. Then, a pseudo–*T* score for the difference was computed and its *P* value was obtained by 10,000 spatial permutations. This procedure was carried out for the VBM results for (**A**) two PCs of SES, (**B**) PGI_SES_, and (**C**) SES controlling for PGI_SES_. The *P* values of the top five concepts from each category were plotted on a log_10_ scale, where each category was ordered by the average of the top five concepts. The asterisk indicates the significance at the false discovery rate (FDR) of 5% level. See table S17 and fig. S15 for the full results.

We then explored the possible functional implications of the volumetric differences observed here by relating them spatially to the results of meta-analyzed functional MRI (fMRI) studies, based on NeuroQuery and 492 cognitive concepts from the Cognitive atlas knowledge base (Fig. 5 and fig. S15) (*29*, *30*). The neuroanatomical correlates of SES are most strongly expressed in language, perceptual cognitive functions, self-monitoring, and communication with statistical significance at the false discovery rate of 5%. These functional associations of SES appear to be driven by genetic influences (PGI_SES_), while PGI_SES_ also distinctly reflects functions related to decision-making (risk and uncertainty), altruism, and empathy as well as broader categories of concepts as shown. The regions presumed to be more environmentally susceptible (Fig. 5C) tend to relate more to functions pertaining to executive control and learning and memory, none of which, however, were statistically significant at the false discovery rate of 5%.

## DISCUSSION

In summary, our results show that SES is linked with brain anatomy through a regionally varying balance of genetic and environmental influences. The functions of the implicated brain regions span many cognitive and affective capacities. A measurement error–corrected polygenic index enabled us to separate regions whose correlations with SES can be partly attributed to common genetic variants, at least in individuals of European ancestry, from other regions more susceptible to environmental and behavioral exposures that correlate with SES, notably BMI. Our results suggest that brain health is more susceptible to SES-related environmental stressors in specific regions, including reduced GMV in the cerebellum among individuals with low SES.

Our study is not the first to introduce the genetic aspect in neuroscience of SES (*31*–*33*). Notably, global and regional measures of cortical regions have been found to have association with a polygenic index for educational attainment (*32*, *33*). Total surface area has also been shown to correlate independently with both parental education and a polygenic index for educational attainment (*33*). To our knowledge, our study is the first to show varying degrees of the genetic contribution to the relationship between SES and brain-wide regional measures, including subcortical regions. Specifically, we identified many regions that remained associated with SES even after adjusting for genetic controls (PGI_SES_).

In an age of growing inequality and socioeconomic disparities in health, achievement, and wellbeing, understanding the neural embedding of SES has social as well as scientific relevance. Poverty and social deprivation are associated with widespread regional reductions of GMV, which the present results confirm with unprecedented certainty and anatomical specificity. A novel implication of our findings is that this association can be explained in part, but only in part, by genetic predisposition to different degrees across the brain. It has been argued that genetically caused disadvantages cannot, at present, be ameliorated by policies that improve the social and economic environment (*34*). However, this reasoning is invalid for at least two reasons. First, even entirely genetic conditions can be treated with environmental interventions, for example, phenylketonuria (*35*). Second, genetic contributions to complex behavioral outcomes such as SES are likely to work via environmental channels that can be influenced (*36*, *37*). In particular, the variance captured by PGI_SES_ is expected to contain indirect genetic effects such as genetic nurture (*38*) that work via different family environments, including family-specific differences in child-rearing and neighborhood quality. An extensive note in section S5 concerns the interpretation and limitations of our results.

For policy purposes, genetic influences should not be taken as a sign of intractability (*36*, *39*). Rather, our findings imply that biological and social factors both contribute to neural disparities and that policy interventions may influence and interact with biological factors. While it would be premature to base specific policies on our results, future research in this direction could provide insights that can be translated into targeted interventions [see (*40*) for an in-depth discussion]. For example, further insights into whether cognitive stimulation during early life or anti-poverty policies (*13*, *41*, *42*) reduce neural disparities would be valuable.

## MATERIALS AND METHODS

### Sample description

We used publicly available data from the UKB, which recruited approximately 500,000 participants from the general population of the United Kingdom (*15*, *43*). Study participants were 40 to 69 years old at recruitment between 2006 and 2010. Our study sample originates from 40,681 individuals whose structural T1 MRI images were available in January 2020 (data field 20252). To derive voxel-level GMVs, we processed T1 images from 38,545 genotyped individuals with CAT12 for Statistical Parametric Mapping (SPM). We then applied several filters to ensure data quality and avoid spurious findings, which concern clinical diagnoses related to brain pathology, morbid obesity, heavy alcohol drinking, or low data quality. The details for these filters can be found in the Supplementary Materials.

After applying these exclusion criteria, 31,330 individuals remained in our sample. A total of 7215 individuals were further excluded due to missing data for the variables used in the analyses. To rule out that our results are influenced by shared family environments among related individuals, we also removed close relatives by randomly dropping one from each pair of siblings or parent-offsprings (see section S2 for details). Our final sample for the main analysis included *N* = 23,931 individuals. In the analyses that used genetic data, we included *N* = 20,799 individuals of European ancestry from this sample.

### Measures

#### Brain imaging data

We extracted GMV on the voxel level from T1-weighted structural brain MRI images provided in the NIFTI (Neuroimaging Informatics Technology Initiative) format (data field 20252). The scanning and processing protocols are detailed in the UKB’s brain imaging documentation (https://biobank.ctsu.ox.ac.uk/crystal/crystal/docs/brain_mri.pdf) as well as in publications (*15*, *44*). We preprocessed the T1 images with CAT12 for SPM (www.fil.ion.ucl.ac.uk/spm/software/spm12/). A detailed description on how we derived voxel-level GMV data is available in section S3.1.

#### SES measures

We collected and constructed an extensive set of SES measures: occupation, occupational wages, household income, housing type, local average household income, and neighborhood SES score. We derived some of the variables by relying on external data sources or aggregating several measures. Specific definitions of these measures can be found in section S3.2.

We then reduced the dimensions of the data by extracting the first two PCs, which represented overall SES implied by the available indicators. To account for the fact that we have both noncategorical and categorical SES indicators, we used a method that is often called factorial analysis of mixed data, which is essentially a generalization of PCA that can handle such mixed data (*45*, *46*). This method combines ordinary PCA for noncategorical data with multiple correspondence analysis for categorical data and is implemented in the R package PCAmix (*47*).

#### Control variables

As control variables, we included age, sex, TIV, genetic population structure, and a set of image-related technical covariates such as site and time of acquisition. The full description of control variables and their details can be found in section S3.3.

#### Polygenic index for SES

We constructed a PGI for SES (PGI_SES_) by combining multiple GWAS results of SES indicators, which included educational attainment, occupational wages, household income, local average income, and neighborhood score. We conducted GWAS on each of these measures with the UKB participants of European ancestry, excluding those in the analysis sample of this study as well as their close relatives (up to the third degree of relatedness). We ran each GWAS with a linear mixed model, estimated by BOLT-LMM (*48*). See section S3.4 for more details about these GWAS.

We combined these GWAS results to represent general SES by the common factor GWAS function of Genomic SEM (*25*). The effective sample size of this common-factor SES GWAS amounts to 849,744 (*49*). We then constructed the PGI for SES for those of European ancestry in the analysis sample (*N* = 20,799). To adjust for the correlation between the SNPs, we used a Bayesian approach called LDpred (*50*, *51*) with a reference panel from the Haplotype Reference Consortium (version 1.1) (*52*). The SNPs included in the PGI_SES_ were limited to the autosomal biallelic SNPs established by the International HapMap 3 Consortium (*53*), which are known to work well for phenotype predictions (*17*, *54*). The SNPs were also filtered to ensure minor allele frequency > 0.01, imputation score > 0.7, and missing rate < 0.05. As a result, 1,020,632 SNPs were used for PGI_SES_. PGI_SES_ was standardized to have zero mean and unit variance.

### Statistical analysis

A detailed description of the analysis methods used is available in the Supplementary Materials. Only the overall summary was provided here.

#### Voxel-based morphometry

Our baseline analysis estimated the associations between voxel-level GMV and the two SES PCs. For each voxel, GMV was regressed on the two SES PCs along with the control variables. An *F* test was conducted for each voxel to test whether there was significant association between its GMV and the SES PCs jointly. We used permutation testing to correct for multiple hypothesis testing across voxels and used *P* = 2.193 × 10^−6^ as the 5% significance level corrected for FWE (see section S4.1.3 for details).

#### VBM with PGI_SES_

Using PGI_SES_, we conducted the following additional VBM analyses: (i) VBM of SES PCs only with individuals of European ancestry, (ii) VBM of PGI_SES_, and (iii) VBM of the SES PCs controlling for PGI_SES_. These VBMs were carried out in the same way as the baseline analysis described above. We then examined which GMV voxels are significantly associated with the SES PCs and/or PGI_SES_. Furthermore, we tested whether there were statistically significant changes in SES-GMV associations before and after PGI_SES_ was controlled for, by using the Wald test (see section S4.3.2).

#### Measurement error correction for polygenic index

PGI_SES_ is a noisy proxy of true linear effects of common genetic variants that are linked to SES because GWAS estimates of individual SNP effects are obtained from finite sample sizes. The difference between the true PGI and the available PGI can be viewed as the classic measurement error, which leads to an attenuation bias in the coefficient estimate for the PGI_SES_. We addressed this attenuation bias by using GIV regression (*26*). The essential idea is that the true PGI_SES_ can be recovered from a noisy PGI_SES_^(1)^ by using another PGI_SES_^(2)^ as an instrumental variable that was derived from a different GWAS sample. The crucial assumption here is that the noise in PGI_SES_^(1)^ and PGI_SES_^(2)^ is uncorrelated to each other. GIV regression can address the measurement error in PGI_SES_ to the extent that this assumption holds.

To obtain PGI_SES_^(1)^ and PGI_SES_^(2)^, we randomly split the UKB GWAS sample into two subsamples (*N* = 105,517 to 170,945) such that each subsample has the same male-female ratio and no individuals in one subsample are related to anyone in the other subsample with more than the third degree of relatedness. With each subsample, GWAS was run for the five numerical SES measures and the results were combined with Genomic SEM. Then, PGI_SES_^(1)^ and PGI_SES_^(2)^ were constructed from one of the two independent GWAS subsample results (see section S4.3.4).

#### Functional annotations

We connected our anatomical findings to known functional localizations by leveraging Cognitive Atlas and the extrapolatable meta-analysis tool NeuroQuery (*29*, *30*). We first took the 518 cognitive concepts from Cognitive Atlas, which were categorized into 10 functional categories. Then, for each concept, we generated a meta-analyzed *z*-score brain map using NeuroQuery. This toolbox allows users to generate a predictive MRI-derived spatial distribution for any term, based on very large-scale meta-analyses containing mostly functional MRI studies. We excluded 26 concepts containing a term for which NeuroQuery failed to generate a brain map. As a result, 492 concepts remained. For each concept-associated brain map, we calculated the difference in mean chi-square between voxels statistically significant nominally at 1% level and the rest of voxels in the VBM results. We then computed a pseudo–*T* score for the difference in mean chi-square and obtained its *P* value from 10,000 spatial permutations of the *F* statistics map from the VBM. We used an approach developed by Burt *et al*. (*55*) for permutation, which allowed us to permute the volumetric brain map with subcortical regions while preserving spatial autocorrelation. We used these permutation-based *P* values as a summary measure to evaluate the strength of signal for a given functional concept in relation to SES (see section S4.4 for details).

## Supplementary Materials

### This PDF file includes:

Sections S1 to S7 Figs. S1 to S19 References

### Other Supplementary Material for this manuscript includes the following:

Data S1, containing tables S1 to S25

## Notes

View/request a protocol for this paper from *Bio-protocol*.
