# Supplementary material for: Human brain anatomy reflects separable genetic and environmental components of socioeconomic status
Source: Sci Adv. 2022 May 18;8(20):eabm2923. doi: 10.1126/sciadv.abm2923 (PMC9116589; doi:10.1126/sciadv.abm2923)
Supplement: Supplementary file 1 — Sections S1 to S7 Figs. S1 to S19 References [file sciadv.abm2923_sm.pdf]

Science Advances
AAAS

Supplementary Materials for

Human brain anatomy reflects separable genetic and environmental components of socioeconomic status

Hyeokmoon Kweon, Gökhan Aydogan, Alain Dagher, Danilo Bzdok, Christian C. Ruff, Gideon Nave, Martha J. Farah*, Philipp D. Koellinger*

*Corresponding author. Email: mfarah@upenn.edu (M.J.F.); koellinger@wisc.edu (P.D.K.)

Published 18 May 2022, *Sci. Adv.* **8**, eabm2923 (2022)
DOI: 10.1126/sciadv.abm2923

The PDF file includes:

Sections S1 to S7
Figs. S1 to S19
Legend for data S1
References

Other Supplementary Material for this manuscript includes the following:

Data S1, containing tables S1 to S25

1. Study overview

In this study, we aim to answer two research questions following a pre-registered analysis plan (https://osf.io/kg29c/):

(1) Are there robust associations between socio-economic status (SES) and brain anatomy?

(2) How much of the association between SES and brain anatomy is due to common genetic factors that are linked to SES?

To this end, we conducted voxel-based morphometry (VBM) analysis of grey matter volumes (GMV) on socioeconomic status (SES), using a population sample of 23,931 older adults from the UK Biobank (UKB) that contains brain images, genetic data, and several measures of SES (*15*, *16*).

Our phenotypic measures of SES uses all available information on SES that is available in the UKB and fully recognizes the multidimensional nature of SES. In particular, we measure SES as the first two principal components (PC) of available indices of household income, occupations, neighborhood, and education. These PCs were then jointly tested for their association with voxel-level GMVs in a univariate VBM. Permutation testing was used to maintain a familywise error rate of 5%.

To investigate the genetic basis of the estimated SES-GMV associations, we constructed a polygenic index for SES (PGI_{SES}) derived from multiple genome-wide association studies (effective N=849,744) and adjusted for measurement error in PGI_{SES} using genetic instrumental variable regression (*26*). We then examined to which extent the estimated SES-GMV associations can be attributed to the shared common genetic architectures of SES and the GMV structure by comparing regression results of GMV on SES before and after controlling for the polygenic index for SES.

Our analysis was carried out under the auspices of the Brain Imaging and Genetics in Behavioral Research Consortium (https://big-bear-research.org/).

2. Sample description

We used publicly available data from the UKB, which recruited ≈500,000 participants from the general population of the UK (*15*, *43*). Study participants were 40-69 years old at recruitment between 2006-2010. Our study sample originates from 40,681 individuals whose structural T1 MRI images were available in January 2020 (data field 20252). To derive voxel-level grey matter volumes, we processed T1 images from 38,545 genotyped individuals with the Computational Anatomy Toolbox (CAT) 12 for SPM (see Section 3.1 for details). We then applied several filters to ensure data quality and avoid spurious findings. We excluded:

- 24 individuals with mismatch between genetic (data field 22001) and self reported sex (data field 31)
- 1,818 individuals with clinical diagnoses related to brain pathology (including dementia, Alzheimer's, Parkinson's, and chronic degenerative neurological diseases, Guillan-Barré syndrome, multiple sclerosis, other demyelinating diseases, stroke or ischaemic stroke, brain cancer, brain haemorrhage, brain or intracranial abscess, cerebral aneurysm, cerebral palsy, encephalitis, epilepsy, head injury, infections of the nervous system, meningeal cancer, meningioma, meningitis, ALS, neurological injury or trauma, spina bifida, subdural haematoma, subarachnoid haemorrhage, or transient ischaemic attack; see the pre-registered plan for ICD10 codes - https://osf.io/kg29c)
- 2,705 individuals who were morbidly obese (BMI > 35)
- 2,427 individuals who were current heavy drinkers, where heavy drinking is defined as consuming more than 24 drinks per week for males and more than 18 drinks per week for females (*56*, *57*), which is defined as the sum of data fields 1568,1578,1588,1598, and 1608.
- 11 individuals with the image-quality rating (computed by CAT12) lower than "C"
- 230 individuals with a sample homogeneity measure (mean voxel correlation) lower than the 1% quantile (0.805 based on all 38,545 individuals). To compute the homogeneity measure, we first estimated a correlation matrix with elements being correlation estimates between individuals computed from voxel-level GMV. The mean voxel correlation corresponds to column-wise (or equivalently row-wise) averages of this matrix

After applying these exclusion criteria, 31,330 individuals remained in our sample. 7,215 individuals were further excluded due to missing data for the variables listed in Section 3. In order to rule out that our results are influenced by shared family environments among related individuals, we also removed close relatives by randomly dropping one from each pair of siblings or parent-offsprings. Our final sample for the main analysis included N = 23,931 individuals. In analyses that employed genetic data, we included N = 20,799 individuals of European ancestry from this sample.

3. Measures

3.1. Imaging-derived phenotypes (IDPs)

We extracted GMV on the voxel level from T1-weighted structural brain MRI images provided in NIFTI format (data field 20252). The UKB scanned the participants with a Siemens Skyra 3T scanner using a standard 32-channel head coil (Siemens Healthcare, Erlangen,

Germany) in three assessment centers (Cheadle, Newcastle, and Reading). The scanning and processing protocols are detailed in the UKB's brain imaging documentation (https://biobank.ctsu.ox.ac.uk/_crystal/crystal/docs/brain_mri.pdf) as well as in publications (*15*, *44*).

We first pre-processed the T1 images with the Computational Anatomy Toolbox (CAT) 12 for SPM (www.fil.ion.ucl.ac.uk/spm/software/spm12/). The images were corrected for bias-field inhomogeneities, tissue-segmented, spatially normalized to the MNI space with 1.5mm resolution by linear and non-linear transformations, and were modulated to ensure that the total amount of signal in the original image was preserved during spatial normalization. 8-mm Full-Width-at-Half-Maximum Gaussian kernel was then used to spatially smooth the pre-processed images. More details can be found in our pre-registered analysis plan (https://osf.io/kg29c/) as well as in the recent publications of BIG BEAR consortium (*56*, *57*).

Following the standard VBM procedures (see e.g. SPM/CAT12 http://www.neuro.uni-jena.de/cat12/CAT12-Manual.pdf), we decided to exclude all voxels from the VBM analyses that did not contain any or sufficient grey matter. To determine this, we first computed the average of all GMV images and then thresholded the average brain image at 250 GMV intensity units. The resulting binarized image was then applied as a pre-mask to all individual images. After applying a gray matter mask derived from the data and dropping the voxels unlikely to contain any gray matter (*56*), GMV estimates from 504,426 voxels were used in the analysis.

3.2. SES measures

The UKB offers a rich set of SES indicators, including education, income, occupation, and neighborhood quality. In order to make full and efficient use of the data, we took a data-driven approach to measure SES by extracting principal components (PC) that capture overall SES from all SES measures available in the UKB. Our approach can be summarized as follows: We (1) collected every available source of information relevant to SES in the UKB; (2) combined measures or derived new variables when possible or appropriate; (3) extracted PCs that represent a sparse, but accurate overall measure of SES; (4) and jointly tested for neuroanatomical association of these PCs based on an F-test.

There are several important reasons that motivated us to use this approach. First, it allowed us to take into full account the multidimensional nature of SES. While each SES dimension tends to share the same direction of correlation, there often are cases that do not agree with such correlation in reality: for instance, a plumber may have less education than a university lecturer, but may earn higher income. Furthermore, the quality of a neighborhood in which an individual lives is an important dimension of SES, but it may be imperfectly correlated with

education and income. Such complex aspects of SES cannot be represented by a single SES measure such as education or income alone.

Second, our data-driven approach is useful for efficiently testing for the association between SES and neuroanatomy by summarizing the available measures and thereby decreasing the multiple testing burden and increasing the statistical power of our analyses.

Third, this approach also makes it possible to use the detailed occupation data of the UKB to a fuller extent. Because it is difficult to handle many occupational categories in a single analysis, studies often use an aggregated summary of occupation by classifying occupations into a small number of predefined categories. One example is using the UK's National Statistics Socio-economic Classification, which reduces the occupation data to 3 or 8 classes. Such a predefined classification can discard potentially useful information and may not truly represent different levels of SES. A data-driven approach can efficiently reduce wide categorical data of occupation into lower continuous dimensions, while minimizing information loss.

Fourth, our approach addresses important limitations of educational attainment measures in the study sample. In the UKB, qualifications are reported in only six non-hierarchical categories, some of which cover a wide range of educational levels. Furthermore, participants were allowed to indicate multiple categories without a specific instruction, which led to a large degree of variation in responses. For this reason, we chose not to use years of schooling as often done (*17*), but instead determined the highest qualification for each participant in a data-driven way and used it as a categorical variable.

3.2.1. Available measures of SES in the UK Biobank

We collected and constructed an extensive set of SES measures as described below. We derived some of the variables by relying on external data sources or aggregating several measures. The participants visited the assessment center up to four times and brain images were taken during the third or fourth visit (the fourth visit was for repeated imaging of a subset of participants). While the data used here was primarily collected during the brain imaging visit at the assessment centers, we used the latest available information if a measure was missing from this visit.

- Occupation (81 categories) - fields 132, 20024, 22617
  - Job codes for the latest job held before the age of 65, coded in 3-digit UK standard occupational classification (SOC) 2000.
- Log occupational wages (continuous) - fields 132, 20024, 22617
  - Sex-specific average occupational wage matched to 4-digit SOC codes. The wage data are obtained from the UK's Office for National Statistics, averaged over 2002-2010.
- Household income (5 categories) - field 738
  - Average total household income before tax.
- Housing type (6 categories) - field 680

- e.g., own outright, own with mortgage, rent

- Log local average household income (continuous) - fields 20074, 20075
  - Derived by matching home locations to Middle-layer Super Output Areas. The income data are obtained from the UK's Office for National Statistics (England and Wales only)
- Neighborhood SES score (continuous) - fields 26411, 26412, 26414
  - Weighted average of three composite deprivation indices for income, employment, and education, constructed at the level of Lower-layer Super Output Areas (England only). The weights were derived by using the inverse of the correlation matrix. This method takes a weighted average of multiple outcomes such that outcomes highly correlated with each other are assigned less weight, while outcomes receive more weight if they are less correlated and therefore represent new information. See (*58*) for details.
- Highest qualification (7 categories) - field 6138
  - See the following description in section 3.2.1.1.

- *3.2.1.1. Highest qualification*

This subsection describes how we derived the highest qualification. During the assessment, participants were asked to choose qualifications that they have from the below options:

1 College or University degree
2 A levels/AS levels or equivalent
3 O levels/GCSEs or equivalent
4 CSEs or equivalent
5 NVQ or HND or HNC or equivalent
6 Other professional qualifications eg: nursing, teaching
7 None of the above

Because participants were able to choose multiple qualifications and also because the vocational category (NVQ or HND or HNC or equivalent) covers an extensive range of educational levels, it was not straightforward to determine the highest qualification for qualifications below college degree. Preferably, a better qualification should correspond to a better SES. We therefore used the following procedure to determine the rank of each qualification.

We first created a new categorical qualification variable that treats each combination of multiple choices as a unique response. Using the method described in Section 3.2.2, we extracted the first PC from this variable along with the rest of SES variables listed above. The average of the first PC was then computed for each qualification from a group of people who reported having that qualification. Note that these groups are not mutually exclusive as the individuals can belong to multiple groups. We then determined the SES-rank of qualifications based on these average PC scores. This approach yielded the following ranking:

1 College or University degree
2 A levels/AS levels or equivalent
6 Other professional qualifications eg: nursing, teaching
3 O levels/GCSEs or equivalent
5 NVQ or HND or HNC or equivalent

4 CSEs or equivalent
7 None of the above

The highest qualification was chosen for each individual according to this rank, which was then included as a categorical variable in the principal component analysis described below.

3.2.2. Data reduction by principal component analysis

We reduced the dimensions of the data by extracting PCs, which represent overall SES implied by the available indicators. Standard principal component analysis (PCA) is only suitable for non-categorical data. Thus, to account for the fact that we have both non-categorical and categorical SES indicators, we employed a method that is often called factorial analysis of mixed data, which is essentially a generalization of PCA that can handle such mixed data (*45*, *46*). This method combines ordinary PCA for non-categorical data with multiple correspondence analysis for categorical data and is implemented in the R package PCAmix (*47*).

Our purpose here was not a factor extraction that finds all relevant factors as typically done, but to exploit only the most meaningful variation in the UKB's SES data to facilitate efficient discovery of neuroanatomical correlates of SES. For this purpose, it was optimal to use the minimal number of PCs that could sufficiently capture the multidimensional nature of SES. Given this objective, we used the first two PCs ($PC1_{SES}$ and $PC2_{SES}$) as aggregate indicators of SES because these PCs were sufficient to explain the overall SES.

Figs. 1B and S2 clearly demonstrate that the first two PCs are both necessary and sufficient to reasonably differentiate major SES groups. The later PCs no longer appear to contribute to distinguishing different SES levels. $PC1_{SES}$ mainly distinguishes high and low SES groups and appears to reflect the positive correlation among different SES measures. $PC1_{SES}$ mostly loads individual differences in occupation, educational attainment, and income (Fig. 1A). On the other hand, $PC2_{SES}$ contributes more to explaining the residual variation in the lower SES groups and illustrates more subtle aspects of SES. $PC2_{SES}$ primarily reflects occupation and neighborhood qualities that are not strongly linked with educational attainment or income (Figs. 1A and S2 and Table S2).

Furthermore, the PCA results reveal the complex nature of SES within lower SES groups. Fig. 1B shows that the lower SES a group represents, the more positively correlated the two components are. While the highest SES group even has a lower mean value of $PC2_{SES}$ compared to that of the lower SES groups, relatively better-off individuals within the lower SES groups tend to have higher levels of both $PC1_{SES}$ and $PC2_{SES}$. These observations imply that the dimensions of SES are more complex in the lower SES groups. Overall, these results demonstrate the multidimensional nature of SES, which cannot be sufficiently described by a single SES measure.

Fig S3 plots the eigenvalues of the extracted PCs. $PC1_{SES}$ represents a dominantly large part of the variation from our SES measures (eigenvalue=2.77). The eigenvalues decrease substantially from $PC2_{SES}$, which nonetheless explains an important amount of variation (eigenvalue=1.44). While the eigenvalues of the third and fourth PCs are not very different from that of $PC2_{SES}$, these PCs do not appear to explain the meaningful variation in SES as shown earlier.

Prior to the analyses, we standardized $PC1_{SES}$ and $PC2_{SES}$ so that they have zero mean and unit variance.

3.3. Control variables

We used the following variables as baseline control variables.

- Age at brain scan (linear, squared, and cubed terms) - field 21003
- Sex - field 31
- Age (linear, squared, and cubed terms) × Sex
- Total intracranial volume - estimated from CAT12
- Site of acquisition (Cheadle, Reading, or Newcastle) - field 54
- A natural cubic spline function of acquisition date (number of days when the acquisition happened since the acquisitions started) with 3 degrees of freedom - field 53
- Time of test (in seconds) - field 21862
- Interaction terms of acquisition site with all of the above
- The first 40 PCs of the genetic data - field 22009
- Genotyping array (UK BiLEVE or UK Biobank Axiom array) - field 22000

The acquisition date and time were included as control variables based on a recent paper (*59*), indicating that these variables account for subtle differences in the UKB's assessment protocols over time. For instance, Fig S1 demonstrates that there is a subtle temporal pattern over time since the UKB started collecting MRI images. We used a natural cubic spline function in order to capture highly non-linear patterns flexibly (the analysis plan specified 5 degrees of freedom for this, but we used 3 degrees of freedom due to rank deficiency). The first 40 genetic PCs were also used to control for the genetic population structure and the genotyping array to control for potential confounds in the genetic PCs due to different arrays used. The genetic PCs were derived internally by the UKB from unrelated individuals of mixed ancestries.

It is important to note that psychological characteristics that are correlated with SES, such as cognitive ability and mental health status, were not covaried. Our reasoning was that correcting for these traits would result in findings less typical of higher and lower SES. By analogy, consider covariates appropriate for assessing sex differences in the brain. Sports participation is more common in men than women throughout the lifespan, for reasons of biology and culture, and would also be expected to impact brain structure though cardiovascular and other mechanisms.

However, one might wonder how and whether correcting for sports participation distorts our understanding of sex differences. Our primary interest is presumably not in comparing men who play less sport than typical for their sex to women who play more, but rather men and women behaving according to their motivations and abilities. Returning to SES and associated psychological traits, here we have opted to focus on brain structure in higher and lower SES, rather than on particularly smart and well-adjusted low-SES individuals and particularly less smart and well-adjusted high-SES individuals.

3.4. Genome-wide association studies and construction of the polygenic index for SES

As a measure of genetic variation associated with SES, we used a polygenic index (PGI) that additively summarizes the effects of more than 1 million genetic markers. The genetic markers used here are single nucleotide polymorphisms (SNP), which are the most common form of genetic variation. A PGI s_i of individual *i* is a weighted sum of SNPs:

$$s_i = \sum_{j=1}^{M} \hat{\beta}_j x_{ij}$$

where x_{ij} represents the genotype of individual *i* for SNP *j* coded as the count of the reference allele. We estimated the weights $\hat{\beta}_j$ from genome-wide association studies (GWAS), which conduct univariate regressions of an outcome on each SNP across the genome. The resulting estimates were then adjusted for the correlation between the SNPs to obtain the weights $\hat{\beta}_j$.

We constructed a PGI for SES (PGI_{SES}) by combining multiple GWAS results of SES indicators, which included educational attainment, occupational wages, household income, local average income, and neighborhood score (see further details below). We conducted GWAS on each of these measures with the UKB participants of European ancestry, excluding those in the analysis sample of this study as well as their close relatives (up to the third degree of relatedness, which corresponds to everyone in the relatedness table reported by the UKB (minimum kinship coefficient = 0.04). We ran each GWAS with a linear mixed model, estimated by BOLT-LMM (*48*).

Educational attainment (years of schooling) was coded in the same way as the recent large-scale GWAS (*17*). *Household income* was coded as the natural log of the midpoint income of each income bracket (for the lowest and highest brackets, which are open-ended, 3/4 times the upper bound and 4/3 times the lower bound were used as the midpoint, respectively). The remaining indicators were the same as described in Section 3.2.1. Except for educational attainment, GWAS was run on male and female samples separately and the male and female results of each measure were meta-analyzed by the meta-analysis version of MTAG (*60*) to

account for possible sex heterogeneity in socio-economic outcomes. Here MTAG was used especially because it is robust to the relatedness between the samples. MTAG can be viewed as a generalization of the conventional inverse-variance-weighted meta-analysis. The sample sizes for each measure varied from 250,865 to 401,026 participants. For educational attainment, the GWAS result was meta-analyzed with the existing GWAS meta-analysis result of educational attainment (*17*), which excludes the UKB. More details of these GWAS are summarized in Table S4.

Finally, we combined these GWAS results to represent general SES by the common-factor GWAS function of Genomic SEM (*25*). The effective sample size of this common-factor SES GWAS amounts to 849,744 (*49*). We then constructed the PGI for SES for those of European ancestry in the analysis sample (N = 20,799). To adjust for the correlation between the SNPs, we used a Bayesian approach called LDpred (*50*, *51*) with a reference panel from the Haplotype Reference Consortium (version 1.1) (*52*). The SNPs included in the PGI_{SES} were limited to the autosomal bi-allelic SNPs established by the International HapMap 3 Consortium (*53*), which are known to work well for phenotype predictions (*17*, *54*). The SNPs were also filtered to ensure minor allele frequency > 0.01, the imputation score (INFO) > 0.7, and the missing rate < 0.05. As a result, 1,020,632 SNPs were used for PGI_{SES}. The PGI_{SES} was standardized to have zero mean and unit variance.

PGI_{SES} predicts about ΔR^2=7.1% of the variation of $PC1_{SES}$ out-of-sample among individuals of European ancestries, above and beyond the control variables (age, age^2, age^3, sex, interactions between sex and the age terms, genotyping array, and the first 40 genetic PCs). On the contrary, PGI_{SES} barely predicts $PC2_{SES}$, explaining ΔR^2=0.02% of its variation.

4. Statistical analyses

4.1. Voxel-based Morphometry (VBM) analysis

4.1.1. Baseline analysis

Our baseline analysis estimated the associations between voxel-level GMV and the two SES PCs. For each voxel j, we estimated the following regression model via ordinary least square (OLS):

$$(1)\quad GMV_i^j = \beta_1^j PC1_{SES,i} + \beta_2^j PC2_{SES,i} + Z_i^T \gamma^j + \varepsilon_i^j$$

where the GMV of voxel j is regressed on the two SES PCs. The vector Z_i include the control variables listed in Section 3.3. ε_i^j is the error term. The GMV and the SES PCs were standardized to have zero mean and unit variance. An F-test was used for each voxel to test whether there is

significant association between voxel *j*'s GMV and the SES PCs jointly with the the null hypothesis $\beta_1^j = \beta_2^j = 0$. We measured the association size by the variance of interest in GMV explained by the SES PCs beyond the covariates of no interest, *i.e.*, partial $R^2 := (R^2_{PC+Z} - R^2_Z) / (1 - R^2_Z)$. R^2_{PC+Z} is the R^2 from the unrestricted model, which includes the two SES PCs and the covariates of no interest, and R^2_Z is the R^2 from the restricted model, which only includes the covariates of no interest. We also quantified the relative contribution of $PC1_{SES}$ in the overall association size by $(R^2_{PC1+Z} - R^2_Z) / (R^2_{PC+Z} - R^2_Z)$. We used permutation testing to correct for multiple hypothesis testing across voxels (see Section 4.1.3 for details).

After the estimation, we anatomically labeled the voxels using the Neuromorphometrics atlas provided in CAT12 (http://Neuromorphometrics.com). For a summary purpose, we also generated cluster-based estimates. Each cluster consists of at least 200 neighboring voxels within the lobe (limbic, cerebellum, insular, frontal, parietal, occipital, temporal) which are significant at the familywise error rate of 5% in the baseline model. We then repeated the same analysis with mean GMV of these clusters.

4.1.2. Controlling for total intracranial volume (TIV)

Analyses that aim to identify associations between localized GMV and outcomes typically control for TIV, since volumetric brian measures scale with the head size. However, controlling for TIV as a linear covariate has important statistical implications for identifying localized GMV patterns linked to SES, because TIV is positively correlated with both SES and regional GMV. In Fig. 2B, GMV of some voxels appear to have negative association with SES when the TIV is included as a control variable in the model. On the contrary, Fig. 2A shows that almost all the voxel-level GMV are positively associated with SES when TIV is not controlled for. This result indicates that the absolute GMV-SES association is unlikely to be negative in any brain region.

To formally illustrate this point, consider a VBM model for SES with only the TIV as a covariate without loss of generality:

$$(2)\quad GMV_i = \beta_{\sim TIV} SES_i + \gamma TIV_i + \varepsilon_i$$

where GMV_i is the GMV of some voxel and $\beta_{\sim TIV}$ denotes the association between the voxel's GMV and SES while TIV is accounted for. Each variable is standardized to have zero mean and unit variance without loss of generality. γ corresponds to the association between the GMV and the TIV, conditional on SES. The linear dependence between the TIV and SES can be described

as: $E[TIV_i|SES_i] = \lambda SES_i$. If we denote β as the coefficient of SES from the regression of the GMV on SES without the TIV as a covariate, $\beta_{\sim TIV}$ can be written as:

$$(3)\quad \beta_{\sim TIV} = \beta - \lambda\gamma$$

Therefore, if both λ and γ are positive and large, $\beta_{\sim TIV}$ can be negative even when β is positive. Our data suggests that this is indeed the case: With the baseline model, we estimated $\hat{\lambda} = 0.10$ for $PC1_{SES}$ and $\hat{\lambda} = 0.01$ for $PC2_{SES}$. $\hat{\gamma}$ was on average 0.46 with the minimum=0.11 (right exterior cerebellum) and the maximum=0.72 (left gyrus rectus). Since estimates of β are positive for the vast majority of the voxels, one cannot conclude that the absolute GMV-SES association is truly negative even when estimates of $\beta_{\sim TIV}$ are negative. Instead, such negative estimates are evidence that λγ is large relative to β and that the GMV-SES association is essentially very small or non-existent for these regions. Note that here we did not interact TIV with the site of acquisition for simplicity when obtaining these estimates. There was not much difference in TIV due to images taken in different acquisition sites.

Therefore, caution is warranted when interpreting the results when TIV was adjusted for as a covariate. For this reason, we reported the VBM results both with or without TIV included as a covariate. Furthermore, given the above, our results suggest that SES is associated with greater gray matter across almost all brain regions investigated, despite small exceptions with negative estimates after adjusting for the TIV. Note that TIV was always included as a control variable unless otherwise stated.

4.1.3. Multiple testing correction

To correct for multiple testing across voxels, we used permutation testing to determine a *p*-value threshold that controls the familywise error (FWE) rate of 5% (*61*). Following a comprehensive simulation study that examined several permutation approaches for the brain-imaging (*62*), we applied the method developed by Freedman and Lane (1983) to construct an empirical distribution of test statistics (*63*). Consider an $N \times M$ matrix *Y* where column *j* is a length-*N* vector of voxel *j*'s GMV with M the number of the voxels. Each column was first residualized of the covariates of no interest (Z_i). Matrix *Y* was then permuted row-wise so that the correlation structure among the voxels was preserved. We then regressed each of the permuted GMV on the non-permuted, original regressors and recorded the maximum *F*-statistic. We repeated this process 5,000 times to form a distribution of the maximum *F*-statistics. We used the *p*-value

computed from the 95th percentile of this distribution (F = 13.04) as the p-value threshold for 5% FWE-corrected significance level, which corresponds to $p = 2.193 \times 10^{-6}$ (uncorrected).

While in principle the permutation testing has to be performed for each different analysis, the resulting p-value thresholds differed only marginally and the threshold for the baseline model was the most conservative. Therefore, we used the 2.193×10^{-6} threshold for every voxel-based analysis.

4.1.4. Stratified analysis of high and low SES groups

To investigate potential heterogeneity across different SES groups, we conducted the same VBM analysis separately on high and low SES groups. High and low SES groups were defined by National Statistics Socio-economic Classification of the UK (*23*): high SES group holds a managerial, administrative, or professional occupation and low SES group holds intermediate, routine, or manual occupation ($N_{high} = 15{,}611$, $N_{low} = 8{,}320$).

4.1.5. VBM of Individual SES measures

To gain additional insight into the neuroanatomical correlates of SES, we conducted additional VBM analyses on each of the five individual numerical SES measures used to construct the SES PGI, described in Section 3.4. Note that the main purpose of these analyses was not to discover novel neuroanatomical correlates from each SES measure, but rather to compare neuroanatomical correlates across these measures.

4.2. Estimating the overall association between SES and GMV structure

Our VBM results demonstrate that the association between SES and an individual GMV IDP is small and does not exceed partial R^2 of 1% with TIV adjusted for. One might then ask how large the brainwide association between SES and the gray matter structure is if we can aggregate individual SES-GMV association estimates from individual voxels. Estimating the overall association is not an easy task because of the high dimension of the voxel-level GMV data and the strong spatial correlation among the voxels. We addressed these challenges by constructing a brainwide GMV score for SES with a machine learning technique. We used a stacked block ridge regression approach inspired by a recent whole-genome regression method (*24*). This approach allows us to tackle the high dimension issue by stacked regressions and the spatial correlation by the use of ridge regressions without excessive computational burden. Ridge regressions also ensure that we only capture linear relationships between SES and the GMV structure.

We constructed a brainwide GMV score for each SES PC in two steps:

(1) Voxels were first partitioned into blocks of 10,000 adjacent voxels. For each block, we ran a ridge regression of each SES PC on its 10,000 voxel-level GMVs with arbitrarily-chosen varying shrinkage parameters: {100, 100^2, 100^3}. We then computed predictions for each SES PC for each value of the shrinkage parameters, resulting in 3 predictors for each SES PC from each block. This resulted in 153 predictors from 51 blocks partitioned from 504,426 voxels.

(2) After collecting the predictors from all the blocks, a ridge regression was run on them together again. The prediction from this regression was used as a brainwide GMV score.

Both steps were implemented in 5-fold nested cross-validation: In the outer loop, the sample was split into 20% test set and 80% training set, the latter of which was again split into 20% validation set and 80% training set in the inner loop. In the inner loop, cross-validation was used to tune the shrinkage parameter for the step-2 ridge regression. The outer loop was used to train the final model and obtain predictions for the test set given the obtained value of the shrinkage parameter from the inner loop. We ensured that no information from the test set was used in the model training.

To measure the overall association between each SES PC and the GMV structure, we used a change in R^2 after including the corresponding brianwide GMV score to the regression. The covariates used were age, age^2, age^3, sex, interactions between sex and the age terms, TIV, genotyping array, and the top 40 genetic principal components. We computed confidence intervals with 1,000 bootstrapped samples.

Of note, we do not claim here that this approach is the best way of constructing a brainwide score or estimating the brainwide association. The primary goal of this analysis is to demonstrate that SES is associated with GMV structure to a substantial degree.

4.3. Incorporating genetics

4.3.1. VBM with PGI

Using PGI_{SES}, we conducted the following additional VBM analyses: (1) VBM of SES PCs only with individuals of European Ancestry (2) VBM of PGI_{SES} (3) VBM of the SES PCs controlling for PGI_{SES}. These VBMs were carried out in the same way as the baseline analysis detailed in Section 4.1. We then examined which GMV voxels are significantly associated with the SES PCs and/or the PGI and examined changes in SES-GMV associations before and after the PGI was controlled for. Note that we measured partial R^2 of the PCs for VBM of the SES PCs controlling for PGI_{SES} as $(R^2_{PC+PGI+Z} - R^2_{PGI+Z}) \,/\, (1 - R^2_Z)$ to be able to compare it with partial R^2 from VBM of SES PCs. In

addition to probing the difference in statistical significance after the PGI was controlled for, we directly tested whether controlling for the PGI significantly altered the SES-GMV association.

4.3.2. Testing differences in SES-GMV associations with and without PGI as a control variable

We used a Wald test to examine whether there was a significant difference in the SES-GMV association before and after the PGI_{SES} was controlled for. More specifically, consider a model where PGI_{SES} is added to the model (1) and also set up an auxiliary regression of the PGI on the SES PCs and the covariates for each voxel:

$$(4)\ GMV_i^j = \tilde{\beta}_1^j PC1_{SES,i} + \tilde{\beta}_2^j PC2_{SES,i} + \theta^j PGI_i + Z_i^T \tilde{\gamma}^j + \tilde{\varepsilon}_i^j$$

$$(5)\ PGI_i = \delta_1 PC1_{SES,i} + \delta_2 PC2_{SES,i} + Z_i^T \psi + u_i$$

Using vector notations: $\beta^j = [\beta_1^j\ \beta_2^j]^T$, $\tilde{\beta}^j = [\tilde{\beta}_1^j\ \tilde{\beta}_2^j]^T$, $\delta = [\delta_1\ \delta_2]^T$, which are all length-2 vectors, it can be shown:

$$(6)\ \beta^j - \tilde{\beta}^j = \theta^j \cdot \delta = \Delta^j$$

Therefore, the vector Δ^j represents the difference in the SES-GMV association for voxel *j* due to controlling for PGI_{SES}. Δ^j can be estimated as the product of estimates of $\hat{\theta}^j$ and $\hat{\delta}$ from the model (4) and (5), respectively. A Wald test was then used to test the null $\Delta^j = 0$ with the test statistic: $\hat{\Delta}^{jT}\, \hat{Var}(\hat{\Delta}^j)^{-1} \hat{\Delta}^j \sim \chi^2_2$, where $\hat{Var}(\hat{\Delta}^j)$ was approximated by the delta method: $\hat{Var}(\hat{\Delta}^j) \approx \hat{Var}(\hat{\theta}^j)\hat{\delta}^T\hat{\delta} + \hat{\theta}^{j2}\, \hat{Var}(\hat{\delta})$. Note that this analysis is statistically equivalent to a mediation analysis with PGI_{SES} being a mediator (*64*). We conducted this test only for the voxels whose GMV was significantly associated with the PCs. Then, the multiple testing was corrected for using Bonferroni correction (the corrected 5% threshold = 1.46×10^{-6} with 34,188 tests).

4.3.3. Measuring differences in SES-GMV associations with and without PGI as a control variable

To represent the relative size of Δ^j in relation to partial R^2, we used the relative change in the net variation explained by the SES PCs after adding PGI_{SES} to the model with the covariates of no interest: $[(R^2_{PC+Z} - R^2_Z) - (R^2_{PC+PGI+Z} - R^2_{PGI+Z})] / (R^2_{PC+Z} - R^2_Z)$. This measure is bounded between

0 and 1 as long as the sign of the coefficients for $PC1_{SES}$ and $PC2_{SES}$ do not change after controlling for PGI_{SES}. This expression can be interpreted as the percent change in the SES-GMV associations due to controlling for PGI_{SES} and essentially the part of the SES-GMV association that can be attributed to PGI_{SES}. Note that, because $PC2_{SES}$ is barely predicted by PGI_{SES} and even barely heritable (Table S5), the percent change in SES-GMV association after controlling for PGI_{SES} is essentially due to the change in $PC1_{SES}$-GMV association. We can therefore rewrite the earlier expression as:

$$(7)\quad [(R^2_{PC+Z} - R^2_Z) - (R^2_{PC+PGI+Z} - R^2_{PGI+Z})] \,/\, (R^2_{PC+Z} - R^2_Z)$$
$$\approx [(R^2_{PC1+Z} - R^2_Z) - (R^2_{PC1+PGI+Z} - R^2_{PGI+Z})] \,/\, (R^2_{PC+Z} - R^2_Z)$$
$$= \Delta_{PC1} \,/\, (R^2_{PC+Z} - R^2_Z)$$
$$= [\Delta_{PC1} \,/\, (R^2_{PC1+Z} - R^2_Z)] \times [(R^2_{PC1+Z} - R^2_Z) \,/\, (R^2_{PC+Z} - R^2_Z)]$$

where $\Delta_{PC1} = (R^2_{PC1+Z} - R^2_Z) - (R^2_{PC1+PGI+Z} - R^2_{PGI+Z})$, the change in the net variance explained by $PC1_{SES}$ after controlling for PGI_{SES}. Hence, the percent change in SES-GMV association is roughly the product of the percent change in $PC1_{SES}$-GMV association ($\Delta_{PC1} \,/\, (R^2_{PC1+Z} - R^2_Z)$) and the relative contribution of $PC1_{SES}$ in the overall SES-GMV association ($(R^2_{PC1+Z} - R^2_Z) \,/\, (R^2_{PC+Z} - R^2_Z)$). A larger share of SES-GMV association can be attributed to PGI_{SES} if genetic factors linked to SES play a bigger role for $PC1_{SES}$-GMV association and/or if $PC2_{SES}$ contributes relatively less to the overall SES-GMV association.

4.3.4. Measurement error correction for PGI

PGI_{SES} is a noisy proxy of true linear effects of common genetic variants that are linked to SES because GWAS estimates of individual SNP effects are obtained from finite sample sizes. The difference between the true PGI and the available PGI can be viewed as the classic measurement error, which leads to an attenuation bias in the coefficient estimate for the PGI_{SES}. Nonetheless, it is still possible to account for the linear effects of common genetic variants that the true PGI_{SES} would capture under reasonable assumptions. We addressed this attenuation bias by using genetic instrumental variable (GIV) regression (*26*). The essential idea is that the true PGI_{SES} can be recovered from a noisy $PGI_{SES}^{(1)}$ by using another $PGI_{SES}^{(2)}$ as an instrumental variable that was derived from a different GWAS sample. The crucial assumption here is that the noise in $PGI_{SES}^{(1)}$ and $PGI_{SES}^{(2)}$ is uncorrelated to each other. GIV regression can address the measurement error in PGI_{SES} to the extent that this assumption holds.

To obtain $PGI_{SES}^{(1)}$ and $PGI_{SES}^{(2)}$, we randomly split the UKB GWAS sample into two subsamples (*N*=105,517~170,945) such that each subsample has the same male-female ratio and no individuals in one subsample are related to anyone in the other subsample with more than the third degree of relatedness. With each subsample, GWAS was run for the five numerical SES measures and the results were combined with Genomic SEM as described in Section 3.4. Then, $PGI_{SES}^{(1)}$ and $PGI_{SES}^{(2)}$ were constructed from one of the two independent GWAS subsample results in the main imaging sample.

Using $PGI_{SES}^{(1)}$ and $PGI_{SES}^{(2)}$, we fitted the model (4) by the GIV estimation, which is two-stage least squares (TSLS).

$$(8)\quad GMV_i^j = \tilde{\beta}_1^j PC1_{SES,i} + \tilde{\beta}_2^j PC2_{SES,i} + \theta^j PGI_i^{(1)} + Z_i^T \tilde{\gamma}^j + \tilde{\varepsilon}_i^j$$

where $PGI_i^{(1)}$ is the PGI estimated from the first subsample. The first-stage equation can be written as:

$$(9)\quad PGI_i^{(1)} = \alpha_1 PGI_i^{(2)} + \alpha_2 PC1_{SES,i} + \alpha_3 PC2_{SES,i} + Z_i^T \eta + e_i$$

where the PGI estimated from the second subsample, $PGI_i^{(2)}$, is used as an instrument for $PGI_i^{(1)}$. We obtained the TSLS estimates by fitting the following equation:

$$(10)\qquad GMV_i^j = \tilde{\beta}_1^j PC1_{SES,i} + \tilde{\beta}_2^j PC2_{SES,i} + \theta^j \hat{PGI}_i^{(1)} + Z_i^T \tilde{\gamma}^j + \tilde{\varepsilon}_i^{*j}$$

where $\hat{PGI}_i^{(1)}$ is the fitted value from the equation (8). The statistical inference was then conducted but in the standard TSLS framework to test the association between the GMV and SES for each voxel conditional on PGI_{SES} (*65*).

We computed Partial R^2's based on adding or excluding $\hat{PGI}_i^{(1)}$ in model (10) instead of the unadjusted PGI. Similarly, we measured the difference in SES-GMV association after controlling for the PGI by GIV as $[(R^2_{PC+Z} - R^2_Z) - (R^2_{PC+\hat{PGI}^{(1)}+Z} - R^2_{\hat{PGI}^{(1)}+Z})] \;/\; (R^2_{PC+Z} - R^2_Z)$

4.4. Functional annotations

We connected our anatomical findings to known functional localizations by leveraging Cognitive Atlas and the extrapolatable meta-analysis tool NeuroQuery (*29*, *30*). We first took the 518 cognitive concepts from Cognitive Atlas which were categorized into 10 functional categories (taken from https://www.cognitiveatlas.org/concepts/categories/all on 2 July 2021). Then, for each

concept, we generated a meta-analyzed Z-score brain map using NeuroQuery. This toolbox allows users to generate a predictive MRI-derived spatial distribution for any term, based on very large-scale meta-analyses containing mostly functional MRI studies. We excluded 12 concepts containing a term for which NeuroQuery failed to generate a brain map as well as 14 concepts where none of the voxels had a non-zero Z score. As a result, 492 concepts remained.

For each concept-associated brain-map, we calculated the difference in mean χ^2 between voxels statistically significant nominally at 1% level and the rest of voxels in the VBM results. We then computed a pseudo T score for the difference in mean χ^2. These steps were implemented by regressing χ^2 scores on the binary indicator for a voxel having p-value < 0.01. A similar approach has previously been used (*66*)

We then obtained its p-value from 10,000 spatial permutations of the F statistics map from the VBM. We used a generative model approach developed by ref (*55*) for permutation, which allowed us to permute the volumetric brain map with subcortical regions while preserving spatial autocorrelation. For model parameters, we set *ns* = 1,500, *knn* = 800, *pv* = 25, resampling = *True*, which yielded a reasonable fit. We used these permutation-based p-values as a summary measure to evaluate the strength of signal for a given functional concept in relation to SES. We defined statistical significance corrected for multiple testing at the false discovery rate of 5% as we aimed to identify functions with stronger evidence compared to the rest. The full results are reported in Table S17.

5. Interpretation

5.1. Brain, SES, and genetics

To aid interpretation of the association between SES and brain anatomy observed in late adulthood, Fig. S19 describes a simple model that illustrates how adulthood brain anatomy can be linked to SES, family environments, and genetics. The model is depicted in a directed acyclic graph (DAG), a popular graphical framework for identifying confounding variables (*67–69*). The model does not attempt to include all possibly relevant factors and mediating pathways. Rather, its purpose is to identify what effects are potentially captured in the estimated GMV-SES association in relation to genetics and family environments.

It is important to note that each arrow in the DAG represents a unidirectional causal relationship between two variables (nodes). For instance, the arrow from “*SES adult*” to “*Brain adult*” only indicates the environmental effect of adult SES on the adult brain. A path is a set of one or more arrows that connects multiple nodes. A path can be either open or closed. An open path channels statistical associations, which can be closed by conditioning on a variable in the

middle. A path can be closed due to a collider, which is a variable that receives two arrows. Conditioning on a collider opens up a closed path, which induces a collider bias.

Though fairly simple, the model is capable of describing key relevant pathways. First, child brain development is determined by genetics and family environments ("*Own genes → Brain child"* and "*Family environment → Brain child"*). Second, SES in adulthood is a function of genetics, family environments, and child brain development ("*Own genes → SES adult", "Family environment → SES adult",* and *"Brain child → SES adult"*). Third, the transition to the (late) adulthood brain is partly influenced by adult SES ("*Brain child → SES adult → Brain adult"* and *"Brain child → Brain adult"*). Therefore, the model describes the roles of both genetics and family environments in causing differences in SES and the brain. Furthermore, the feedback between SES and the brain is illustrated by the path: "*Brain child → SES adult → Brain adult".* One could extend the model by distinguishing late and early adulthood phases and including another feedback effect. Such an extension, however, will not provide additional key insights as long as socioeconomic mobility is limited during adulthood.

Another important feature is that the model recognizes so-called genetic nurture effects (*38*). Childhood family environments shaped by the parents are known to be associated with the genes of the parents ("*Parental genes → family environment"*), which are passed on to their child ("*Parental genes → Own genes")*. These links induce statistical associations between own genetics and family environments ("*Own genes ← Parental Genes → family environment"*). This fact statistically blurs the common dichotomy between genetics and family environments.

In this study, we regressed voxel-level GMV on an adult SES measure with a goal to estimate the SES-GMV association. If our aim were to estimate the causal effect of adult SES environments on the GMV structure (*i.e.*, "*SES adult → Brain adult"*), a resulting regression estimate will clearly be biased due to the open confounding paths, which transmit statistical associations. Therefore, the estimated SES-GMV associations in this study are expected to encompass the direct environmental effect of adult SES on adult brain and all the effects due to the open paths, which can be summarized as follows:

1) Environmental effects of adult SES on adult brain: *SES adult → Brain adult*
2) Brain causing SES: *SES adult ← Brain child → Brain adult*
3) Genetic effects: *SES adult ← Own genes → Brain child → Brain adult*
4) Family environment effects: *SES adult ← Family environment → Brain child → Brain adult*
5) Genetic nurture effects on brain*: SES adult ← Own genes ← Parental genes → Family environment → Brain child → Brain adult*
6) Genetic nurture effects on SES: *SES adult ← Family environment ← Parental genes → Own genes → Brain child → Brain adult*

Notably, the DAG in Fig. S19 demonstrates that one needs to account for either childhood brain measures (*i.e.*, lifetime longitudinal data) or measures of both family environments and genetics in order to identify the causal effect of the adult SES on the brain (“*SES adult → Brain adult”)*, assuming the absence of no other unobserved confounders.

5.2. Interpretation of the polygenic index for SES

While statistical analysis using PGI_{SES} is straightforward, careful interpretations are required. Most importantly, the remaining associations between the GMV and SES after conditioning on PGI_{SES} cannot entirely be interpreted as environmental effects of SES on the brain anatomy because PGI_{SES} only captures noisy estimates of the effects of measured common genetic variants. It does not include the potential effects of structural or rare genetic variants that are not (or only partly) captured by the observed common genetic variants. Nonetheless, the GMV-SES association that is robust to controlling for PGI_{SES} can point to regions of the brain that are more likely to be affected by environmental factors linked with SES.

To interpret the results, we first need to probe what effects are likely to be summarized in PGI_{SES}. On the basis of the DAG presented in Fig. S19, the GWAS of SES will capture the direct genetic effects on SES (“*Own genes → Brain child → SES adult”* and “*Own genes → SES adult”*) as well as the effects due to confounders, namely genetic nurture effects (“*Own genes ← Parental genes → Family environment → SES adult”* and *“Own genes ← Parental genes → Family environment→ Brain child → SES adult”*). All of these effects will therefore be incorporated in PGI_{SES}. Furthermore, it is important to note that the paths via the adult brain will not be captured in PGI_{SES} due to the adult brain being a collider: “*Own genes → Brain child → Brain adult ← SES adult”.*

These observations lead to the following interpretations for the SES-GMV association estimates conditional on PGI_{SES}. First and most importantly, PGI_{SES} is expected to capture a part of the SES-GMV association due to different family environments and parental SES. A PGI captures the association between a phenotype and genetic variants, rather than causal effects of genetic variants. For this reason, PGI_{SES} will contain genetic nurture effects as described above. Studies have shown that such genetic nurture effects tend to be larger for socio-economic phenotypes (*38*, *70*). Therefore, PGI_{SES} is likely to overstate the genetic effects associated with SES.

Second, what we effectively control for by controlling for PGI_{SES} is the shared genetic architecture between SES and developmental neuroanatomy that is captured by the measured genetic variants and their estimated linear associations with SES. Hence, controlling for PGI_{SES} is not necessarily equivalent to controlling for the entire common genetic variants behind the

GMV-SES association. More specifically, in light of the DAG, PGI_{SES} will account for the following genetic effects on SES: "*Own genes → SES adult*" and *"Own genes → Brain child → SES adult",* the latter of which works via the child brain. On the other hand, PGI_{SES} will not account for the genetic effects on the adult brain that do not work through adult SES: "*Own genes → Brain child → Brain adult*". In fact, in order to account for the underlying genetic effects in the SES-GMV association, it would be required to construct a *PGI* for a brain IDP conditional on adult SES. However, it is currently difficult to construct such a *PGI* with sufficient predictive power due to a limited sample size available for conducting a required GWAS. Moreover, such a *PGI* will need to be constructed for each IDP representing a sufficiently narrow region.

Despite these challenges for interpretation, PGI_{SES} is still useful for identifying brain regions likely to be more susceptible to the influence of socio-economic environments than that of genetic factors. If the estimated SES-GMV association is relatively less attenuated after controlling for PGI_{SES}, the observed SES-GMV association is likely to be a result of environmental effects of SES rather than genetic factors. One reason is because PGI_{SES} tends to overestimate the effects of common genetic variants on SES. Also, at least for healthy individuals, it is highly unlikely that the SES-GMV association is dominantly driven by rare or structural genetic variants with only negligible contribution from common genetic variants associated with SES.

6. Supplementary analyses

6.1. Heritability and genetic correlation

We estimated SNP-based heritability of SES, TIV, and the brainwide GMV scores as well as their pairwise genetic correlation, using genomic-relatedness-based restricted maximum likelihood (GREML) estimation (*71*, *72*). The method estimates the genetic contribution to the phenotypic variance based on a linear mixed model, where the genetic effects are modeled as random. Its extension to a bivariate model estimates genetic correlation between two phenotypes.

We randomly dropped one of a pair of individuals with estimated relatedness greater than 0.05, which resulted in N = 20,447 (*73*). We used a slightly pruned set of the SNPs used to construct PGI_{SES} with the following pruning parameters: window size = 1,000 variant counts, step size = 5, r^2 = 0.95. As a result, 452,190 SNPs were included. As covariates, we included age, age^2, age^3, sex, interaction terms between the sex and age terms, genotyping array indicator, and top 40 genetic PCs. The estimation was implemented in BOLT-REML (*74*).

The results are reported in Table S5. TIV and the GMV score for $PC1_{SES}$ were both partly heritable (h^2 = 0.41, SE = 0.02; and h^2 = 0.28, SE = 0.02, respectively). $PC1_{SES}$ was moderately heritable (h^2 = 0.16, SE = 0.02) and positively genetically correlated with TIV (r_g = 0.37, SE = 0.06).

Furthermore, $PC1_{SES}$ had a moderate genetic correlation with the values of the brainwide GMV score that we constructed for $PC1_{SES}$ (r_g = 0.57, SE = 0.06). Similar estimates for $PC2_{SES}$ and GMV score for $PC2_{SES}$ were either smaller or had much larger standard errors (h^2 = 0.05, SE = 0.02 for $PC2_{SES}$; h^2 = 0.14, SE = 0.02 for GMV score; r_g = 0.18, SE = 0.10 with TIV; r_g = 0.34, SE = 0.18 with the GMV score). Overall, these results demonstrate that the genetic architectures of SES and brain structure are partly overlapping.

6.2. Testing differences in residual SES-GMV associations due to BMI

As presented in Figs. 3C and 3D, the remaining SES-GMV associations after controlling for PGI_{SES} can be substantially attributed to individual differences in BMI. Here we formally tested whether this is the case statistically. In other words, we tested whether there is a statistically significant change in at least one of the coefficients for $PC1_{SES}$ and $PC2_{SES}$ after accounting for BMI in addition to PGI_{SES}. The testing procedure was analogous to the one conducted for PGI_{SES}, which is described in Section 4.3.2, except that GIV regression was used to estimate each model. As it was done for PGI_{SES}, we conducted this test only for the voxels that had significant association with the PCs and then the multiple testing was corrected for using Bonferroni correction. As a result, we found that 84.4% of 34,188 voxels tested had a significant change in at least one of the coefficients for $PC1_{SES}$ and $PC2_{SES}$ after controlling for BMI in addition to PGI_{SES}. This result confirms that BMI can indeed statistically account for the remaining SES-GMV associations after adjusting for PGI_{SES}.

Note that, to measure the contribution of BMI in explaining the remaining SES-GMV associations after controlling for PGI_{SES}, we again used the relative change in the net variation explained by the SES PCs. Hence, following the same logic, we computed the contribution of BMI as:

$$[(R^2_{PC+P\hat{G}I^{(1)}+Z} - R^2_{P\hat{G}I^{(1)}+Z}) - (R^2_{PC+P\hat{G}I^{(1)}+BMI+Z} - R^2_{P\hat{G}I^{(1)}+BMI+Z})] \ / \ (R^2_{PC+P\hat{G}I^{(1)}+Z} - R^2_{P\hat{G}I^{(1)}+Z})$$

6.3. Heterogeneity

Sex and age are two important factors for both SES and neuroanatomy. Therefore, we tested whether the SES-GMV associations are heterogeneous with respect to *i)* different sex (sex interaction) and *ii)* different ages (age interaction). We examined each aspect of heterogeneity separately by using the voxel clusters and including the interaction terms with $PC1_{SES}$ and $PC2_{SES}$. The interaction terms were then tested jointly with F-tests.

The results are reported in Table S23-24. The SES-GMV associations were generally larger for men, with the largest difference found in the biggest cluster from the prefrontal cortex. One exception was found in a small cluster in the cerebellum, where the SES-GMV association was

larger for women. However, none of the regions would survive the brainwide multiple testing correction. The SES-GMV associations also tended to increase with age, while the age interaction estimates were not large enough to be statistically significant even at the uncorrected 5% level, except for one cluster from the anterior insular and the frontal operculum. These null results for age interaction may be due to the survival effect because the majority of the participants were older than 60.

6.4. Controlling for alcohol consumption

Our baseline analyses implicitly adjusted for heavy drinking by excluding heavy drinking individuals. A recent study has shown that even moderate alcohol consumption is associated with reduction in GMV even when educational attainment is adjusted for (*57*). Since alcohol drinking behavior is known to be related to SES, it may be hypothesized that the alcohol consumption is a factor that constitutes the observed SES-GMV associations. However, because individuals with high SES tend to consume a greater amount of alcohol (*75*), controlling for the alcohol consumption is expected to only increase estimates for the SES-GMV associations.

Our data confirms that this is indeed the case. In a cluster-based analysis, we controlled for the alcohol consumption (the number of drinks per week) with linear and square terms. The results show that the SES-GMV associations measured in partial R^2 increased by up to 31%, but only marginally in general (Table S25). Therefore, our positive estimates for the SES-GMV associations cannot be directly attributed to the alcohol consumption. Rather, when not adjusted for, the alcohol intake is a factor that reduces the GMV difference between high and low SES individuals.

6.5. Controlling for cognitive ability and mental health

As with alcohol consumption, there may be several other pathways that may underlie the SES-GMV associations, notably cognitive ability and mental health. Since cognitive ability is positively associated with both SES and GMV, controlling for a cognitive ability measure is expected to decrease the magnitude of a SES-GMV association estimate. Similarly, since mental health status is likely to be negatively associated with both SES and GMV, controlling for a mental health proxy is also expected to decrease the magnitude of a SES-GMV association estimate. Such reduction in the estimate due to controlling for cognitive ability or mental health can be interpreted as the part of SES-GMV association that can be *statistically* attributed to cognitive ability or mental health.

Using GMV clusters with a fluid intelligence score (field 20016) and self-reported mental health proxies (field 2050, 2060, 2070, 2080, 2090, 2100), we examined how much of observed

SES-GMV association can be statistically attributed to cognitive ability or mental health. The testing procedure was equivalent to the analysis described in Section 4.3.2. While this procedure for the fluid intelligence score was straightforward by replacing PGI_{SES} with the fluid intelligence score, some modification was required for mental health.

Since we have multiple proxies of mental health, an auxiliary regression, which corresponds to model (5), was needed for each of six mental health proxies. Accordingly, let each of the parameters δ_1, δ_2, and θ^j now represent a length-6 column vector. The difference in the coefficients for $PC1_{SES}$ and $PC2_{SES}$ can be expressed as: $\beta^j - \tilde{\beta}^j = [\theta^{jT}\delta_1 \;\; \theta^{jT}\delta_2]^T = \Delta^j$, which analogously represents the difference in the SES-GMV association for cluster *j* due to controlling for the mental health proxies. A Wald test was again used to test the null $\Delta^j = 0$ with the test statistic: $\hat{\Delta}^{jT}\, \hat{Var}(\hat{\Delta}^j)^{-1}\hat{\Delta}^j \sim \chi^2_2$, where $\hat{Var}(\hat{\Delta}^j)$ was approximated by the delta method: $\hat{Var}(\hat{\Delta}^j) \approx [\hat{\delta}_1 \;\; \hat{\delta}_2]^T \hat{Var}(\hat{\theta}^j)[\hat{\delta}_1 \;\; \hat{\delta}_2] + [\hat{\theta}^j \;\; \hat{\theta}^j]^T \hat{Var}(\hat{\delta})[\hat{\theta}^j \;\; \hat{\theta}^j]$. $\hat{Var}(\hat{\delta})$ is the covariance matrix for a vector stacking $\hat{\delta}_1$ and $\hat{\delta}_2$. To fully estimate the off-diagonal elements of $\hat{Var}(\hat{\delta})$, we employed a seemingly unrelated regression framework to estimate the auxiliary regression equations.

The results indeed suggest that both cognitive ability and mental health are possible downstream consequences that constitute the relationship between SES and the brain (Fig. S16). Except for a few clusters, controlling for cognitive ability and mental health led to statistically significant differences in the SES-GMV association. While Fluid intelligence accounted for about a half of the overall SES-GMV association for some clusters, mental health accounted for much less on average with 25.2% at the maximum.

To conclude, we emphasize that the purpose of these control analyses was *not* to show that factors such as cognitive ability and mental health are *confounders* to SES-GMV *association*. Our aim was to robustly identify the associations between the brain structure and SES, not the causal effect of SES on the brain structure or vice versa. Since our target was an associational quantity, our approach does not necessarily require isolating potential mediators or potential downstream consequences. Instead, our main analyses only controlled for the upstreatm sources of variation in SES and the brain structure, which included differences due to sex, age, and genetic population structure. Factors such as cognitive ability and mental health are co-variations of key interest, which could constitute the link between SES and the brain rather than confound it (see Section 3.3). Controlling for cognitive abilities and mental health could lead to understating the magnitude of the relationship between SES and the brain as well as the relevant role of genetics. Therefore, we did not control for such factors in our main analysis.

6.6. Heterogeneity between genetic ancestry groups

In the first set of our analyses, where we aimed to identify robust SES-GMV associations, we included samples with multiple genetic ancestry groups. Therefore, it may be of interest to explore heterogeneity between genetic ancestry groups. Because the majority of the UKB sample is those of European ancestry, we conducted a stratified analysis on samples of European (EUR) ancestry and Non-European (Non-EUR) ancestry, by using GMV clusters. The results suggest some degree of heterogeneity between EUR and non-EUR samples (Fig. S17). However, the estimates for the non-EUR sample were often too noisy to be informative. Although the results do not find statistically significant differences in the results for the European and non-European ancestry groups, we believe that some degree of heterogeneity between the two samples could exist due to the fact that the non-European sample is from social and ethical minority groups in the UK, which implies that the two samples represent different underlying population samples.

As an additional robustness check, we also conducted a meta-analysis of the EUR and non-EUR specific results. We meta-analyzed the stratified analysis results with inverse-variance weights. Fig. S18 shows that the meta-analyzed results are essentially identical to the original results from the pooled analysis. These results also match statistical expectations. By the law of total covariance, it can be analytically shown that the OLS estimator with the pooled sample produces a weighted average of the estimates from each stratified group as well as the estimate reflecting the between-group differences. Because the between-ancestry difference in SES and the brain phenotype can be accounted for by the genetic PCs, our results already represented a weighted average of the European and the Non-European ancestry subsamples as shown in Fig. S17. The weights from this pooled analysis are almost identical to the inverse-variance weights used in the meta-analysis because the OLS estimator also exploits inverse-variance weights to minimize the variance. As a result, the meta-analysis produced barely different results.

6.7. The second principal component of SES

We pre-registered to use the two PCs because the first two PCs are both necessary and sufficient to explain the overall SES variation in the UKB sample as demonstrated in Figs. 1B and S2. Whenever possible, we refrained from reporting results for each PC because our statistical quantity of main interest, as pre-registered, is the joint association of the two PCs with brain structure phenotypes. For this reason, we measured SES-GMV association with partial R^2 throughout the paper. While it was not our intention to examine each PC separately, we briefly reported results for each PC as by-products for some analyses.

As shown in Figs. 2 and S4A, $PC2_{SES}$ turned out to have much weaker associations with the brain than does $PC1_{SES}$. Nonetheless, we deemed $PC2_{SES}$ to be relevant *ex-post* for two reasons: First, it gave us more power to identify brain regions significantly associated with SES. Second, $PC2_{SES}$ contributed to capturing non-genetic variation in SES.

Despite its weaker associations with GMV, $PC2_{SES}$ still captures the difference in GMV above and beyond what $PC1_{SES}$ does, which is also statistically detectable for some voxels. As a result, examining the joint association of the two PCs allowed us to find 11% more voxels significantly associated with SES compared to when only $PC1_{SES}$ was used.

More importantly, $PC2_{SES}$ turned out to be barely heritable as reported in Section 6.1. If we excluded $PC2_{SES}$ in the analysis, we would have only kept the part of SES-GMV association that is more heritable; consequently, we would have overstated the role of genetics than what the data actually suggests. The regions that have relatively larger association with $PC2_{SES}$, such as lateral temporal and cerebellar regions, tend to overlap with the regions that we found to be susceptible to the influence of the socioeconomic environment. Therefore, $PC2_{SES}$ played a crucial role in differentiating genetic and environmental influences to the SES-GMV relation.

7. Supplementary discussion

Our results quantify the extent to which SES could be an underlying cause or a confounding factor in brain imaging studies concerned with cognitive health or behavior. Furthermore, the modest effect sizes we found for specific brain regions imply that large sample sizes are required to identify robust neuroanatomical associations with SES. For example, a sample size of $N > 1{,}200$ or $N > 2{,}800$ is required for obtaining 90% statistical power to detect even the largest effect we found (partial $R^2 = 1.3\%$) at p-values of 0.01 or 2×10^{-6}, respectively. The effect sizes reported in this paper are far smaller than typically reported in smaller-scale studies in the past. Due to their small sample size, some of the past reports may be inflated estimates that suffer the winner's curse (*11*).

Importantly, small effect sizes for individual voxels do not necessarily imply that the association between SES and brainwide GMV structure is also negligible. The brainwide GMV score we constructed was able to predict almost 5% of the *out-of-sample* variation of SES. The predictive accuracy of the score can be further improved with a larger training sample. Furthermore, this result suggests that the overall association between SES and GMV will be far from being modest if it is estimated in-sample.

Of note, the modest effect sizes can partly be due to the fact that the UKB participants generally hold a higher SES than the general British population (*76*). As Fig S6 suggests, SES-GMV associations are likely to be larger for lower SES individuals and therefore our results

may underestimate the true SES-GMV association size for the general population. Furthermore, in order to identify robust SES-GMV association, we excluded individuals who were clinically diagnosed with a brain disease, morbidly obese, and heavy-drinking. Such sample exclusion criteria can attenuate the estimates since these traits are known or likely to be negatively associated with both SES and GMV. Fig S12 demonstrates that this was indeed the case.

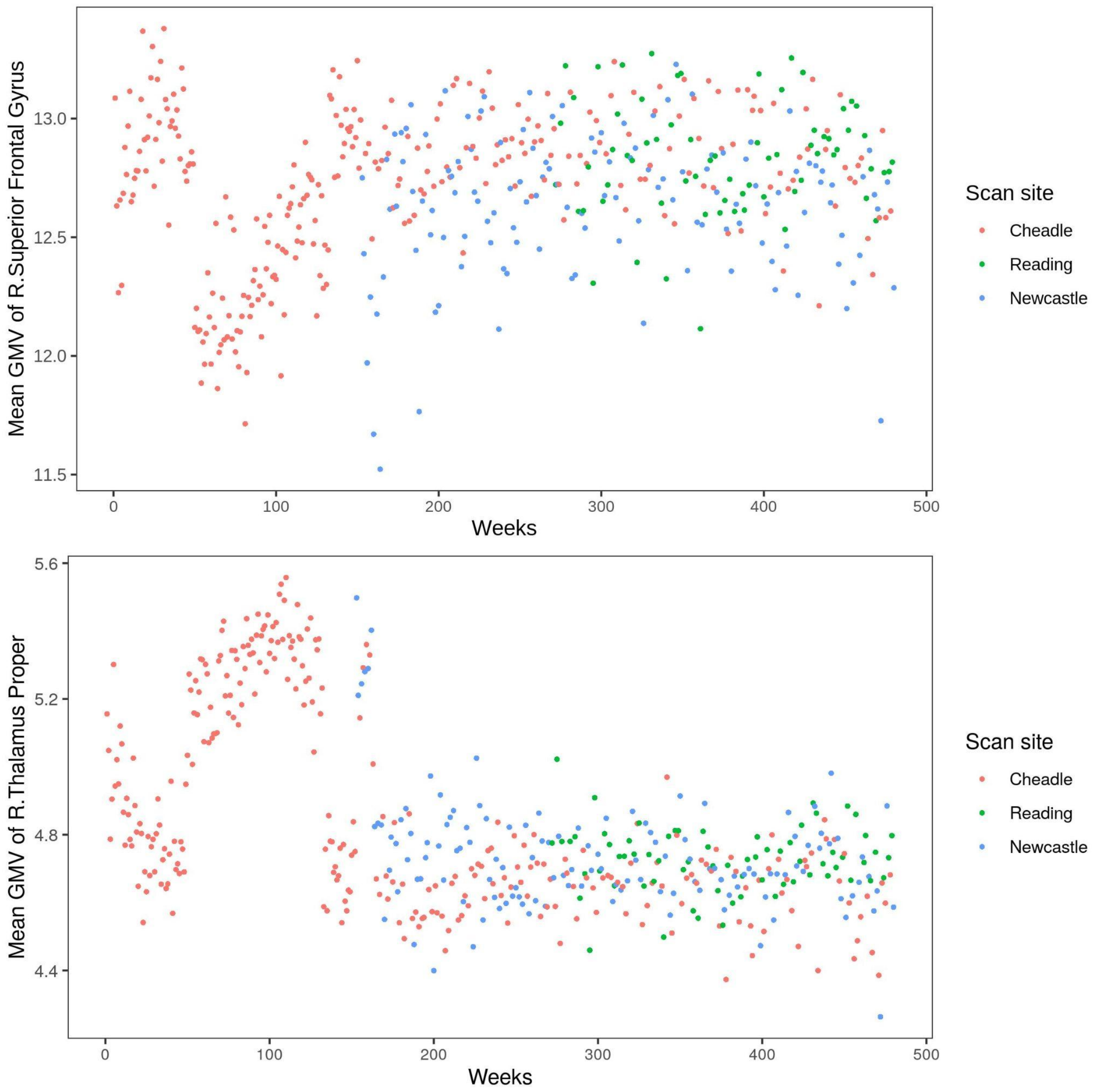

Figure S1. Average grey matter volume over acquisition dates by week

Site-specific weekly averages of grey matter volume are plotted over acquisition dates for the right superior frontal gyrus and the right thalamus. These regions are selected as examples. Other regions show similar patterns.

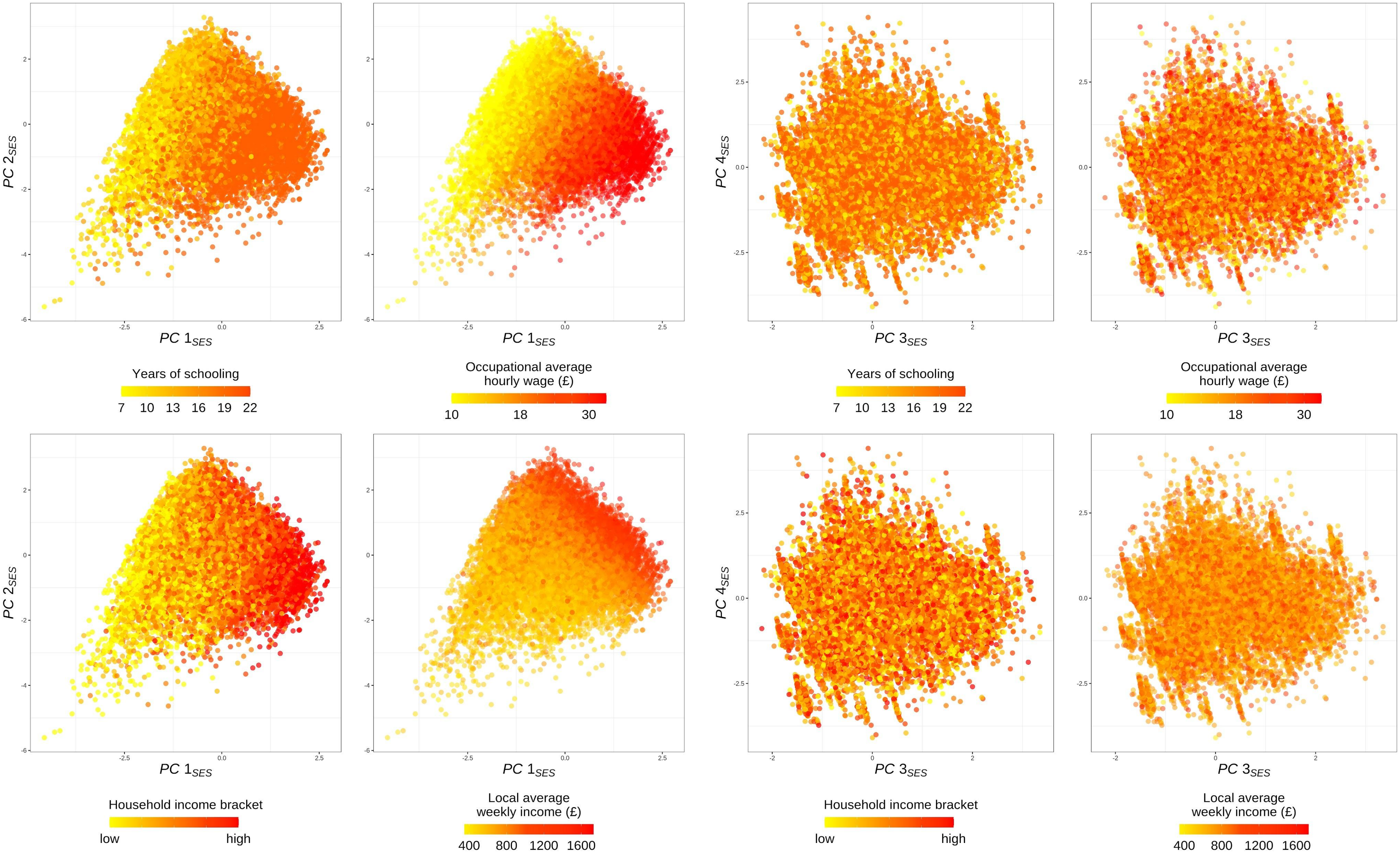

Figure S2. Scatter plots of top principal components (PC) for socioeconomic status (SES)

Top four PCs for SES are plotted with color indicating different levels of selected socioeconomic measures.

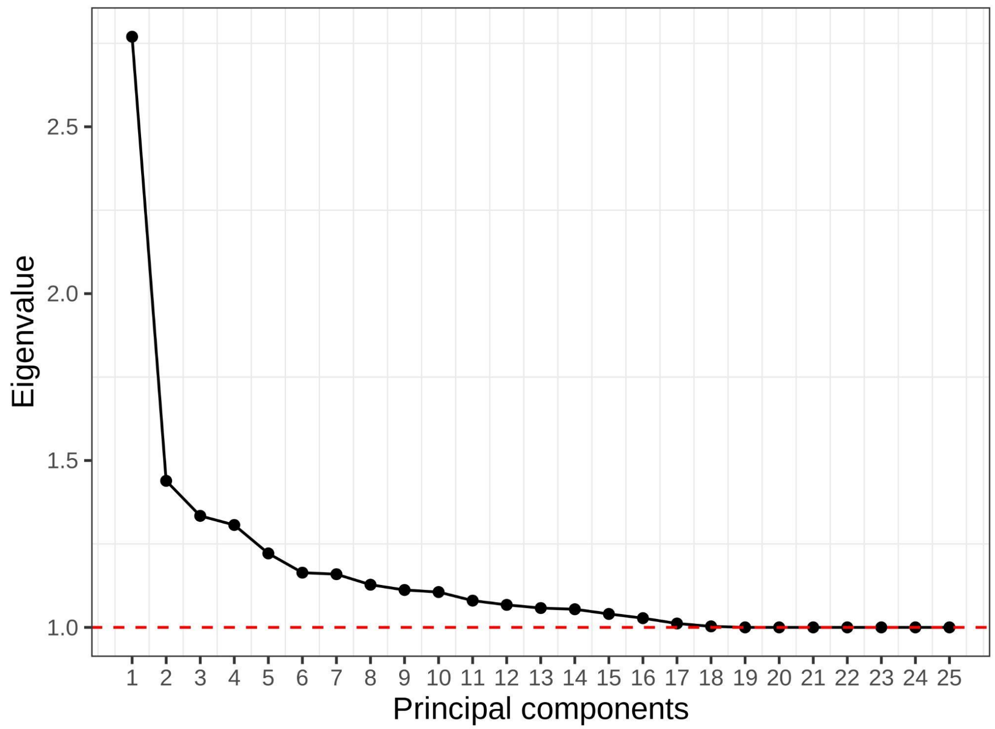

Figure S3. Eigenvalues from the principal component analysis of socioeconomic indicators

A.

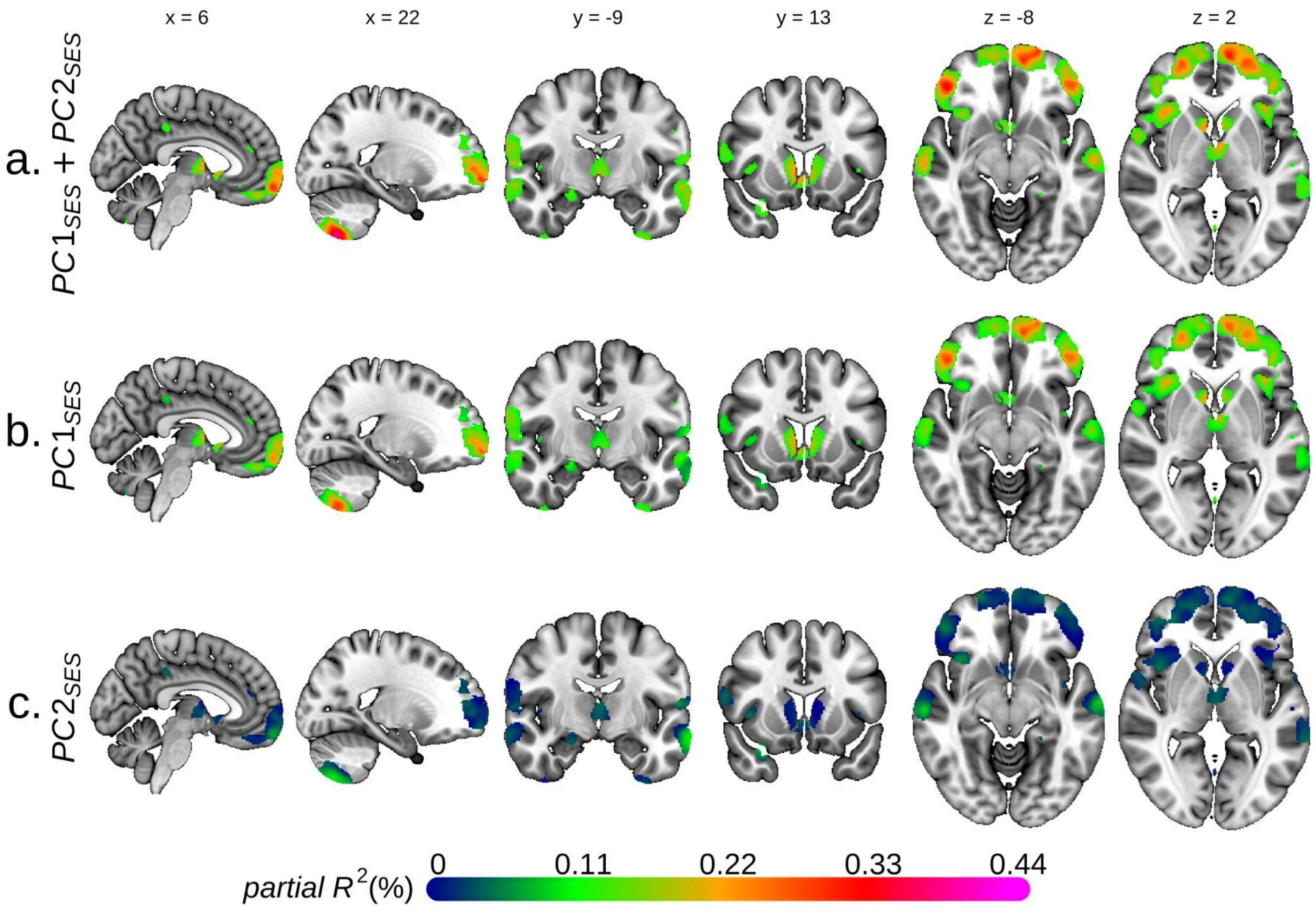

x = 6
x = 22
y = -9
y = 13
z = -8
z = 2
a. $PC1_{SES} + PC2_{SES}$
b. $PC1_{SES}$
c. $PC2_{SES}$
partial R^2(%)
0
0.11
0.22
0.33
0.44

B.

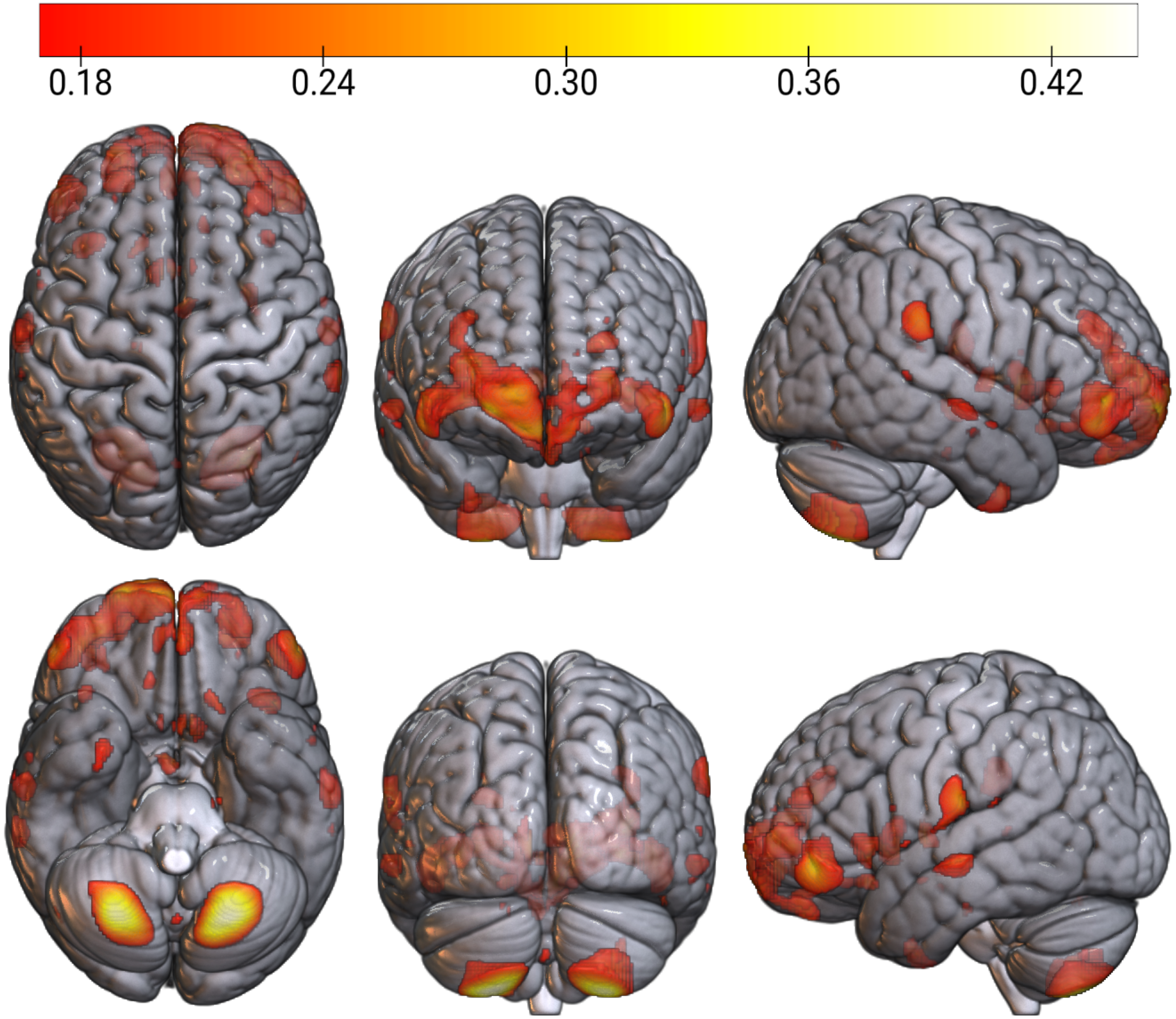

Figure S4. Voxel-based morphometry of grey matter volume and socioeconomic status (SES)

Univariate voxel-based morphometry results on the two principal components (PC) for SES.

A. Each figure plots the association sizes measured in partial R^2 for only the voxels significant at FWE rate of 5%. MNI coordinates are indicated.

B. Partial R^2 (%) for two SES PC is plotted for only the voxels that were significant at FWE rate of 5% and had partial R^2>0.17%.

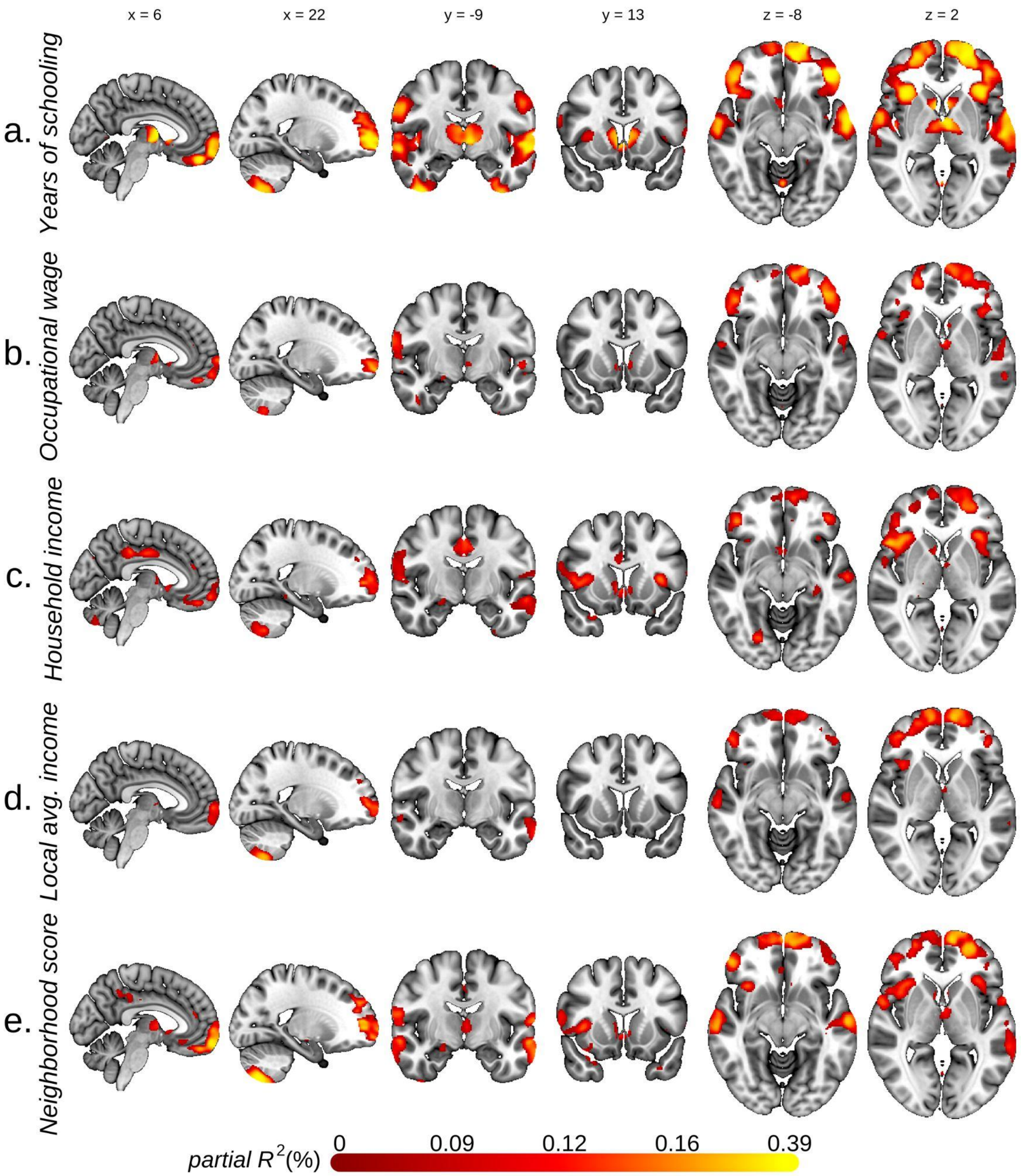

Figure S5. Voxel-based morphometry of grey matter volume and various socioeconomic measures

Univariate voxel-based morphometry results, with grey matter volume as the dependent variable and each of the five socioeconomic measures as the explanatory variable. Each figure plots the association sizes measured in partial R^2 for only the voxels significant at FWE rate of 5%. MNI coordinates are indicated. N = 30,954 (a), 27,307 (b), 29,839 (c), 29,006 (d), 28,405 (e)

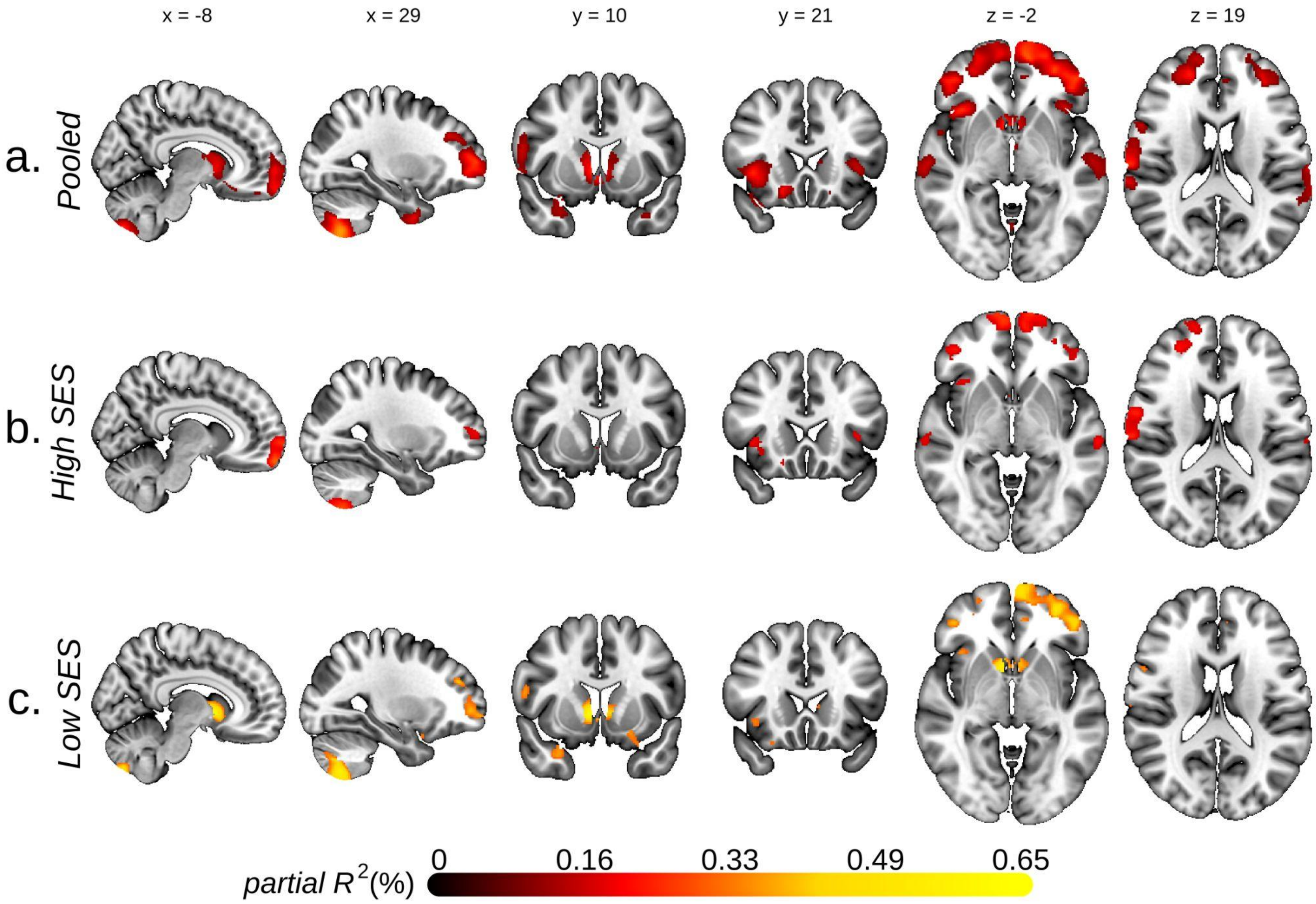

Figure S6. Stratified analyses on low and high socioeconomic status groups

Results from baseline voxel-based morphometry analysis conducted separately on low and high socioeconomic status (SES) groups as well as the pooled sample. Each figure plots the association sizes measured in partial R^2 for only the voxels significant at FWE rate of 5%. High and low SES groups were defined by National Statistics Socio-economic Classification (high if holding a managerial, administrative, or professional occupation). MNI coordinates are indicated. N = 23,931 (a), 15,611 (b), 8,320 (c).

A.

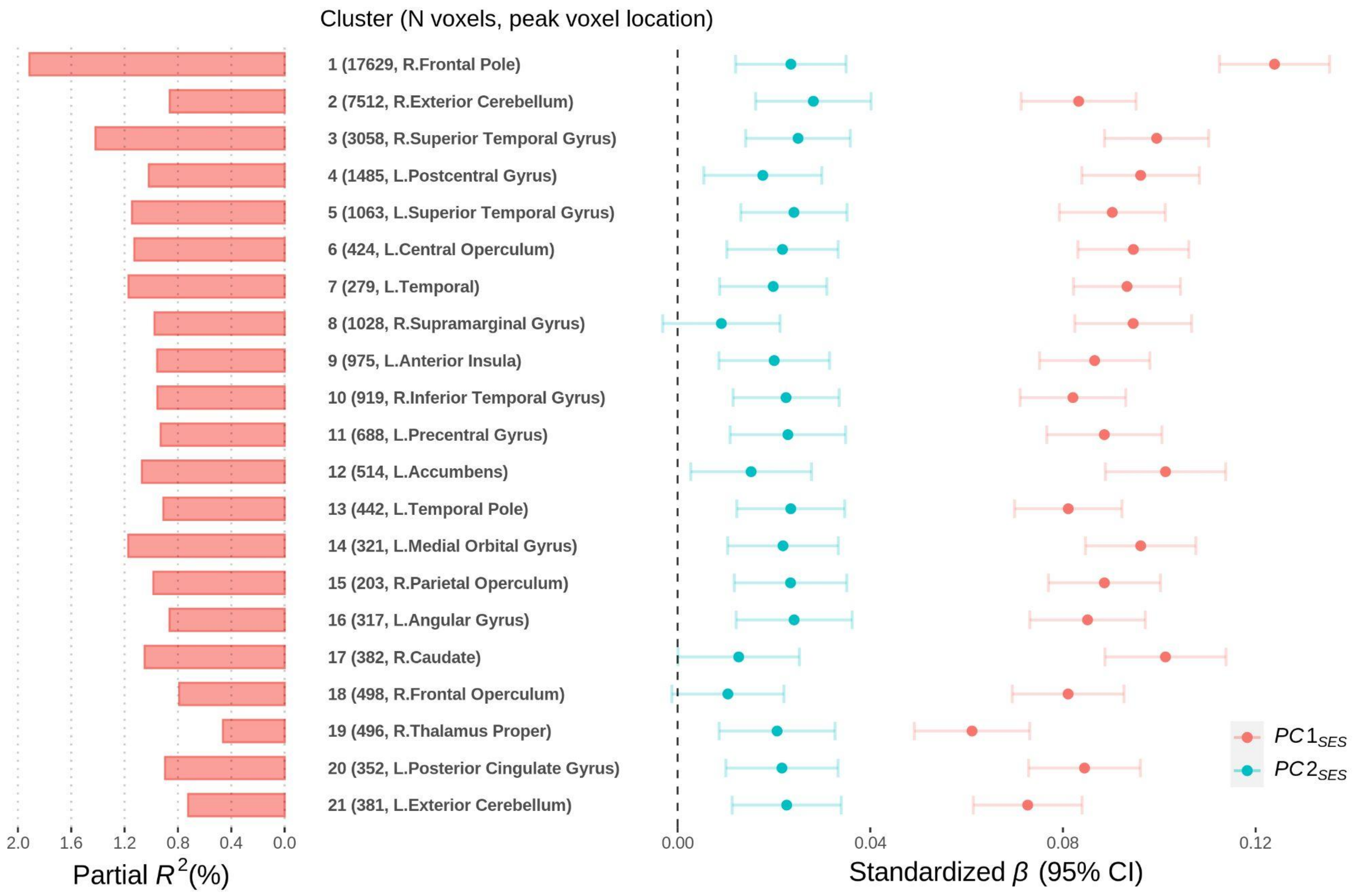
Cluster (N voxels, peak voxel location)
1 (17629, R.Frontal Pole)
2 (7512, R.Exterior Cerebellum)
3 (3058, R.Superior Temporal Gyrus)
4 (1485, L.Postcentral Gyrus)
5 (1063, L.Superior Temporal Gyrus)
6 (424, L.Central Operculum)
7 (279, L.Temporal)
8 (1028, R.Supramarginal Gyrus)
9 (975, L.Anterior Insula)
10 (919, R.Inferior Temporal Gyrus)
11 (688, L.Precentral Gyrus)
12 (514, L.Accumbens)
13 (442, L.Temporal Pole)
14 (321, L.Medial Orbital Gyrus)
15 (203, R.Parietal Operculum)
16 (317, L.Angular Gyrus)
17 (382, R.Caudate)
18 (498, R.Frontal Operculum)
19 (496, R.Thalamus Proper)
20 (352, L.Posterior Cingulate Gyrus)
21 (381, L.Exterior Cerebellum)
2.0 1.6 1.2 0.8 0.4 0.0
Partial R^2(%)
0.00 0.04 0.08 0.12
Standardized β (95% CI)
$PC1_{SES}$
$PC2_{SES}$

B.

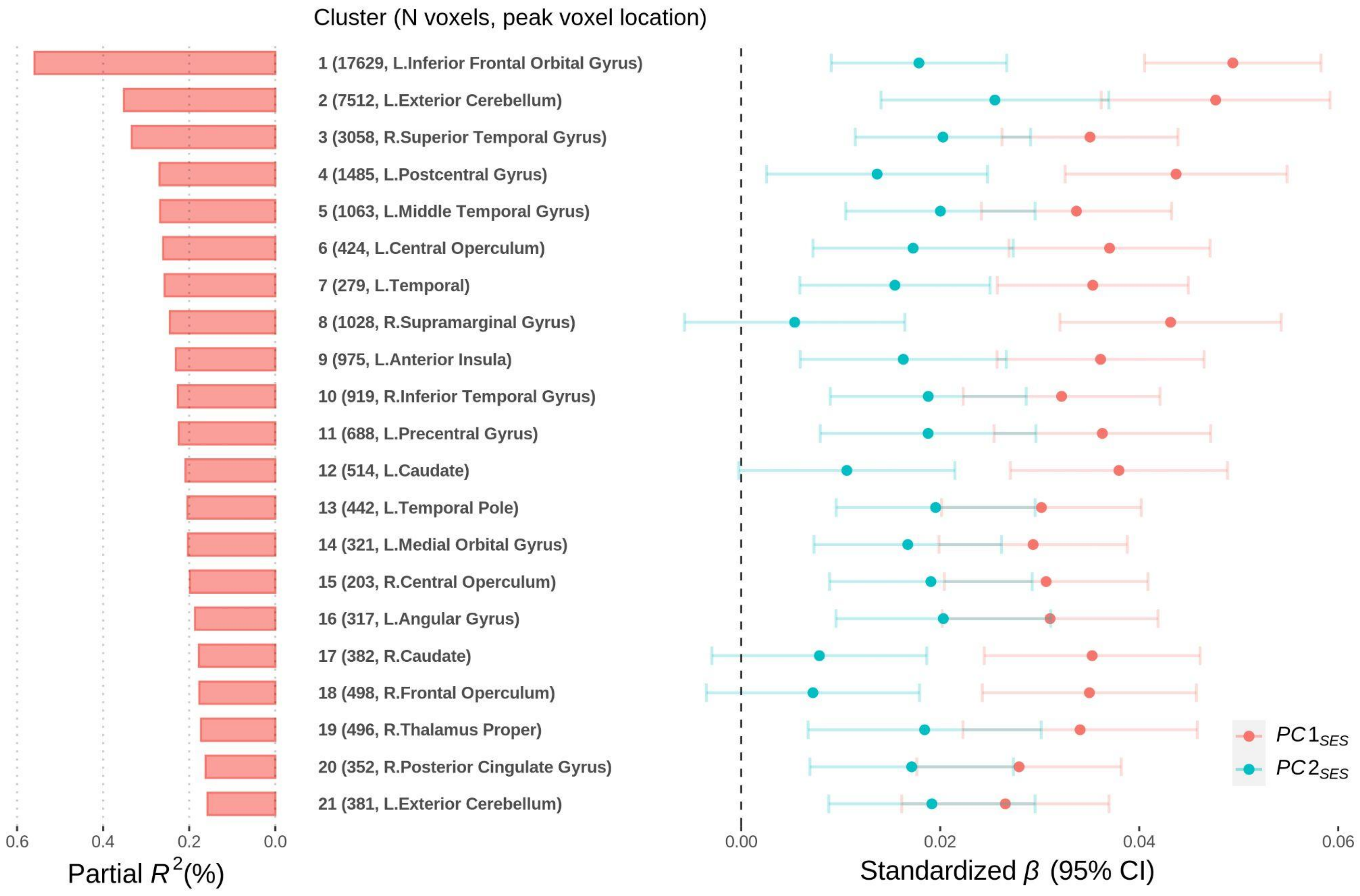

Figure S7. Standardized effect sizes of associations between socioeconomic status (SES) and grey matter volume (GMV) in voxel clusters

Results from regressing GMV in each cluster on $PC1_{SES}$ and $PC2_{SES}$. Total intracranial volume was not controlled for in **A.** but in **B.** On the left, partial R^2 from both $PC1_{SES}$ and $PC2_{SES}$ are reported and, on the right, the standardized coefficient estimates are plotted with their uncorrected 95% confidence intervals. The clusters were formed with at least 200 voxels showing significant associations at FWE rate of 5% level in the baseline TIV-adjustd voxel-based morphometry (VBM) results on $PC1_{SES}$ and $PC2_{SES}$. For each cluster, the anatomical location of the peak voxel from the VBM results is indicated. See Table S8 for more information about the clusters.

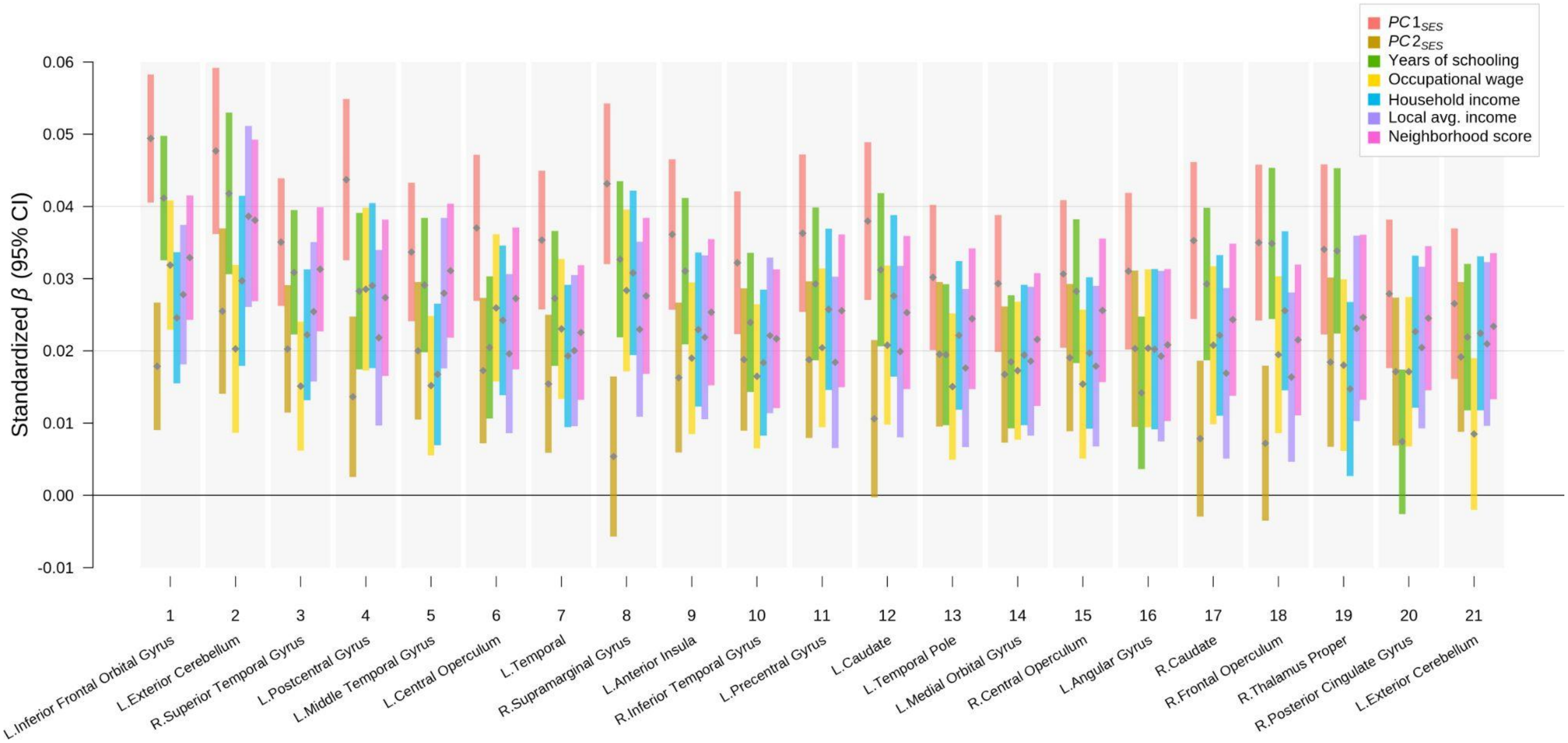

Figure S8. Standardized effect sizes of associations between various socioeconomic measures and grey matter volume (GMV) in voxel clusters

Results from regressing GMV in each cluster on each of the socioeconomic measures separately. The standardized coefficient estimates (grey points) are plotted with their uncorrected 95% confidence intervals (color bars). The clusters were formed with at least 200 voxels showing significant associations at FWE rate of 5% level in the baseline voxel-based morphometry (VBM) results on $PC1_{\mathrm{SES}}$ and $PC2_{\mathrm{SES}}$. The clusters are ordered by the strength of joint associations with $PC1_{\mathrm{SES}}$ and $PC2_{\mathrm{SES}}$. For each cluster, the anatomical location of the peak voxel from the VBM results is indicated at the bottom. See Table S8 for more information about the clusters.

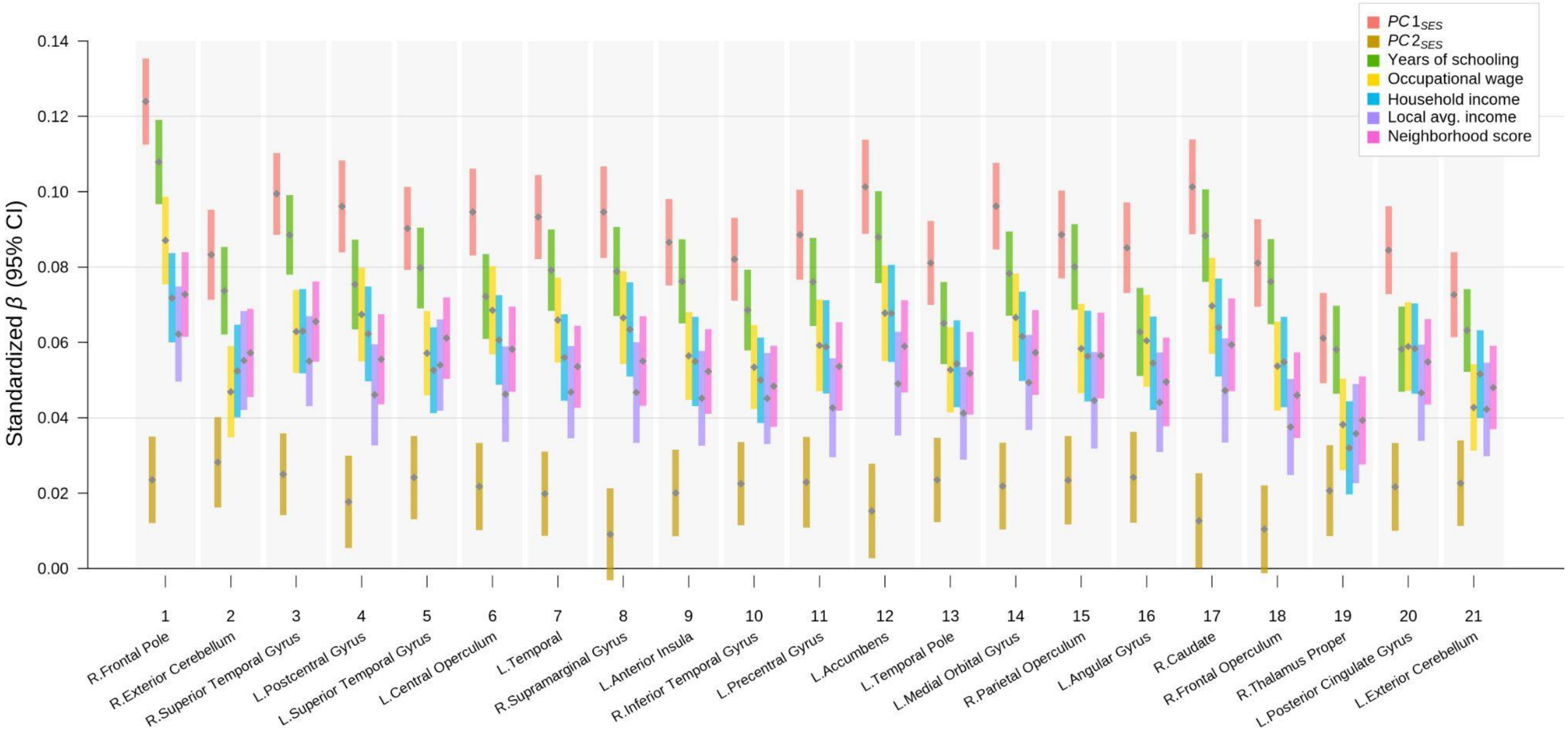

Figure S9. Standardized effect sizes of TIV-unadjusted associations between various socioeconomic measures and grey matter volume (GMV) in voxel clusters

Results from regressing GMV in each cluster on each of the socioeconomic measures separately while not controlling for total intracranial volume (TIV). The standardized coefficient estimates (grey points) are plotted with their uncorrected 95% confidence intervals (color bars). The clusters were formed with at least 200 voxels showing significant associations at FWE rate of 5% level in the baseline voxel-based morphometry (VBM) results on $PC1_{SES}$ and $PC2_{SES}$. The clusters are ordered by the strength of joint associations with $PC1_{SES}$ and $PC2_{SES}$. For each cluster, the anatomical location of the peak voxel from the VBM results is indicated at the bottom. See Table S8 for more information about the clusters.

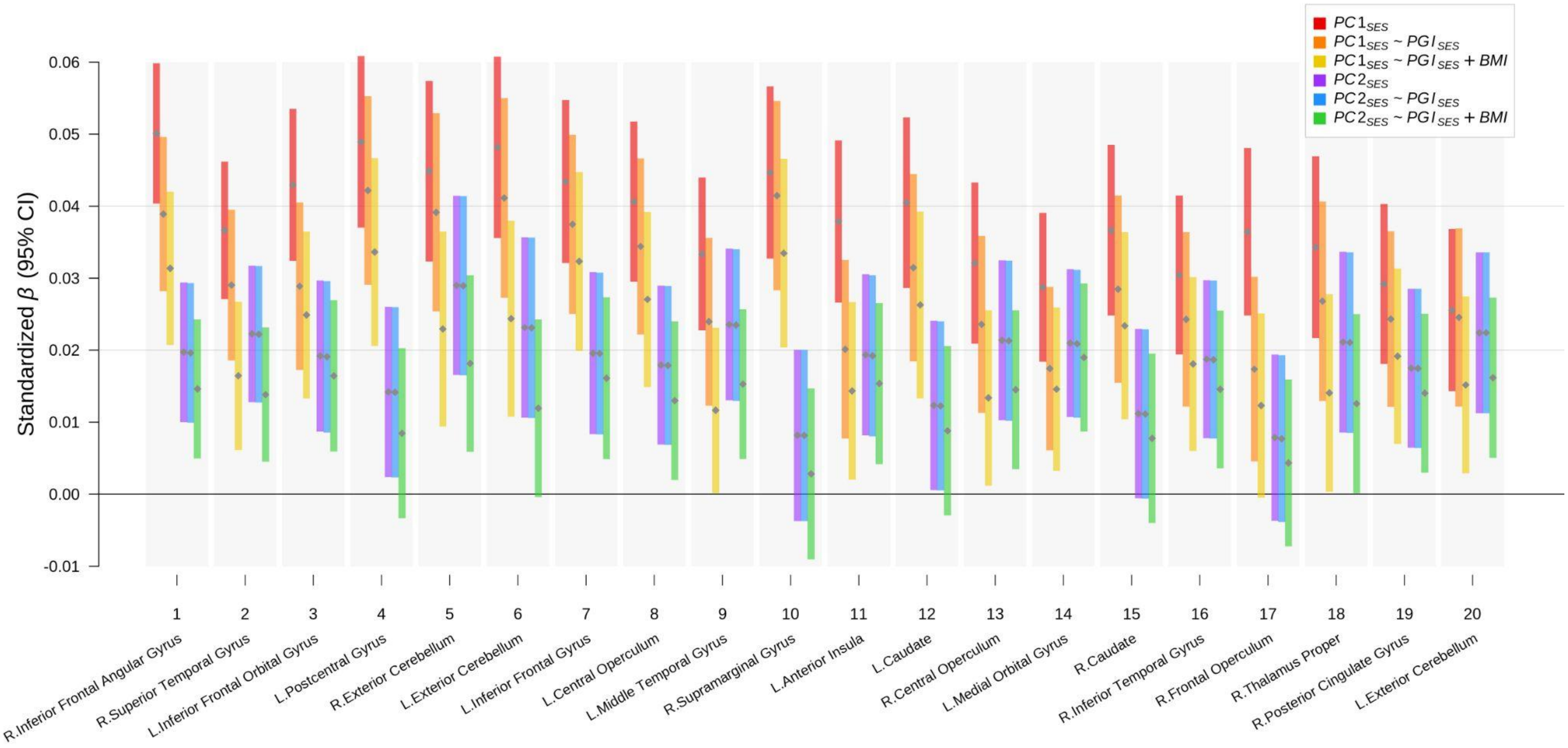

Figure S10. Standardized effect sizes of associations between socioeconomic status (SES) and grey matter volume (GMV) in voxel clusters with additional controls

Results from regressing GMV in each cluster on $PC1_{\text{SES}}$ and $PC2_{\text{SES}}$ while additionally controlling for PGI_{SES} and BMI. The standardized coefficient estimates (grey points) are plotted with their uncorrected 95% confidence intervals (color bars). The sample was restricted to individuals of European ancestry. Measurement error in PGI_{SES} is adjusted for with genetic instrument variable (GIV) regression. The clusters were formed with at least 200 voxels showing significant associations at FWE rate of 5% level in the baseline voxel-based morphometry (VBM) results on $PC1_{\text{SES}}$ and $PC2_{\text{SES}}$. The clusters are ordered by the strength of joint associations with $PC1_{\text{SES}}$ and $PC2_{\text{SES}}$. For each cluster, the anatomical location of the peak voxel from the VBM results is indicated at the bottom. See Table S9 for more information about the clusters.

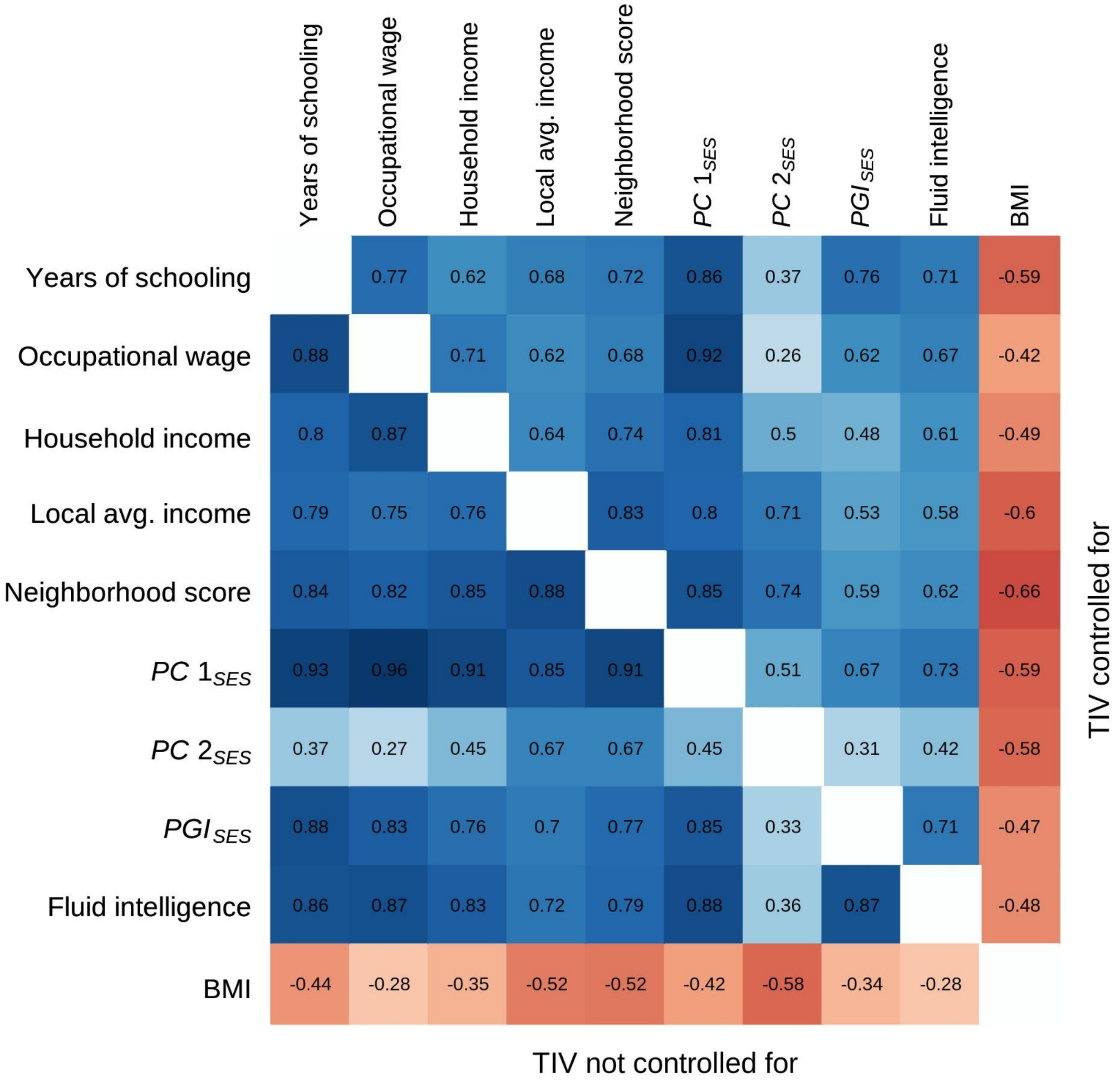

Figure S11. Grey-matter neuroanatomical correlations of various measures

The figure plots pairwise Pearson correlations computed from *T*-statistics of univariate grey-matter VBM results for each measure. The upper triangle reports the correlations of the *T*-statistics from the VBM analyses that adjusted for total intracranial volume (TIV), while the lower triangle from those that did not adjust for TIV.

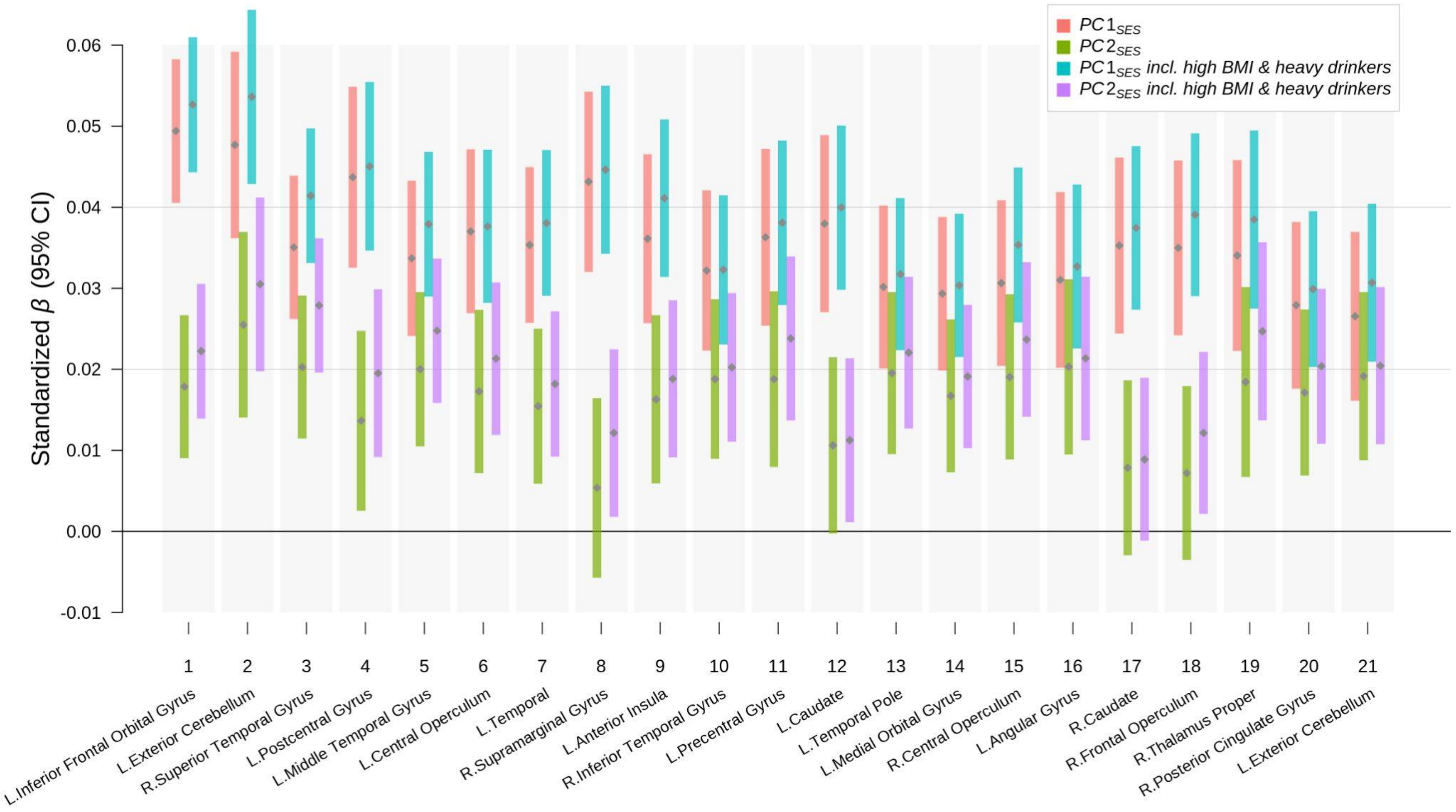

Figure S12. Standardized effect sizes of associations between socioeconomic status and grey matter volume (GMV) in voxel clusters with and without morbidly obese and heavy drinking individuals

Results from regressing GMV in each cluster on $PC1_{SES}$ and $PC2_{SES}$ with and without morbidly obese and heavy drinking individuals. The standardized coefficient estimates (grey points) are plotted with their uncorrected 95% confidence intervals (color bars). The clusters were formed with at least 200 voxels showing significant associations at FWE rate of 5%

level in the baseline voxel-based morphometry (VBM) results on $PC1_{\mathrm{SES}}$ and $PC2_{\mathrm{SES}}$. The clusters are ordered by the strength of joint associations with $PC1_{\mathrm{SES}}$ and $PC2_{\mathrm{SES}}$. For each cluster, the anatomical location of the peak voxel from the VBM results is indicated at the bottom. See Table S8 for more information about the clusters.

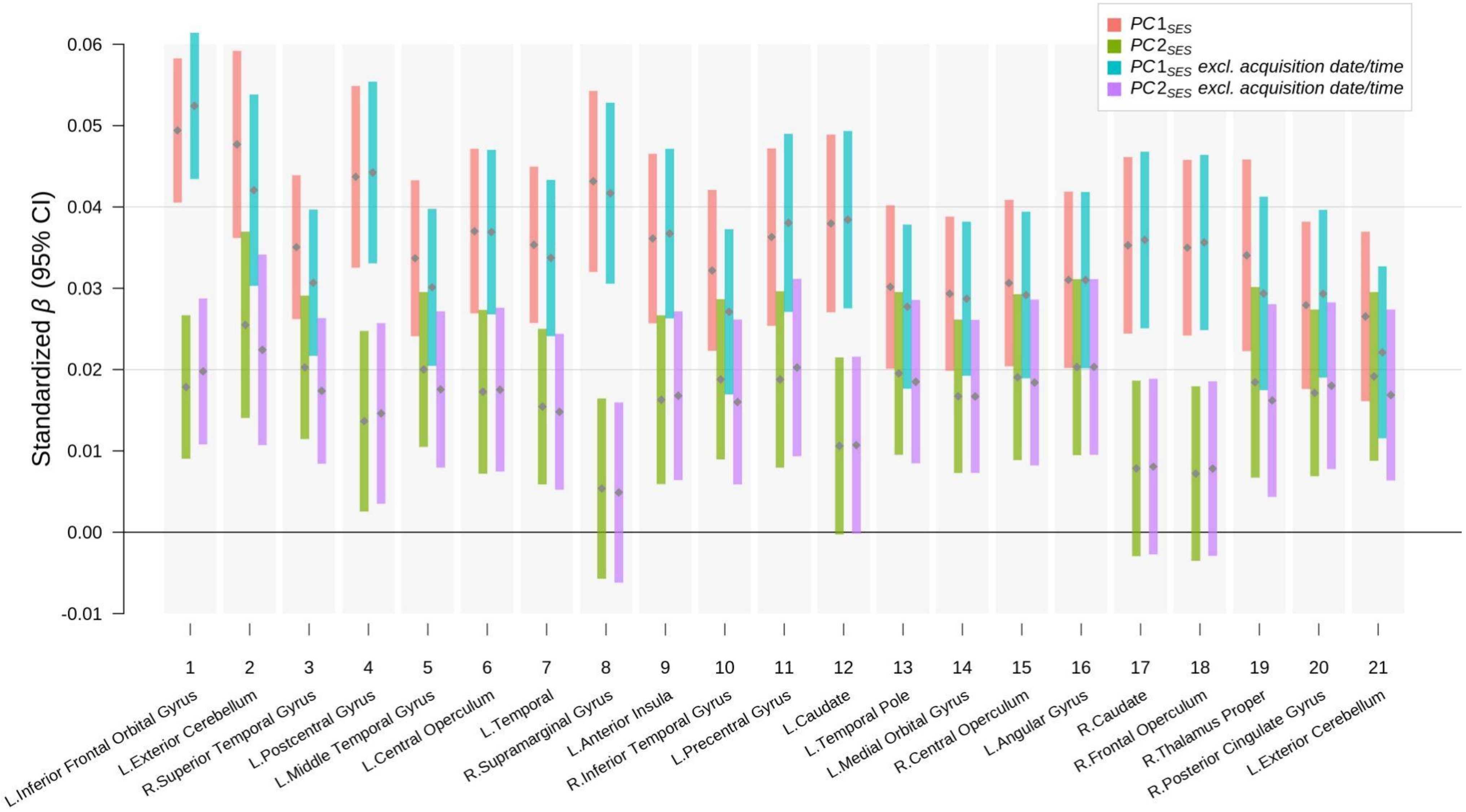

Figure S13. Standardized effect sizes of associations between socioeconomic status and grey matter volume (GMV) in voxel clusters with and without acquisition date and time as control variables

Results from regressing GMV in each cluster on $PC1_{SES}$ and $PC2_{SES}$ with and without the acquisition date and time as control variables. The standardized coefficient estimates (grey points) are plotted with their uncorrected 95% confidence intervals (color bars). The clusters were formed with at least 200 voxels showing significant associations at FWE rate of 5%

level in the baseline voxel-based morphometry (VBM) results on $PC1_{SES}$ and $PC2_{SES}$. The clusters are ordered by the strength of joint associations with $PC1_{SES}$ and $PC2_{SES}$. For each cluster, the anatomical location of the peak voxel from the VBM results is indicated at the bottom. See Table S8 for more information about the clusters.

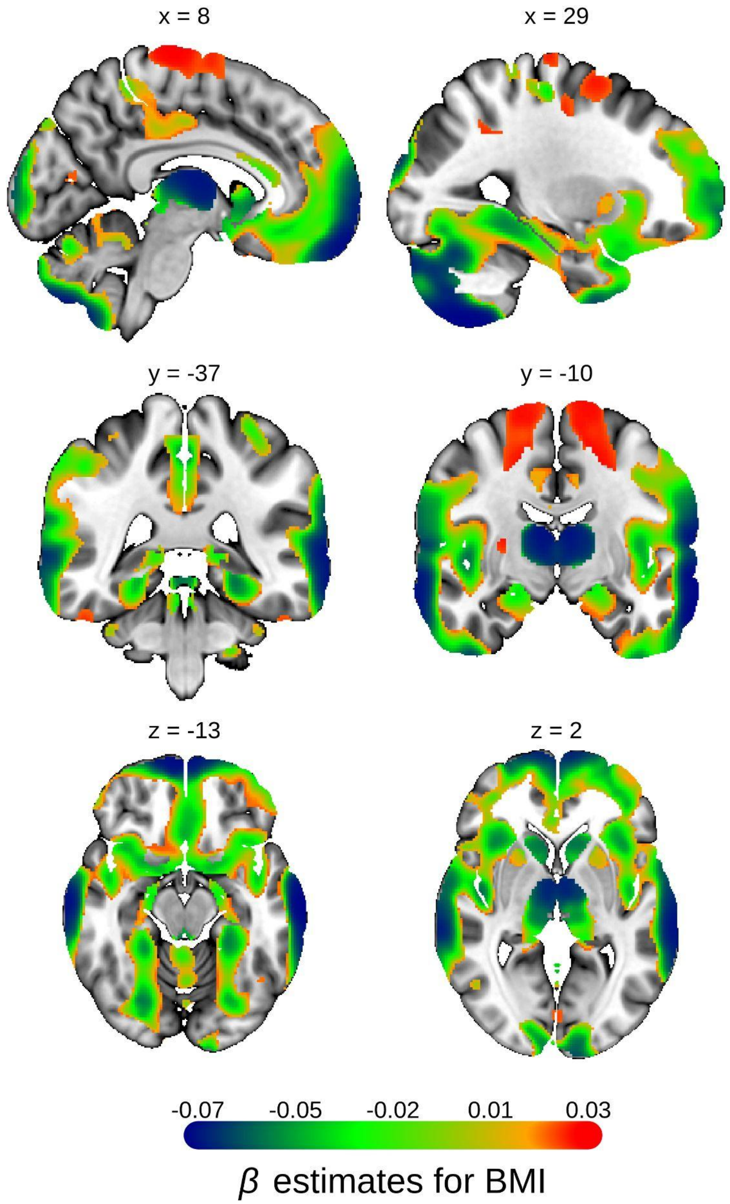

Figure S14. Voxel-based morphometry (VBM) of body mass index (BMI)

Univariate VBM results on BMI, with grey matter volume (GMV) as the dependent variable. The Beta estimates are plotted for voxels significant at FWE rate of 1% level with partial R^2>0.02%. The Beta estimates are reported in the standard deviation unit of GMV. MNI coordinates are indicated.

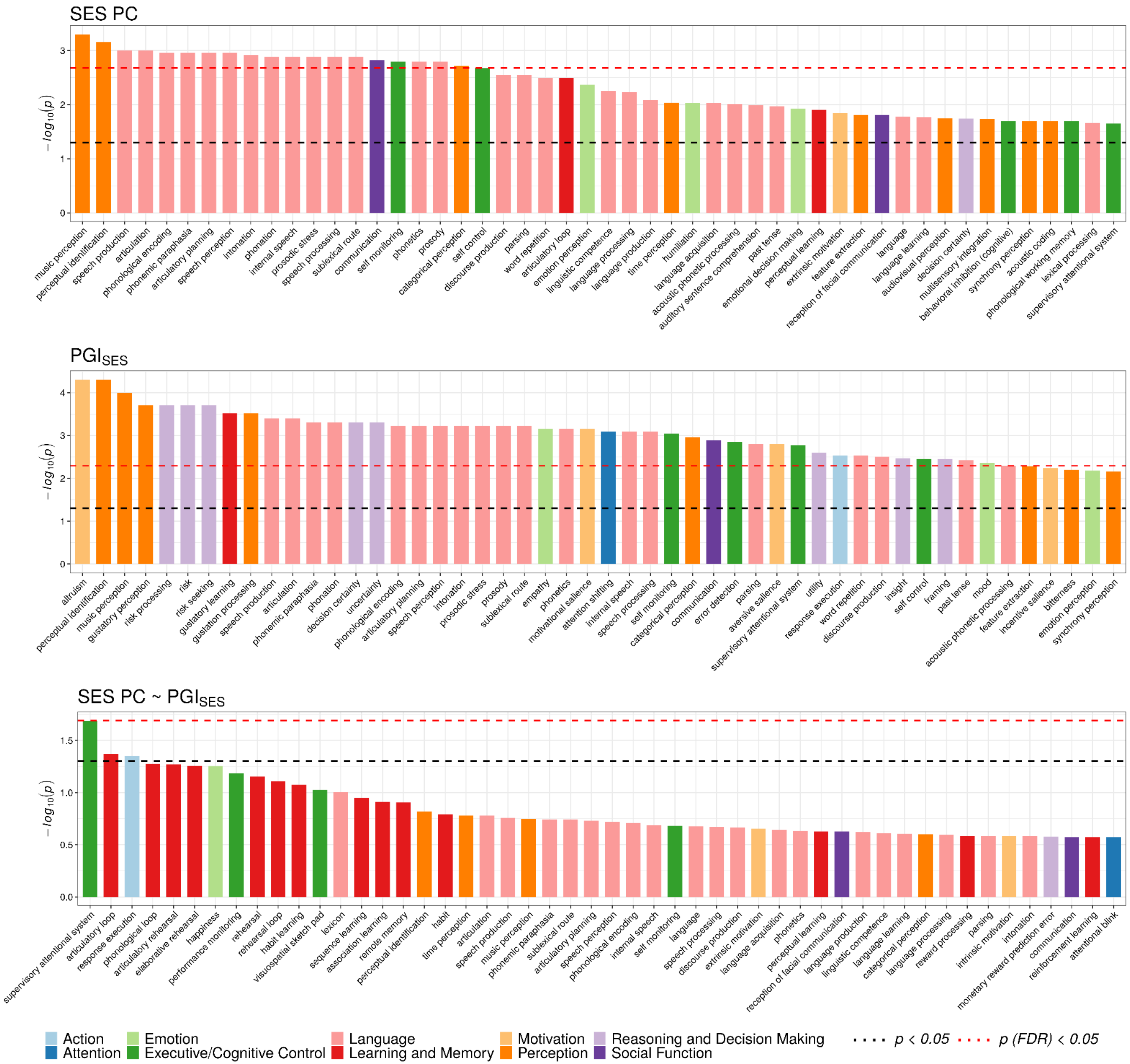

Figure S15. Bar plots of functional annotation of brain regions associated with socioeconomic status (SES)

492 cognitive concepts, belonging to 10 categories, were taken from Cognitive Atlas and their predicted fMRI meta-analysis results were generated by NeuroQuery. For each concept, we computed the difference in mean χ^2 between voxels statistically significant at 1% level and the rest of voxels. Then, a pseudo T score for the difference was computed and its p-value

was obtained by 10,000 spatial permutations. This procedure was carried out for the voxel-based morphometry results respectively for **1)** two principal components (PC) of SES, **2)** the polygenic index for SES (PGI_{SES}), and **3)** SES controlling for PGI_{SES}. The *p*-values of the top 50 concepts were plotted on log_{10} scale for each result.

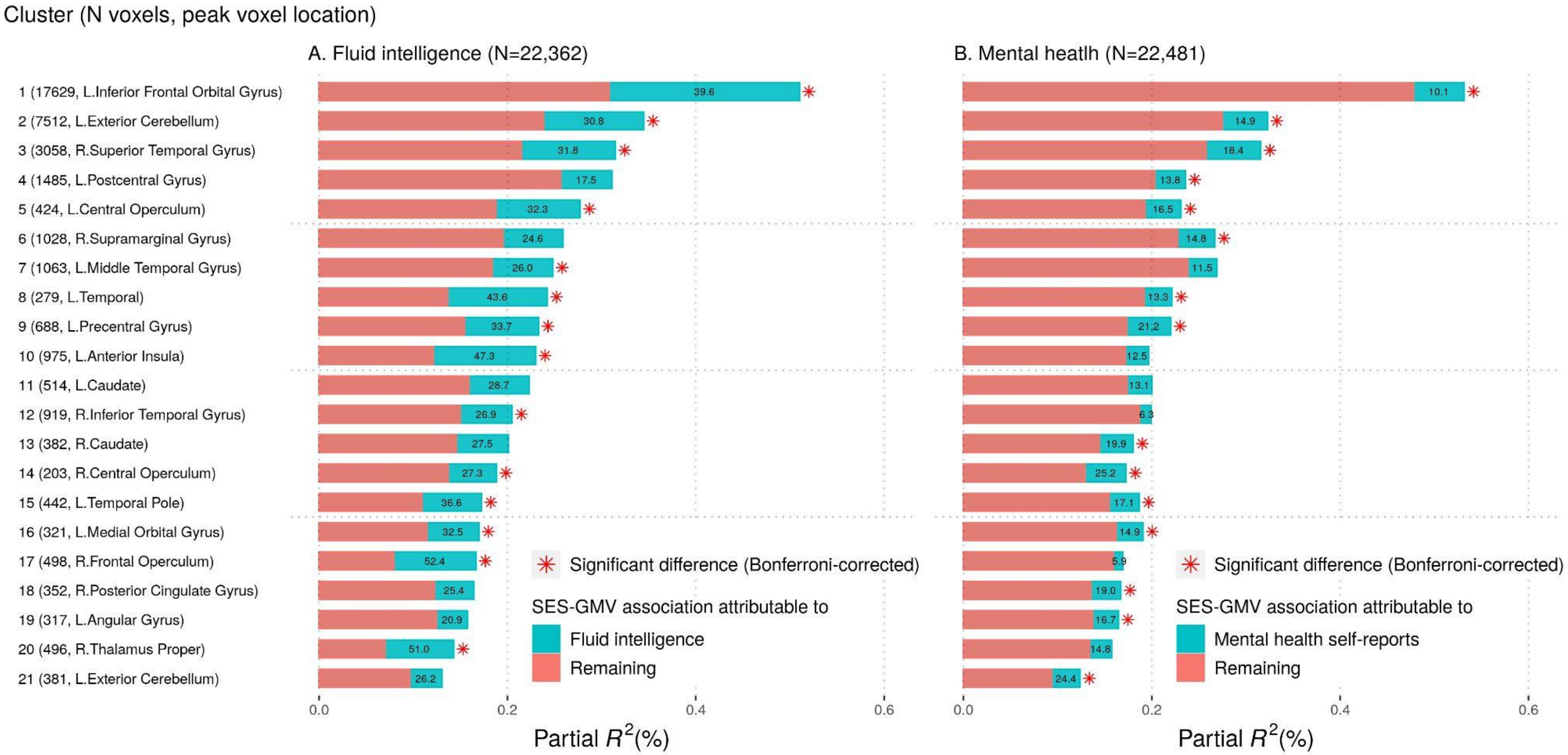

Figure S16. Control analysis with fluid intelligence score and mental health proxies

Associations in partial R^2 between the two PC for SES and GMV in voxel clusters attributable to fluid intelligence and mental health proxies. The numbers in the bars report the percent share in the SES-GMV association attributable to fluid intelligence or mental health proxies. The asterisks indicate statistically significant differences with Bonferroni correction after accounting for fluid intelligence or mental health proxies. The clusters were formed with at least 200 voxels showing significant associations at FWE rate of 5% level in the baseline voxel-based morphometry (VBM) results on $PC1_{\mathrm{SES}}$ and $PC2_{\mathrm{SES}}$. The clusters are ordered by the strength of joint associations with $PC1_{\mathrm{SES}}$ and $PC2_{\mathrm{SES}}$. For each cluster, the anatomical location of the peak voxel from the VBM results is indicated at the bottom. See Table S8 for more information about the clusters. Note that the samples used are slightly different from the VBM analysis due to missing data.

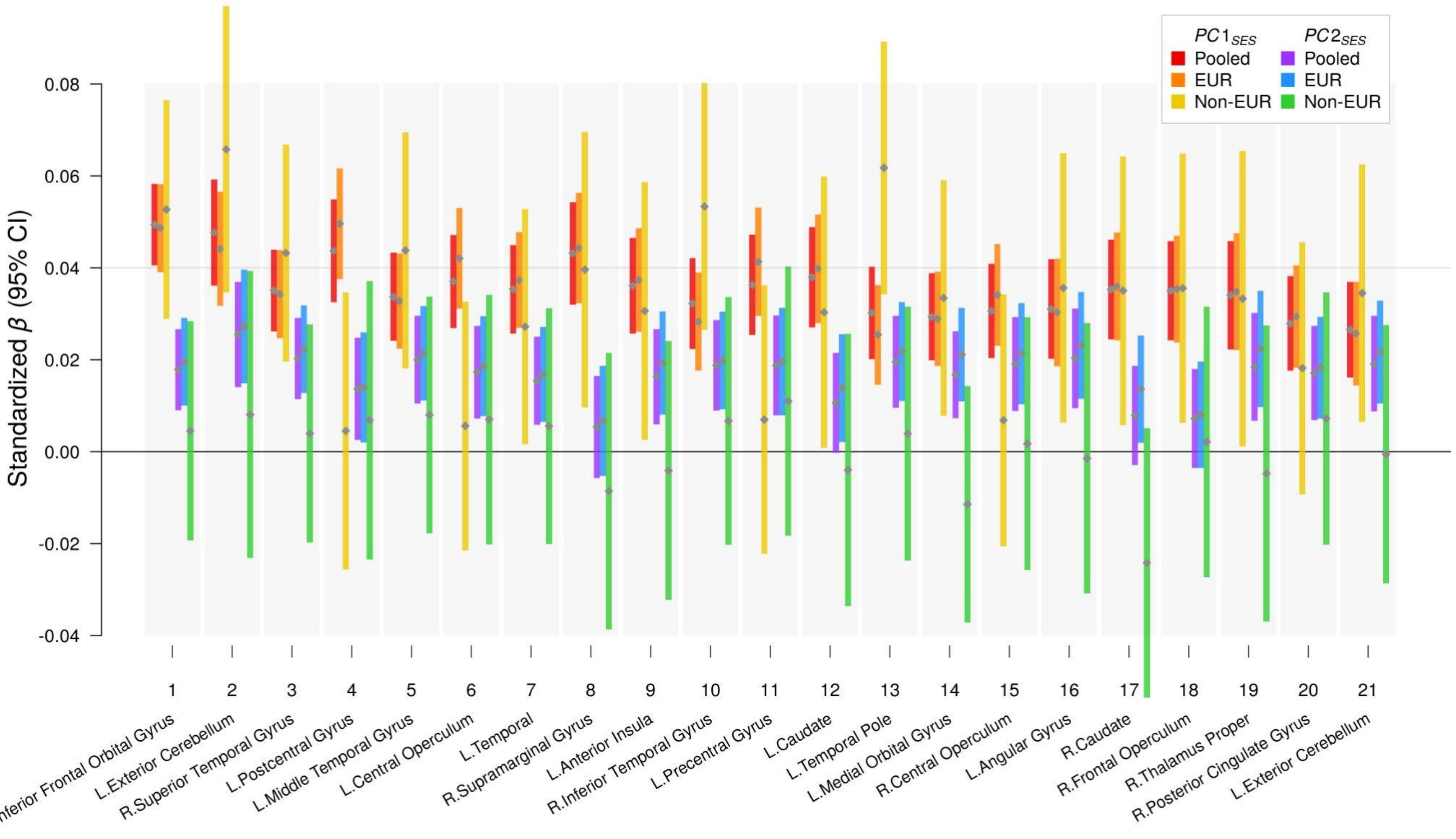

Figure S17. Stratified analysis of samples of European and Non-European ancestries

Results from regressing GMV in each cluster on $PC1_{\mathrm{SES}}$ and $PC2_{\mathrm{SES}}$ with the pooled sample and the stratified sample of European and Non-European genetic ancestry. The standardized coefficient estimates (grey points) are plotted with their uncorrected 95% confidence intervals (color bars). The clusters were formed with at least 200 voxels showing significant associations at FWE rate of 5% level in the baseline voxel-based morphometry (VBM) results on $PC1_{\mathrm{SES}}$ and $PC2_{\mathrm{SES}}$. The clusters are ordered by the strength of joint associations with $PC1_{\mathrm{SES}}$ and $PC2_{\mathrm{SES}}$. For each cluster, the anatomical location of the peak voxel from the VBM results is indicated at the bottom. See Table S8 for more information about the clusters.

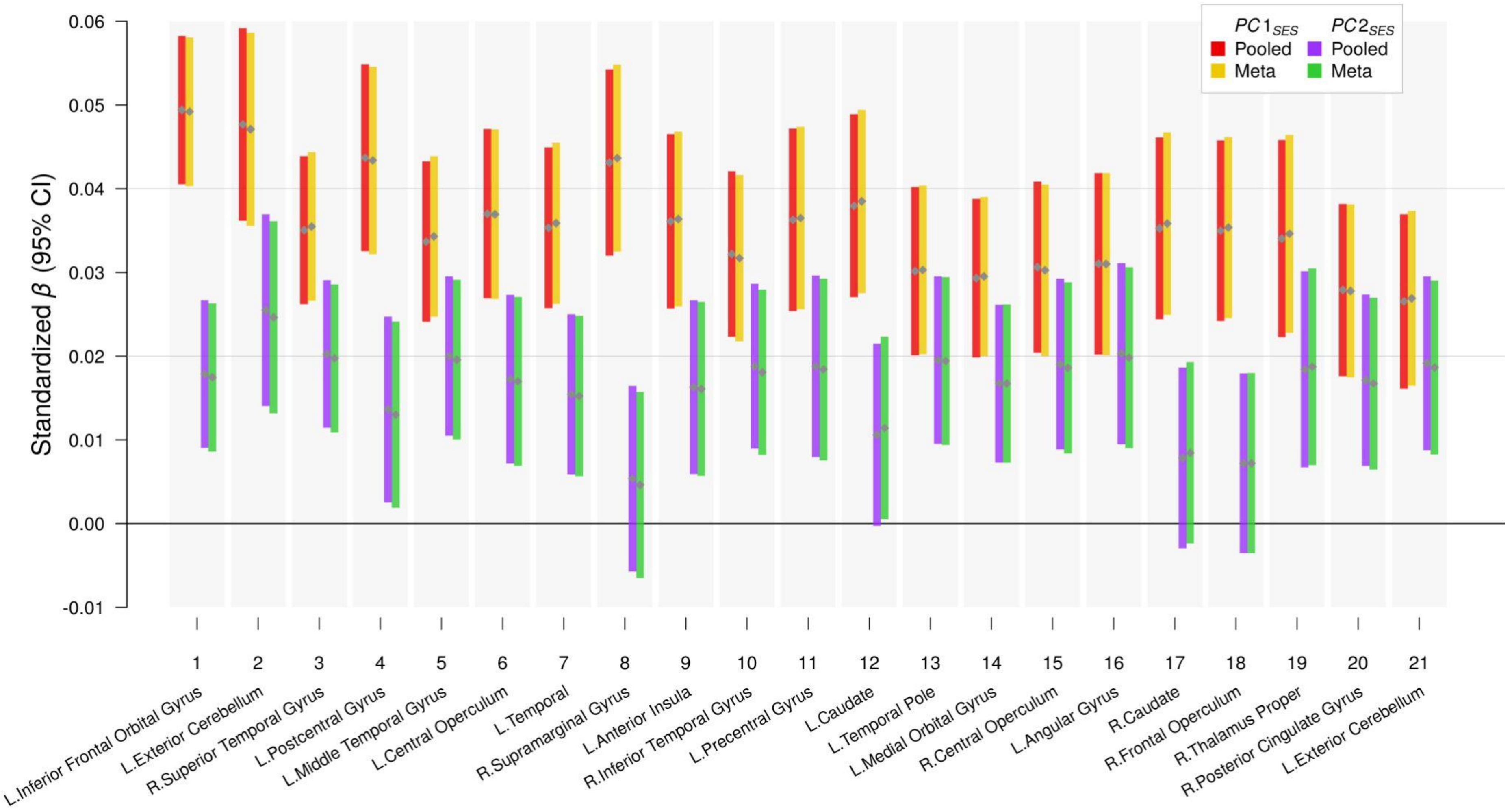

Figure S18. Comparison of meta-analysis and pooled analysis of samples of European and Non-European ancestries

Results from regressing GMV in each cluster on $PC1_{\text{SES}}$ and $PC2_{\text{SES}}$ based on the pooled analysis and the meta-analysis of samples of European and Non-European genetic ancestry. The inverse-variance weights were used for the meta-analysis. The standardized coefficient estimates (grey points) are plotted with their uncorrected 95% confidence intervals (color bars). The clusters were formed with at least 200 voxels showing significant associations at FWE rate of 5% level in the baseline voxel-based morphometry (VBM) results on $PC1_{\text{SES}}$ and $PC2_{\text{SES}}$. The clusters are ordered by the strength of joint associations with $PC1_{\text{SES}}$ and $PC2_{\text{SES}}$. For each cluster, the anatomical location of the peak voxel from the VBM results is indicated at the bottom. See Table S8 for more information about the clusters.

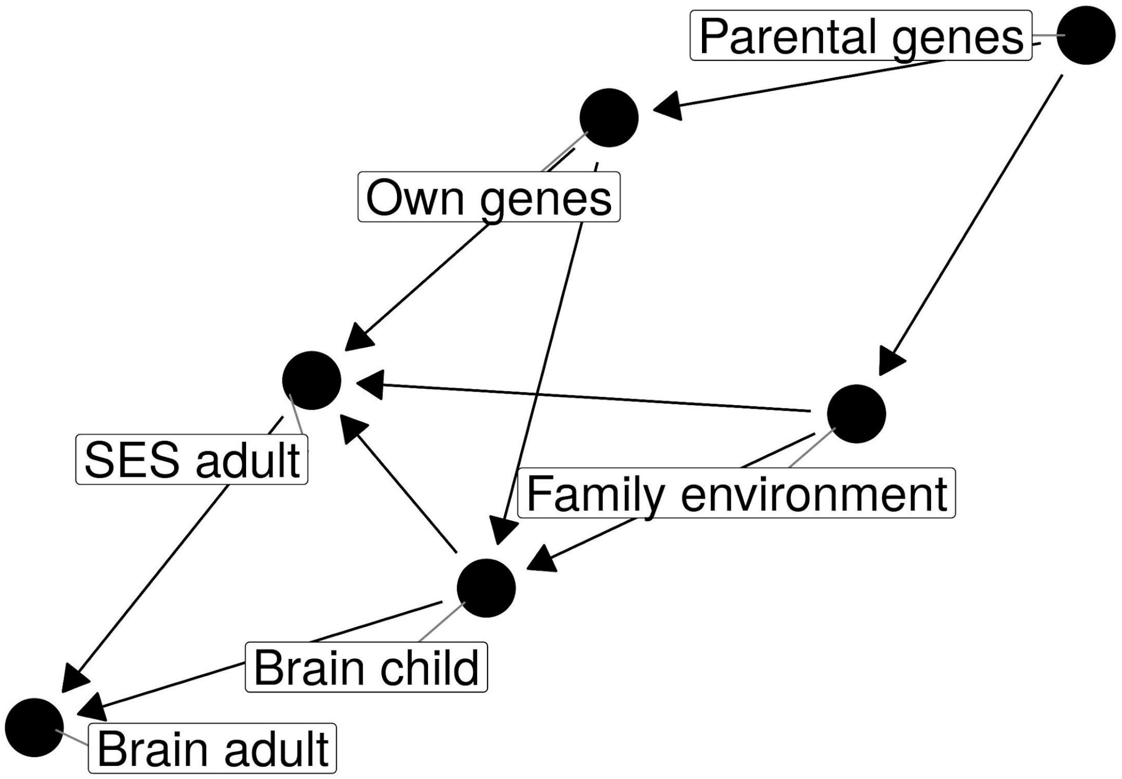

Figure S19. Path diagram of the brain and its link to socioeconomic and genetic factors

The figure shows a directed acyclic graph that illustrates how adulthood brain anatomy can be linked to socioeconomic status, family environments, and genetics.

Other Supplementary Materials for this manuscript include the following:

Data S1: Excel file which contains the source data for Supplementary tables S1-S25.

REFERENCES AND NOTES

1. N. E. Adler, J. Stewart, Health disparities across the lifespan: Meaning, methods, and mechanisms. *Ann. N. Y. Acad. Sci.* **1186**, 5–23 (2010).

2. D. W. Sacks, B. Stevenson, J. Wolfers, The new stylized facts about income and subjective well-being. *Emotion* **12**, 1181–1187 (2012).

3. R. Chetty, M. Stepner, S. Abraham, S. Lin, B. Scuderi, N. Turner, A. Bergeron, D. Cutler, The association between income and life expectancy in the United States, 2001-2014. *JAMA* **315**, 1750–1766 (2016).

4. S. Stringhini, C. Carmeli, M. Jokela, M. Avendaño, P. Muennig, F. Guida, F. Ricceri, A. d'Errico, H. Barros, M. Bochud, M. Chadeau-Hyam, F. Clavel-Chapelon, G. Costa, C. Delpierre, S. Fraga, M. Goldberg, G. G. Giles, V. Krogh, M. Kelly-Irving, R. Layte, A. M. Lasserre, M. G. Marmot, M. Preisig, M. J. Shipley, P. Vollenweider, M. Zins, I. Kawachi, A. Steptoe, J. P. Mackenbach, P. Vineis, M. Kivimäki; LIFEPATH consortium, Socioeconomic status and the 25 × 25 risk factors as determinants of premature mortality: A multicohort study and meta-analysis of 1.7 million men and women. *Lancet* **389**, 1229–1237 (2017).

5. M. Ridley, G. Rao, F. Schilbach, V. Patel, Poverty, depression, and anxiety: Causal evidence and mechanisms. *Science* **370**, eaay0214 (2020).

6. B. S. McEwen, P. J. Gianaros, Central role of the brain in stress and adaptation: Links to socioeconomic status, health, and disease. *Ann. N. Y. Acad. Sci.* **1186**, 190–222 (2010).

7. K. A. Muscatell, Socioeconomic influences on brain function: Implications for health. *Ann. N. Y. Acad. Sci.* **1428**, 14–32 (2018).

8. M. J. Farah, The neuroscience of socioeconomic status: Correlates, causes, and consequences. *Neuron* **96**, 56–71 (2017).

9. E. C. Merz, C. A. Wiltshire, K. G. Noble, Socioeconomic inequality and the developing brain: Spotlight on language and executive function. *Child Dev. Perspect.* **13**, 15–20 (2019).

10. K. G. Noble, M. A. Giebler, The neuroscience of socioeconomic inequality. *Curr. Opin. Behav. Sci.* **36**, 23–28 (2020).

11. K. S. Button, J. P. A. Ioannidis, C. Mokrysz, B. A. Nosek, J. Flint, E. S. J. Robinson, M. R. Munafò, Power failure: Why small sample size undermines the reliability of neuroscience. *Nat. Rev. Neurosci.* **14**, 365–376 (2013).

12. K. B. Walhovd, A. M. Fjell, Y. Wang, I. K. Amlien, A. M. Mowinckel, U. Lindenberger, S. Düzel, D. Bartrés-Faz, K. P. Ebmeier, C. A. Drevon, W. F. C. Baaré, P. Ghisletta, L. B. Johansen, R. A. Kievit, R. N. Henson, K. S. Madsen, L. Nyberg, J. R Harris, C. Solé-Padullés, S. Pudas, Ø. Sørensen, R. Westerhausen, E. Zsoldos, L. Nawijn, T. H. Lyngstad, S. Suri, B. Penninx, O. J. Rogeberg, A. M. Brandmaier, Education and income show heterogeneous relationships to lifespan brain and cognitive differences across European and US cohorts. *Cereb. Cortex* **32**, 839–854 (2021).

13. D. G. Weissman, M. Hatzenbuehler, M. Cikara, D. Barch, K. A. McLaughlin, Antipoverty programs mitigate socioeconomic disparities in brain structure and psychopathology among U.S. youths. *PsyArXiv* 10.31234/osf.io/8nhej (2021).

14. R. J. Herrnstein, C. A. Murray, *The Bell Curve: Intelligence and Class Structure in American Life* (Free Press, 1994).

15. K. L. Miller, F. Alfaro-Almagro, N. K. Bangerter, D. L. Thomas, E. Yacoub, J. Xu, A. J. Bartsch, S. Jbabdi, S. N. Sotiropoulos, J. L. R. Andersson, L. Griffanti, G. Douaud, T. W. Okell, P. Weale, I. Dragonu, S. Garratt, S. Hudson, R. Collins, M. Jenkinson, P. M. Matthews, S. M. Smith, Multimodal population brain imaging in the UK Biobank prospective epidemiological study. *Nat. Neurosci.* **19**, 1523–1536 (2016).

16. C. Bycroft, C. Freeman, D. Petkova, G. Band, L. T. Elliott, K. Sharp, A. Motyer, D. Vukcevic, O. Delaneau, J. O'Connell, A. Cortes, S. Welsh, A. Young, M. Effingham, G. McVean, S. Leslie, N. Allen, P. Donnelly, J. Marchini, The UK Biobank resource with deep phenotyping and genomic data. *Nature* **562**, 203–209 (2018).

17. J. J. Lee, R. Wedow, A. Okbay, E. Kong, O. Maghzian, M. Zacher, T. A. Nguyen-Viet, P. Bowers, J. Sidorenko, R. K. Linnér, M. A. Fontana, T. Kundu, C. Lee, H. Li, R. Li, R. Royer, P. N. Timshel, R. K. Walters, E. A. Willoughby, L. Yengo; 23andMe Research Team; COGENT (Cognitive Genomics Consortium); Social Science Genetic Association Consortium, M. Alver, Y. Bao, D. W. Clark, F. R. Day, N. A. Furlotte, P. K. Joshi, K. E. Kemper, A. Kleinman, C. Langenberg, R. Mägi, J. W. Trampush, S. S. Verma, Y. Wu, M. Lam, J. H. Zhao, Z. Zheng, J. D. Boardman, H. Campbell, J. Freese, K. M. Harris, C. Hayward, P. Herd, M. Kumari, T. Lencz, J. Luan, A. K. Malhotra, A. Metspalu, L. Milani, K. K. Ong, J. R. B. Perry, D. J. Porteous, M. D. Ritchie, M. C. Smart, B. H. Smith, J. Y. Tung, N. J. Wareham, J. F. Wilson, J. P. Beauchamp, D. C. Conley, T. Esko, S. F. Lehrer, P. K. E. Magnusson, S. Oskarsson, T. H. Pers, M. R. Robinson, K. Thom, C. Watson, C. F. Chabris, M. N. Meyer, D. I. Laibson, J. Yang, M. Johannesson, P. D. Koellinger, P. Turley, P. M. Visscher, D. J. Benjamin, D. Cesarini, Gene discovery and polygenic prediction from a genome-wide association study of educational attainment in 1.1 million individuals. *Nat. Genet.* **50**, 1112–1121 (2018).

18. G. Nave, W. H. Jung, R. K. Linnér, J. W. Kable, P. D. Koellinger, Are bigger brains smarter? Evidence from a large-scale preregistered study. *Psychol. Sci.* **30**, 43–54 (2019).

19. J. Cavanagh, R. Krishnadas, G. D. Batty, H. Burns, K. A. Deans, I. Ford, A. McConnachie, A. McGinty, J. S. McLean, K. Millar, N. Sattar, P. G. Shiels, C. Tannahill, Y. N. Velupillai, C. J. Packard, J. McLean, Socioeconomic status and the cerebellar grey matter volume. Data from a well-characterised population sample. *Cerebellum* **12**, 882–891 (2013).

20. N. E. Adler, J. M. Ostrove, Socioeconomic status and health: What we know and what we don't. *Ann. N. Y. Acad. Sci.* **896**, 3–15 (1999).

21. J. Schnittker, Education and the changing shape of the income gradient in health. *J. Health Soc. Behav.* **45**, 286–305 (2004).

22. K. G. Noble, S. M. Houston, N. H. Brito, H. Bartsch, E. Kan, J. M. Kuperman, N. Akshoomoff, D. G. Amaral, C. S. Bloss, O. Libiger, N. J. Schork, S. S. Murray, B. J. Casey, L. Chang, T. M. Ernst, J. A. Frazier, J. R. Gruen, D. N. Kennedy, P. Van Zijl, S. Mostofsky, W. E. Kaufmann, T. Kenet, A.

M. Dale, T. L. Jernigan, E. R. Sowell, Family income, parental education and brain structure in children and adolescents. *Nat. Neurosci.* **18**, 773–778 (2015).

23. D. Rose, D. J. Pevalin, *A Researcher's Guide to the National Statistics Socio-Economic Classification* (SAGE, 2003).

24. J. Mbatchou, L. Barnard, J. Backman, A. Marcketta, J. A. Kosmicki, A. Ziyatdinov, C. Benner, C. O'Dushlaine, M. Barber, B. Boutkov, L. Habegger, M. Ferreira, A. Baras, J. Reid, G. Abecasis, E. Maxwell, J. Marchini, Computationally efficient whole-genome regression for quantitative and binary traits. *Nat. Genet.* **53**, 1097–1103 (2021).

25. A. D. Grotzinger, M. Rhemtulla, R. de Vlaming, S. J. Ritchie, T. T. Mallard, W. D. Hill, H. F. Ip, R. E. Marioni, A. M. McIntosh, I. J. Deary, P. D. Koellinger, K. P. Harden, M. G. Nivard, E. M. Tucker-Drob, Genomic structural equation modelling provides insights into the multivariate genetic architecture of complex traits. *Nat. Hum. Behav.* **3**, 513–525 (2019).

26. T. A. DiPrete, C. A. P. Burik, P. D. Koellinger, Genetic instrumental variable regression: Explaining socioeconomic and health outcomes in nonexperimental data. *Proc. Natl. Acad. Sci. U.S.A.* **115**, E4970–E4979 (2018).

27. F. Morys, M. Dadar, A. Dagher, Association between midlife obesity and its metabolic consequences, cerebrovascular disease, and cognitive decline. *J. Clin. Endocrinol. Metab.* **106**, e4260–e4274 (2021).

28. J. Ludwig, L. Sanbonmatsu, L. Gennetian, E. Adam, G. J. Duncan, L. F. Katz, R. C. Kessler, J. R. Kling, S. T. Lindau, R. C. Whitaker, T. W. McDade, Neighborhoods, obesity, and diabetes—A randomized social experiment. *N. Engl. J. Med.* **365**, 1509–1519 (2011).

29. R. A. Poldrack, A. Kittur, D. Kalar, E. Miller, C. Seppa, Y. Gil, D. S. Parker, F. W. Sabb, R. M. Bilder, The cognitive atlas: Toward a knowledge foundation for cognitive neuroscience. *Front. Neuroinform.* **5**, 17 (2011).

30. J. Dockès, R. A. Poldrack, R. Primet, H. Gözükan, T. Yarkoni, F. Suchanek, B. Thirion, G. Varoquaux, NeuroQuery, comprehensive meta-analysis of human brain mapping. *Elife* **9**, e53385 (2020).

31. L. Raffington, D. Czamara, J. J. Mohn, J. Falck, V. Schmoll, C. Heim, E. B. Binder, Y. L. Shing, Stable longitudinal associations of family income with children's hippocampal volume and memory persist after controlling for polygenic scores of educational attainment. *Dev. Cogn. Neurosci.* **40**, 100720 (2019).

32. B. L. Mitchell, G. Cuéllar-Partida, K. L. Grasby, A. I. Campos, L. T. Strike, L.-D. Hwang, A. Okbay, P. M. Thompson, S. E. Medland, N. G. Martin, M. J. Wright, M. E. Rentería, Educational attainment polygenic scores are associated with cortical total surface area and regions important for language and memory. *Neuroimage* **212**, 116691 (2020).

33. N. Judd, B. Sauce, J. Wiedenhoeft, J. Tromp, B. Chaarani, A. Schliep, B. van Noort, J. Penttilä, Y. Grimmer, C. Insensee, A. Becker, T. Banaschewski, A. L. W. Bokde, E. B. Quinlan, S. Desrivières, H. Flor, A. Grigis, P. Gowland, A. Heinz, B. Ittermann, J.-L. Martinot, M.-L. Paillère Martinot, E. Artiges, F. Nees, D. Papadopoulos Orfanos, T. Paus, L. Poustka, S. Hohmann, S. Millenet, J. H. Fröhner, M. N. Smolka, H. Walter, R. Whelan, G. Schumann, H. Garavan, T. Klingberg, Cognitive and brain development is independently influenced by socioeconomic status and polygenic scores for educational attainment. *Proc. Natl. Acad. Sci. U.S.A.* **117**, 12411–12418 (2020).

34. C. Murray, *Human Diversity: The Biology of Gender, Race, and Class* (Grand Central Publishing, 2020).

35. D. B. Paul, How PKU became a genetic disease, in *Human Heredity in the Twentieth Century*, B. Gausemeier, Ed. (Routledge, 2015), pp. 197–210.

36. C. Jencks, Heredity, environment, and public policy reconsidered. *Am. Sociol. Rev.* **45**, 723–736 (1980).

37. K. P. Harden, P. D. Koellinger, Using genetics for social science. *Nat. Hum. Behav.* **4**, 567–576 (2020).

38. A. Kong, G. Thorleifsson, M. L. Frigge, B. J. Vilhjalmsson, A. I. Young, T. E. Thorgeirsson, S. Benonisdottir, A. Oddsson, B. V. Halldorsson, G. Masson, D. F. Gudbjartsson, A. Helgason, G. Bjornsdottir, U. Thorsteinsdottir, K. Stefansson, The nature of nurture: Effects of parental genotypes. *Science* **359**, 424–428 (2018).

39. A. S. Goldberger, Heritability. *Economica* **46**, 327–347 (1979).

40. M. J. Farah, Socioeconomic status and the brain: Prospects for neuroscience-informed policy. *Nat. Rev. Neurosci.* **19**, 428–438 (2018).

41. M. J. Farah, S. Sternberg, T. A. Nichols, J. T. Duda, T. Lohrenz, Y. Luo, L. Sonnier, S. L. Ramey, R. Montague, C. T. Ramey, Randomized manipulation of early cognitive experience impacts adult brain structure. *J. Cogn. Neurosci.* **33**, 1197–1209 (2021).

42. K. G. Noble, K. Magnuson, L. A. Gennetian, G. J. Duncan, H. Yoshikawa, N. A. Fox, S. Halpern-Meekin, Baby's first years: Design of a randomized controlled trial of poverty reduction in the United States. *Pediatrics* **148**, e2020049702 (2021).

43. T. J. Littlejohns, J. Holliday, L. M. Gibson, S. Garratt, N. Oesingmann, F. Alfaro-Almagro, J. D. Bell, C. Boultwood, R. Collins, M. C. Conroy, N. Crabtree, N. Doherty, A. F. Frangi, N. C. Harvey, P. Leeson, K. L. Miller, S. Neubauer, S. E. Petersen, J. Sellors, S. Sheard, S. M. Smith, C. L. M. Sudlow, P. M. Matthews, N. E. Allen, The UK Biobank imaging enhancement of 100,000 participants: Rationale, data collection, management and future directions. *Nat. Commun.* **11**, 2624 (2020).

44. F. Alfaro-Almagro, M. Jenkinson, N. K. Bangerter, J. L. R. Andersson, L. Griffanti, G. Douaud, S. N. Sotiropoulos, S. Jbabdi, M. Hernandez-Fernandez, E. Vallee, D. Vidaurre, M. Webster, P. McCarthy, C. Rorden, A. Daducci, D. C. Alexander, H. Zhang, I. Dragonu, P. M. Matthews, K. L. Miller, S. M. Smith, Image processing and quality control for the first 10,000 brain imaging datasets from UK Biobank. *Neuroimage* **166**, 400–424 (2018).

45. M. O. Hill, A. J. E. Smith, Principal component analysis of taxonomic data with multi-state discrete characters. *Taxon* **25**, 249–255 (1976).

46. J. Pagès, *Multiple Factor Analysis by Example using R* (CRC Press, 2014).

47. M. Chavent, V. Kuentz-Simonet, A. Labenne, J. Saracco, Multivariate analysis of mixed data: The R package PCAmixdata. arXiv:1411.4911 [stat.CO] (18 November 2017).

48. P.-R. Loh, G. Tucker, B. K. Bulik-Sullivan, B. J. Vilhjálmsson, H. K. Finucane, R. M. Salem, D. I. Chasman, P. M. Ridker, B. M. Neale, B. Berger, N. Patterson, A. L. Price, Efficient Bayesian mixed-model analysis increases association power in large cohorts. *Nat. Genet.* **47**, 284–290 (2015).

49. T. T. Mallard, R. K. Linnér, A. Okbay, A. D. Grotzinger, R. de Vlaming, S. F. W. Meddens, E. M. Tucker-Drob, K. S. Kendler, M. C. Keller, P. D. Koellinger, K. Paige Harden, Not just one p: Multivariate GWAS of psychiatric disorders and their cardinal symptoms reveal two dimensions of cross-cutting genetic liabilities. bioRxiv 603134 [**Preprint**]. 8 September 2020. https://doi.org/10.1101/603134.

50. B. J. Vilhjálmsson, J. Yang, H. K. Finucane, A. Gusev, S. Lindström, S. Ripke, G. Genovese, P.-R. Loh, G. Bhatia, R. Do, T. Hayeck, H.-H. Won; Schizophrenia Working Group of the Psychiatric Genomics Consortium, Discovery, Biology, and Risk of Inherited Variants in Breast Cancer (DRIVE) study, S. Kathiresan, M. Pato, C. Pato, R. Tamimi, E. Stahl, N. Zaitlen, B. Pasaniuc, G. Belbin, E. E. Kenny, M. H. Schierup, P. De Jager, N. A. Patsopoulos, S. McCarroll, M. Daly, S. Purcell, D. Chasman, B. Neale, M. Goddard, P. M. Visscher, P. Kraft, N. Patterson, A. L. Price, Modeling linkage disequilibrium increases accuracy of polygenic risk scores. *Am. J. Hum. Genet.* **97**, 576–592 (2015).

51. F. Privé, J. Arbel, B. J. Vilhjálmsson, LDpred2: Better, faster, stronger. *Bioinformatics* **36**, 5424–5431 (2020).

52. S. McCarthy, S. Das, W. Kretzschmar, O. Delaneau, A. R. Wood, A. Teumer, H. M. Kang, C. Fuchsberger, P. Danecek, K. Sharp, Y. Luo, C. Sidore, A. Kwong, N. Timpson, S. Koskinen, S. Vrieze, L. J. Scott, H. Zhang, A. Mahajan, J. Veldink, U. Peters, C. Pato, C. M. van Duijn, C. E. Gillies, I. Gandin, M. Mezzavilla, A. Gilly, M. Cocca, M. Traglia, A. Angius, J. C. Barrett, D. Boomsma, K. Branham, G. Breen, C. M. Brummett, F. Busonero, H. Campbell, A. Chan, S. Chen, E. Chew, F. S. Collins, L. J. Corbin, G. D. Smith, G. Dedoussis, M. Dorr, A.-E. Farmaki, L.

Ferrucci, L. Forer, R. M. Fraser, S. Gabriel, S. Levy, L. Groop, T. Harrison, A. Hattersley, O. L. Holmen, K. Hveem, M. Kretzler, J. C. Lee, M. McGue, T. Meitinger, D. Melzer, J. L. Min, K. L. Mohlke, J. B. Vincent, M. Nauck, D. Nickerson, A. Palotie, M. Pato, N. Pirastu, M. McInnis, J. B. Richards, C. Sala, V. Salomaa, D. Schlessinger, S. Schoenherr, P. E. Slagboom, K. Small, T. Spector, D. Stambolian, M. Tuke, J. Tuomilehto, L. H. Van den Berg, W. Van Rheenen, U. Volker, C. Wijmenga, D. Toniolo, E. Zeggini, P. Gasparini, M. G. Sampson, J. F. Wilson, T. Frayling, P. I. W. de Bakker, M. A. Swertz, S. McCarroll, C. Kooperberg, A. Dekker, D. Altshuler, C. Willer, W. Iacono, S. Ripatti, N. Soranzo, K. Walter, A. Swaroop, F. Cucca, C. A. Anderson, R. M. Myers, M. Boehnke, M. I. McCarthy, R. Durbin; Haplotype Reference Consortium, A reference panel of 64,976 haplotypes for genotype imputation. *Nat. Genet.* **48**, 1279–1283 (2016).

53. The International HapMap 3 Consortium, Integrating common and rare genetic variation in diverse human populations. *Nature* **467**, 52–58 (2010).

54. A. Okbay, J. P. Beauchamp, M. A. Fontana, J. J. Lee, T. H. Pers, C. A. Rietveld, P. Turley, G.-B. Chen, V. Emilsson, S. F. W. Meddens, S. Oskarsson, J. K. Pickrell, K. Thom, P. Timshel, R. de Vlaming, A. Abdellaoui, T. S. Ahluwalia, J. Bacelis, C. Baumbach, G. Bjornsdottir, J. H. Brandsma, M. Pina Concas, J. Derringer, N. A. Furlotte, T. E. Galesloot, G. Girotto, R. Gupta, L. M. Hall, S. E. Harris, E. Hofer, M. Horikoshi, J. E. Huffman, K. Kaasik, I. P. Kalafati, R. Karlsson, A. Kong, J. Lahti, S. J. van der Lee, C. deLeeuw, P. A. Lind, K.-O. Lindgren, T. Liu, M. Mangino, J. Marten, E. Mihailov, M. B. Miller, P. J. van der Most, C. Oldmeadow, A. Payton, N. Pervjakova, W. J. Peyrot, Y. Qian, O. Raitakari, R. Rueedi, E. Salvi, B. Schmidt, K. E. Schraut, J. Shi, A. V. Smith, R. A. Poot, B. St Pourcain, A. Teumer, G. Thorleifsson, N. Verweij, D. Vuckovic, J. Wellmann, H.-J. Westra, J. Yang, W. Zhao, Z. Zhu, B. Z. Alizadeh, N. Amin, A. Bakshi, S. E. Baumeister, G. Biino, K. Bønnelykke, P. A. Boyle, H. Campbell, F. P. Cappuccio, G. Davies, J.-E. De Neve, P. Deloukas, I. Demuth, J. Ding, P. Eibich, L. Eisele, N. Eklund, D. M. Evans, J. D. Faul, M. F. Feitosa, A. J. Forstner, I. Gandin, B. Gunnarsson, B. V. Halldórsson, T. B. Harris, A. C. Heath, L. J. Hocking, E. G. Holliday, G. Homuth, M. A. Horan, J.-J. Hottenga, P. L. de Jager, P. K. Joshi, A. Jugessur, M. A. Kaakinen, M. Kähönen, S. Kanoni, L. Keltigangas-Järvinen, L. A. L. M. Kiemeney, I. Kolcic, S. Koskinen, A. T. Kraja, M. Kroh, Z. Kutalik, A. Latvala, L. J. Launer, M. P. Lebreton, D. F. Levinson, P. Lichtenstein, P. Lichtner, D. C. M. Liewald; LifeLines Cohort Study, A. Loukola, P. A. Madden, R. Mägi, T. Mäki-Opas, R. E. Marioni, P. Marques-Vidal, G. A. Meddens, G. McMahon,

C. Meisinger, T. Meitinger, Y. Milaneschi, L. Milani, G. W. Montgomery, R. Myhre, C. P. Nelson, D. R. Nyholt, W. E. R. Ollier, A. Palotie, L. Paternoster, N. L. Pedersen, K. E. Petrovic, D. J. Porteous, K. Räikkönen, S. M. Ring, A. Robino, O. Rostapshova, I. Rudan, A. Rustichini, V. Salomaa, A. R. Sanders, A.-P. Sarin, H. Schmidt, R. J. Scott, B. H. Smith, J. A. Smith, J. A. Staessen, E. Steinhagen-Thiessen, K. Strauch, A. Terracciano, M. D. Tobin, S. Ulivi, S. Vaccargiu, L. Quaye, F. J. A. van Rooij, C. Venturini, A. A. E. Vinkhuyzen, U. Völker, H. Völzke, J. M. Vonk, D. Vozzi, J. Waage, E. B. Ware, G. Willemsen, J. R. Attia, D. A. Bennett, K. Berger, L. Bertram, H. Bisgaard, D. I. Boomsma, I. B. Borecki, U. Bültmann, C. F. Chabris, F. Cucca, D. Cusi, I. J. Deary, G. V. Dedoussis, C. M. van Duijn, J. G. Eriksson, B. Franke, L. Franke, P. Gasparini, P. V. Gejman, C. Gieger, H.-J. Grabe, J. Gratten, P. J. F. Groenen, V. Gudnason, P. van der Harst, C. Hayward, D. A. Hinds, W. Hoffmann, E. Hyppönen, W. G. Iacono, B. Jacobsson, M.-R. Järvelin, K.-H. Jöckel, J. Kaprio, S. L. R. Kardia, T. Lehtimäki, S. F. Lehrer, P. K. E. Magnusson, N. G. Martin, M. McGue, A. Metspalu, N. Pendleton, B. W. J. H. Penninx, M. Perola, N. Pirastu, M. Pirastu, O. Polasek, D. Posthuma, C. Power, M. A. Province, N. J. Samani, D. Schlessinger, R. Schmidt, T. I. A. Sørensen, T. D. Spector, K. Stefansson, U. Thorsteinsdottir, A. R. Thurik, N. J. Timpson, H. Tiemeier, J. Y. Tung, A. G. Uitterlinden, V. Vitart, P. Vollenweider, D. R. Weir, J. F. Wilson, A. F. Wright, D. C. Conley, R. F. Krueger, G. Davey Smith, A. Hofman, D. I. Laibson, S. E. Medland, M. N. Meyer, J. Yang, M. Johannesson, P. M. Visscher, T. Esko, P. D. Koellinger, D. Cesarini, D. J. Benjamin, Genome-wide association study identifies 74 loci associated with educational attainment. *Nature* **533**, 539–542 (2016).

55. J. B. Burt, M. Helmer, M. Shinn, A. Anticevic, J. D. Murray, Generative modeling of brain maps with spatial autocorrelation. *Neuroimage* **220**, 117038 (2020).

56. G. Aydogan, R. Daviet, R. Karlsson Linnér, T. A. Hare, J. W. Kable, H. R. Kranzler, R. R. Wetherill, C. C. Ruff, P. D. Koellinger, G. Nave, Genetic underpinnings of risky behaviour relate to altered neuroanatomy. *Nat. Hum. Behav.* **5**, 787–794 (2021).

57. R. Daviet, G. Aydogan, K. Jagannathan, N. Spilka, P. D. Koellinger, H. R. Kranzler, G. Nave, R. R. Wetherill, Associations between alcohol consumption and gray and white matter volumes in the UK Biobank. *Nat. Commun.* **13**, 1175 (2022).

58. M. L. Anderson, Multiple inference and gender differences in the effects of early intervention: A reevaluation of the abecedarian, perry preschool, and early training projects. *J. Am. Stat. Assoc.* **103**, 1481–1495 (2008).

59. F. Alfaro-Almagro, P. McCarthy, S. Afyouni, J. L. R. Andersson, M. Bastiani, K. L. Miller, T. E. Nichols, S. M. Smith, Confound modelling in UK Biobank brain imaging. *Neuroimage* **224**, 117002 (2020).

60. P. Turley, R. K. Walters, O. Maghzian, A. Okbay, J. J. Lee, M. A. Fontana, T. A. Nguyen-Viet, R. Wedow, M. Zacher, N. A. Furlotte; 23andMe Research Team; Social Science Genetic Association Consortium, P. Magnusson, S. Oskarsson, M. Johannesson, P. M. Visscher, D. Laibson, D. Cesarini, B. M. Neale, D. J. Benjamin, Multi-trait analysis of genome-wide association summary statistics using MTAG. *Nat. Genet.* **50**, 229–237 (2018).

61. T. Nichols, S. Hayasaka, Controlling the familywise error rate in functional neuroimaging: A comparative review. *Stat. Methods Med. Res.* **12**, 419–446 (2003).

62. A. M. Winkler, G. R. Ridgway, M. A. Webster, S. M. Smith, T. E. Nichols, Permutation inference for the general linear model. *Neuroimage* **92**, 381–397 (2014).

63. D. Freedman, D. Lane, A nonstochastic interpretation of reported significance levels. *J. Bus. Econ. Stat.* **1**, 292–298 (1983).

64. D. P. MacKinnon, J. L. Krull, C. M. Lockwood, Equivalence of the mediation, confounding and suppression effect. *Prev. Sci.* **1**, 173–181 (2000).

65. J. M. Wooldridge, *Econometric Analysis of Cross Section and Panel Data* (MIT Press, 2010).

66. A. F. Alexander-Bloch, H. Shou, S. Liu, T. D. Satterthwaite, D. C. Glahn, R. T. Shinohara, S. N. Vandekar, A. Raznahan, On testing for spatial correspondence between maps of human brain structure and function. *Neuroimage* **178**, 540–551 (2018).

67. S. Greenland, J. Pearl, J. M. Robins, Causal diagrams for epidemiologic research. *Epidemiology* **10**, 37–48 (1999).

68. I. Shrier, R. W. Platt, Reducing bias through directed acyclic graphs. *BMC Med. Res. Methodol.* **8**, 70 (2008).

69. P. W. G. Tennant, E. J. Murray, K. F. Arnold, L. Berrie, M. P. Fox, S. C. Gadd, W. J. Harrison, C. Keeble, L. R. Ranker, J. Textor, G. D. Tomova, M. S. Gilthorpe, G. T. H. Ellison, Use of directed acyclic graphs (DAGs) to identify confounders in applied health research: Review and recommendations. *Int. J. Epidemiol.* (2020).

70. S. Selzam, S. J. Ritchie, J.-B. Pingault, C. A. Reynolds, P. F. O'Reilly, R. Plomin, Comparing within- and between-family polygenic score prediction. *Am. J. Hum. Genet.* **105**, 351–363 (2019).

71. J. Yang, B. Benyamin, B. P. McEvoy, S. Gordon, A. K. Henders, D. R. Nyholt, P. A. Madden, A. C. Heath, N. G. Martin, G. W. Montgomery, M. E. Goddard, P. M. Visscher, Common SNPs explain a large proportion of the heritability for human height. *Nat. Genet.* **42**, 565–569 (2010).

72. S. H. Lee, J. Yang, M. E. Goddard, P. M. Visscher, N. R. Wray, Estimation of pleiotropy between complex diseases using single-nucleotide polymorphism-derived genomic relationships and restricted maximum likelihood. *Bioinformatics* **28**, 2540–2542 (2012).

73. L. M. Evans, R. Tahmasbi, S. I. Vrieze, G. R. Abecasis, S. Das, S. Gazal, D. W. Bjelland, T. R. de Candia; Haplotype Reference Consortium, M. E. Goddard, B. M. Neale, J. Yang, P. M. Visscher, M. C. Keller, Comparison of methods that use whole genome data to estimate the heritability and genetic architecture of complex traits. *Nat. Genet.* **50**, 737–745 (2018).

74. P.-R. Loh, G. Bhatia, A. Gusev, H. K. Finucane, B. K. Bulik-Sullivan, S. J. Pollack; Schizophrenia Working Group of Psychiatric Genomics Consortium, T. R. de Candia, S. H. Lee, N. R. Wray, K. S. Kendler, M. C. O'Donovan, B. M. Neale, N. Patterson, A. L. Price, Contrasting genetic architectures of schizophrenia and other complex diseases using fast variance-components analysis. *Nat. Genet.* **47**, 1385–1392 (2015).

75. S. E. Collins, Associations between socioeconomic factors and alcohol outcomes. *Alcohol Res.* **38**, 83–94 (2016).

76. A. Fry, T. J. Littlejohns, C. Sudlow, N. Doherty, L. Adamska, T. Sprosen, R. Collins, N. E. Allen, Comparison of sociodemographic and health-related characteristics of UK biobank participants with those of the general population. *Am. J. Epidemiol.* **186**, 1026–1034 (2017).
